# Electrochemical‐Induced Ring Transformation of Cyclic α‐(*ortho*‐Iodophenyl)‐β‐oxoesters

**DOI:** 10.1002/chem.201905570

**Published:** 2020-01-30

**Authors:** Julia Strehl, Christoph Kahrs, Thomas Müller, Gerhard Hilt, Jens Christoffers

**Affiliations:** ^1^ Institut für Chemie Carl von Ossietzky Universität Oldenburg 26111 Oldenburg Germany

**Keywords:** annulation, electrosynthesis, medium-ring compounds, reduction, ring expansion

## Abstract

Cyclic α‐(*ortho*‐iodophenyl)‐β‐oxoesters were converted in a ring‐expanding transformation to furnish benzannulated cycloalkanone carboxylic esters. The reaction sequence started by electrochemical reduction of the iodoarene moiety. In a mechanistic rationale, the resulting carbanionic species was adding to the carbonyl group under formation of a strained, tricyclic benzocyclobutene intermediate, which underwent carbon–carbon bond cleavage and rearrangement of the carbon skeleton by retro‐aldol reaction. The scope of the reaction sequence was investigated by converting cyclic oxoesters with different ring sizes yielding benzocycloheptanone, ‐nonanone and ‐decanone derivatives in moderate to good yields. Furthermore, acyclic starting materials and cyclic compounds carrying additional substituents on the iodophenyl ring were submitted to this reaction sequence. The starting materials for this transformation are straightforwardly obtained by conversion of β‐oxoesters with phenyliodobis(trifluoroacetate).

## Graphical abstract

**Electrosynthesis**: Benzannulated cycloalkanone carboxylic esters are obtained by ring‐expansion of cyclic α‐(*ortho*‐iodophenyl)‐β‐oxoesters. The reaction sequence starts with the electrochemical reduction of the iodoarene moiety. After nucleophilic addition to the carbonyl group the transformation proceeds via a tricyclic benzocyclobutene intermediate, which finally undergoes C−C bond cleavage by a retro‐aldol reaction (see scheme).

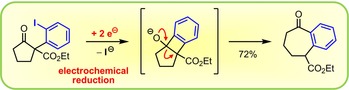

Due to inherent entropic and enthalpic factors, the construction of seven‐membered^1^ and larger (medium) sized rings^2^ defines a challenging task in synthetic organic chemistry. A common strategy towards such synthetic targets is the ring‐expansion of more readily available compounds with five or six‐membered rings.^3^ As an illustrative example, Stoltz and co‐workers^4^ introduced the ring‐expansion of β‐oxoester **1 a** with an aryne furnishing benzocycloheptanone derivative **2 a** (Scheme 1). After addition of the enolate of oxoester **1 a** to the aryne, the reaction proceeds via intermediates **3** and **4**. The aryne was generated in situ from trimethylsilylphenyltriflate and excess CsF. As an alternative to this protocol we envisioned to generate carbanionic species **3** from iodo‐compound **5 a** by an electrochemical reduction.^5^ Electrosynthesis is an attractive concept with steadily growing importance, because it can potentially reduce the amount of waste on one hand by avoiding spent redox reagents. On the other hand, renewable energy can be used to contribute to more sustainable conversions.^6^ The preparation of the starting materials of this new synthetic concept, α‐(*ortho*‐iodophenyl)‐β‐oxoesters **5**, was recently introduced by Shafir and co‐workers^7^ by conversion of β‐oxoesters **1** with PhI(O_2_CCF_3_)_2_ (PIFA).

**Scheme 1:**
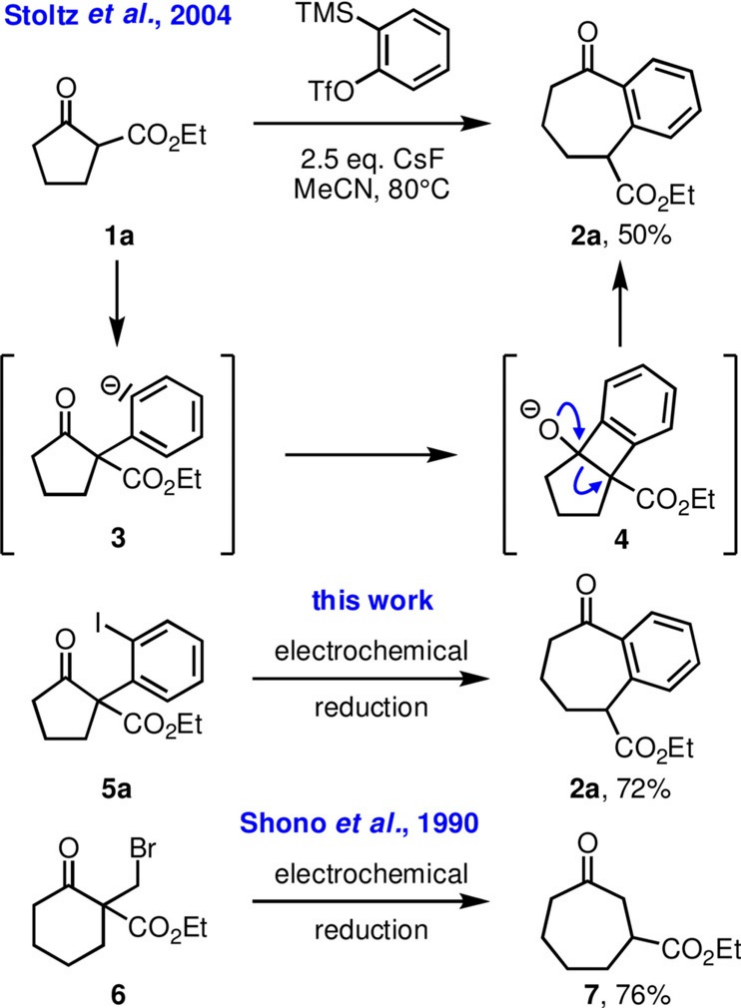
Previously reported ring‐transformation of oxoester **1 a** with an in situ generated aryne^4^ via intermediates **3** and **4**. Entry into the same reaction sequence leading to product **2 a** by electrochemical reduction of iodoarene **5 a** (this work) and electrochemical ring‐enlargement of oxoester **6** by cathodic reduction. Conditions: 3 equiv TMSCl, *n*Et_4_NOTos, DMF, 23 °C, 0.2 A, 4 F mol^−1^, Pb cathode, carbon anode.^8^

We started the screening program for optimal reaction conditions based on a literature report by Shono et al. (Scheme 1), who have developed a cathodic ring‐enlargement reaction of α‐(bromomethyl)‐β‐oxoesters like compound **6** by application of DMF as the solvent and three equivalents of TMSCl as an additive to activate the carbonyl group of the ketone to facilitate the nucleophilic addition.^8^ As a cathode material, we have chosen leaded bronze, which was recently recommended as an innovative material for reductive dehalogenations.^9^ Initially, we have used *n*Bu_4_NClO_4_ as a conducting salt and a current of 10 mA and achieved 54 % yield of compound **5 a** (Table 1, entry 1). Change of the conducting salt revealed that the anion had almost no influence on the yield. Other cations, however, lowered the yield. Therefore, we have chosen *n*Bu_4_NBr in all further experiments, since with this supporting electrolyte the byproduct profile was least complicated (entry 2). Variations of the cathode (graphite, glassy carbon, Cu, Pt, Pb) and anode (glassy carbon, Pt) materials did not improve the yields. Investigation of the Lewis acidic additive (TMSOTf, Ti(OEt)_4_, TiCl_4_, CeCl_3_, LaCl_3_, AlCl_3_, InBr_3_, ZnCl_2_, ZnI_2_, BF_3_ **⋅**OEt_2_) also gave no improvements; however, without or with only one equivalent of TMSCl, no conversion was achieved and with five equivalents of TMSCl the yield was lower. Survey of other solvents confirmed DMF to be optimal (MeCN, DMPU, NMP, and DMA gave lower yields, with 1,4‐dioxane and THF no conductivity was achieved). The first and only significant improvement was achieved, when the electric current was lowered (entries 3 and 4). With 8, 6.5, or 5 mA the yield was raised to 71–72 %. Upon further lowering (2.5 mA, entry 5) it dropped to 43 %. In favor of a high reaction rate it was kept at 8 mA for all further optimizations. Variation of the reaction temperature (entries 6 and 7) as well as lowering the concentration of the conducting salt (entry 8) lowered the yield. For more details on the screening program see the Supporting Information.

**Table 1:** Selected examples of variation to the reaction conditions.

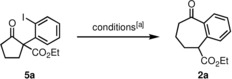		
Entry	Change from initial conditions^[b]^	Yield of **2 a** [%]^[c]^
1	no change	54
2	*n*Bu_4_NBr (*c=*0.3 mol l ^−1^) as conducting salt^[d]^	55
3	***I*** **=8 mA** ^[e]^	**72**
4	*I*=6.5 mA or 5 mA	71
5	*I*=2.5 mA	43
6	*T=*0 °C	36
7	*T=*40 °C	41
8	*c*(*n*Bu_4_NBr)=0.2 mol l ^−1^	60

[a] Reactions were performed in a divided cell on a 0.25 mmol scale with a substrate concentration of 36 mmol l ^−1^. [b] Initial conditions: 3.0 equiv TMSCl, 0.3 mol l ^−1^ *n*Bu_4_NClO_4_, DMF, 23 °C, 10 mA, 2.0 F mol^−1^, CuSn_17_Pb (leaded bronze) cathode, graphite anode. [c] Yield determined by GLC of the unpurified reaction mixture with mesitylene as internal standard. [d] This change was kept for entries 3–7. [e] This change was kept for entries 6–8.

With optimized reaction conditions in hand, we applied various substrates of type **5** to investigate the scope and limitations of the electrochemical‐induced rearrangement reaction. First, we have investigated the ring size of the starting materials (Table 2). Whereas the yield of the benzocycloheptanone **2 a** was good, it was somewhat lower for the benzannulated products with nine‐ (compound **2 c**, 64 %) and 10‐membered rings (**2 d**, 45 %). Attempts of converting the cyclohexanone derivative **5 b** to product **2 b** were never successful; instead, varying amounts of the protodeiodinated compound **8** could be detected in the reaction mixture. Interestingly, this type of starting material was also investigated by Stoltz in the aryne‐induced ring‐expansion and the corresponding rearrangement was not observed as well.^4^ The reason for this failure, however, remains unclear. Moreover, the conversion of indanone and tetralone derivatives **5 e** and **5 f** also gave rather complex reaction mixtures. However, in the latter case the desired product dibenzocyclooctanone derivative **5 f** could be isolated in low yield (10 %). The acyclic starting materials **5 g** and **5 h** could be converted to acetophenone derivative **2 g** (54 %) and benzophenone derivative **2 h** (18 %). Finally, cyclopentanone derivatives **5 i**–**m** carrying additional substituents at the α‐(*ortho*‐iodophenyl)‐moiety were prepared and converted to respective benzocycloheptanone derivatives **2 i**–**m**. The yields range from 44 to 59 % almost independently from the electronic nature and position of the additional residue. Interestingly, a fluoro substituent in compound **5 j** is tolerated adjacent to the proposed carbanionic center of the intermediate, thus competing aryne formation seems not to take place, albeit product **2 j** was formed in 55 % yield. While all these conversions were performed on a 0.25 mmol scale, we have performed the reaction of compound **5 a** also on a scale of 1 mmol and obtained product **2 a** with a slightly lowered yield of 56 %, which might be due to the doubled concentration of the substrate **5 a** (36 vs. 71 mmol L^−1^).

**Table 2:** Scope of the transformation with various α‐(*ortho*‐iodophenyl)‐β‐oxoesters **5**; yields refer to isolated and purified products.

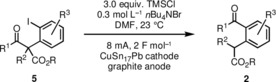
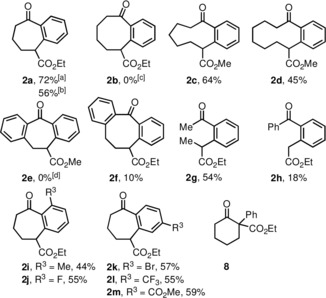

[a] The reaction was performed on a 0.25 mmol scale. [b] The reaction was performed on a 1 mmol scale. [c] Protodeiodination product **8** was formed and isolated in 7 % yield. [d] Unspecified decomposition.

The starting materials **5 a**–**g** of this study were accessed from the β‐oxoesters **1 a**–**g** 10 following the original report^7^ with a stoichiometric amount of PIFA (**9 a**, R^3^=H) and TFAA (trifluoroacetic anhydride) in a mixture of MeCN and TFA (trifluoroacetic acid; Scheme 2; see the Supporting Information). An exception was compound **5 h**, which was prepared by TiCl_4_‐mediated Claisen‐condensation from ethyl (2‐iodophenylacetate) and benzoyl chloride.^11^ Compounds **5 i**–**m** were prepared accordingly from substituted PIFA‐derivatives **9 i**–**m**. The latter were obtained by oxidation of the corresponding iodobenzene derivatives with oxone by following a literature protocol.^12^ As also observed by others,^13^ donor‐substituted PIFA reagents (e.g. with R^3^=OMe) cannot be obtained by this procedure.

**Scheme 2:**
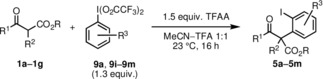
Preparation of starting materials **5 a**–**m** from oxoesters **1 a**–**g** and PIFA **9 a** and PIFA derivatives **9 i**–**m**.

Regarding the mechanism of the overall transformation, it could be either proposed, that **5 a** is reduced to the corresponding phenyl radical, which then enters the reaction sequence by attack to the carbonyl group. More likely, however, is the formation of a phenyl anion derivative (cf. species **3** in Scheme 1), which then proceeds to formation of the tricylic intermediate **4**. As evidence for the latter process we have prepared the Grignard reagent from compound **5 a** by using a protocol introduced by Knochel et al. (Scheme 3)^14^ and indeed obtained the product **2 a** in 17 % yield. Furthermore, when converting compound **5 a** to the respective phenyl radical by using *n*Bu_3_SnH and AIBN, only the protodeiodinated product **10** could be detected by NMR spectroscopy. There is actually literature precedence on the electrochemical reduction of aryliodides. The initial electron transfer to a radical intermediate was reported to be relatively slow, while the second electron transfer to the anion was much faster.^15^

**Scheme 3:**
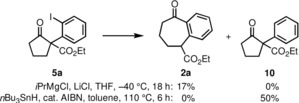
Control experiments for the ionic or radical transformation of starting material **5 a**.

Crucial in the overall process seems the role of TMSCl. We propose it to activate the endocyclic carbonyl group (intermediate **11** in Scheme 4). Nucleophilic addition of the phenyl anion to the activated carbonyl group gives intermediate **12** with CO_2_Et and OTMS groups in relative *cis*‐configuration. The results of DFT calculations at the M06‐2X/Def2‐TZVP level (see the Supporting Information) revealed that this step is exergonic (Δ*G* ^298^(**11**→**12**)=−268 kJ mol^−1^) and the reaction proceeds almost without barrier (Δ*G* ^298≠^(**11**→**12**)=+8 kJ mol^−1^). Initially, we had assumed that the TMS group would then be transferred to the ester‐oxygen atom while the C−C‐bond is cleaved in the retro‐aldol reaction, thus the ketene–silyl acetal **13** would be formed. Although this process is slightly exergonic (Δ*G* ^298^(**12**→**13**)=−6 kJ mol^−1^), it is kinetically highly unfavorable. It should proceed via a transition state with a pentacoordinated silicon atom in a trigonal bipyramidal coordination environment in which the two oxygen atoms adopt the axial positions. This arrangement cannot be realized within the strained tricyclic framework.^16^ Therefore, we suggest, that the ring transformation is finalized after hydrolytic workup, which could be supported by calculations: The concerted ring‐opening/proton‐transfer reaction that yields the (*Z*)‐isomer of enol **15** is essentially thermoneutral (Δ*G* ^298^(**14**→**15**)=+1 kJ mol^−1^) and the subsequent exergonic tautomerization step drives the reaction to completion (Δ*G* ^298^(**15**→**2 a′**)=−82 kJ mol^−1^). The proton transfer to the ester‐carbonyl oxygen atom is assisted by one water molecule, which allows the proton transfer to proceed via two hydrogen bridges with a relative small barrier (Δ*G* ^298≠^(**14**→**15**)=+61 kJ mol^−1^).^17^

**Scheme 4:**
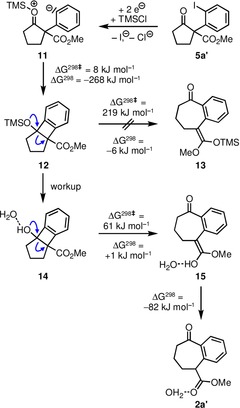
Mechanistic proposal for the transformation of compound **5 a′** by two‐electron reduction in the presence of TMSCl to product **2 a′**. DFT calculations were performed at M06‐2X/Def2‐TZVP with the methyl instead of the ethyl esters; species **14**, **15**, and **2 a′** were calculated as hydrogen‐bonded monohydrates.

In summary, a novel reaction pathway was realized for the generation of the oxoesters of type **2** from iodobenzene derivatives **5** in the presence of TMSCl by a formal 1,3‐acyl‐shift. The reaction sequence starts by an electrochemical reduction of the iodobenzene derivative **5** and proceeds by nucleophilic addition of the carbanion to the carbonyl group followed by C−C bond cleavage by retro‐aldol reaction. From cyclic oxoesters as starting materials, a ring enlargement by two carbon atoms is achieved, thus elegant access to medium‐sized ring compounds is attained.

## Conflict of interest

The authors declare no conflict of interest.

## Supporting information

As a service to our authors and readers, this journal provides supporting information supplied by the authors. Such materials are peer reviewed and may be re‐organized for online delivery, but are not copy‐edited or typeset. Technical support issues arising from supporting information (other than missing files) should be addressed to the authors.

Supplementary

Supplementary
