# Supplementary material for: Electrochemical‐Induced Ring Transformation of Cyclic α‐(ortho‐Iodophenyl)‐β‐oxoesters
Source: Chemistry. 2020 Jan 30;26(15):3222–5. doi: 10.1002/chem.201905570 (PMC7155071; doi:10.1002/chem.201905570)
Supplement: Supplementary file 1 — Supplementary [file CHEM-26-3222-s001.pdf]

CHEMISTRY
A European Journal

Supporting Information

Electrochemical-Induced Ring Transformation of Cyclic α-(*ortho*-Iodophenyl)-β-oxoesters

Julia Strehl, Christoph Kahrs, Thomas Müller,* Gerhard Hilt,* and Jens Christoffers*[a]

chem_201905570_sm_miscellaneous_information.pdf
chem_201905570_sm_transition_state_14_to_15.gif

Supporting Information

1. General Information S2
2. Electrosynthesis S3
3. α-(*ortho*-Iodophenylation) of β-Oxo Esters **1** S12
4. Synthesis of PIFA Derivatives **9** S20
5. Computational Details S22
6. References S26
7. NMR-Spectra of all Reported Compounds S27
8. GLC of Compounds **2a**, **8** and **2c**–**2m** S61

1. General Information

All solvents were commercially available and were distilled under reduced pressure prior to use. DMF was dried over CaH_2, distilled and stored over 3 Å molecular sieves. All other solvents were dried over 3 Å molecular sieves. Methyl 1-oxoindane-2-carboxylate (**1e**)[S1] and ethyl 1-oxo-1,2,3,4-tetrahydronaphthalene-2-carboxylate (**1f**)[S1] were prepared according to literature procedures. All other starting materials or reagents were purchased from commercial suppliers without further purification. If water or air sensitive compounds were used, the experiments were carried out in heat dried glassware using conventional Schlenk techniques under nitrogen atmosphere. Electrochemical reactions were carried out using an AIM-TTI Instruments MX100T power supply. These reactions were performed in a divided H-type cell (Figure S1), equipped with stirring bars, septums, inert gas supply and a $CuSn_{17}Pb$ cathode (4.7 cm^2) and a graphite anode (3.5 cm^2).

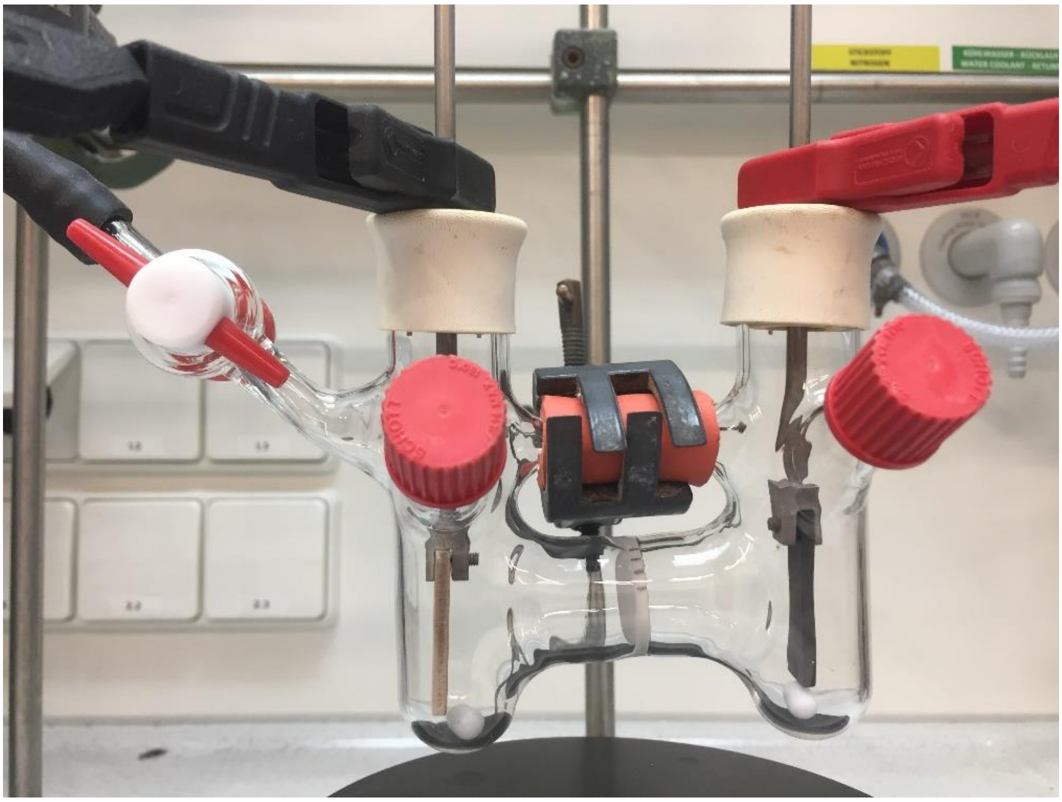

Figure S1. H-type electrolysis cell, divided by a glas frit (G4); left: $CuSn_{17}Pb$ cathode; right: graphite anode.

NMR spectra were recorded on Bruker Avance 300 (300 MHz), Avance III (500 MHz) or Avance DRX (500 MHz) instruments. Chemical shifts were reported in parts per million (ppm). The spectra were referenced to residual solvent peaks. The IR spectra were obtained with a Shimadzu IRSpirit with a QATR-S cell. The wave numbers λ^{-1} were quoted in recipro-

cal centimeters (cm^{-1}). MS and HRMS spectra of products were obtained with a Waters Q-TOF Premier (ESI, pos. mode) or Thermo Scientific DFS (EI) spectrometers.
Flash column chromatography was carried out using Machery-Nagel SiO_2 60 (40–63 µm). Thin layer chromatography was carried out on Merck TLC plates coated with SiO_2 60 F_{254} with fluorescence indicator.

2. Electrosynthesis

2.1 Optimization of the Reaction Conditions

Table S1. Optimization of the reaction conditions.

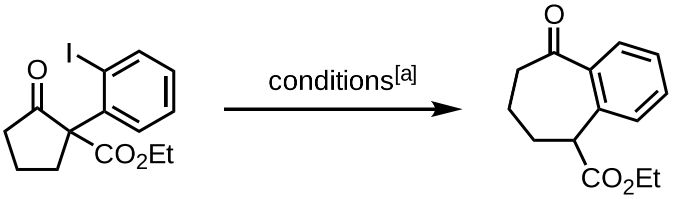

5a **2a**

Entry	variation from initial conditions[b]	Yield of **2a**[c]
	screening of conductive salts	
1	*n*Bu_4NBr	55%
2	*n*Bu_4NClO_4	54%
3	Et_4NOTos	52%
4	*n*Bu_4NPF_6	40%
5	$LiClO_4$	17%
6	*n*Bu_4NBF_4	51%
	electrode materials	
7	graphite cathode	14%
8	glassy carbon cathode	0%
9	Cu cathode	7%
10	Pt cathode	0%
11	Pb cathode[d]	67%
11	glassy carbon anode	43%
12	Pt anode	31%

Table S1 continued.

	additives	
13	no additive	0%
14	$Ti(OEt)_4$	29%
15	$CeCl_3$	17%
16	$ZnCl_2$	20%
17	$LaCl_3$	5%
18	$BF_3 \cdot OEt_2$[d]	52%
19	TMSOTf[d]	64%
20	$TiCl_4$[d]	0%
21	$AlCl_3$[d]	51%
22	$InBr_3$[d]	0%
23	ZnI_2[d]	39%
	electric current	
24	**I = 8 mA**	**72%**
25	I = 6.5 mA or 5 mA	71%
26	I = 2.5 mA	43%
	solvents	
27	1,4-dioxane	no conductivity
28	DMPU	29%
29	NMP	40%
30	THF	no conductivity
31	DMA[d]	0%

[a] Reactions were performed in a divided cell on a 0.25 mmol scale with a substrate concentration of 36 mmol L^{-1}. [b] Initial conditions: 3.0 equiv. TMSCl, 0.3 mol L^{-1} $n$$Bu_4Br$, DMF, 23°C, 10 mA, 2.0 F mol^{-1}, $CuSn_{17}Pb$ (leaded bronze) cathode, graphite anode. [c] Yield determined by GLC of the unpurified reaction mixture with mesitylene as internal standard. [d] Performed at 8 mA.

2.2 General procedure A (GPA) for the electrochemical conversion of *ortho*-iodophenyl compounds 5

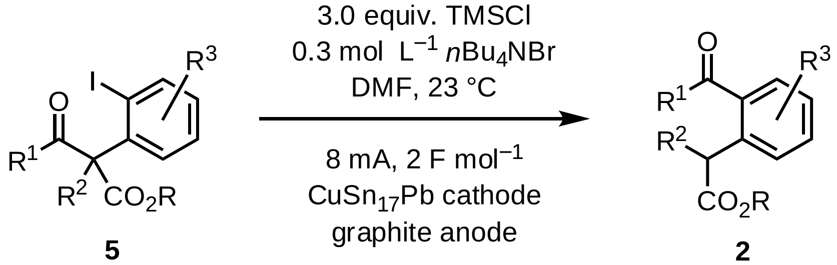

First of all, nBu$_4$NBr (677 mg, 2.10 mmol) was weighed into each chamber of the cell. The reaction tube was then evacuated, refilled with inert gas, and *abs.* DMF [7 mL/chamber, c(nBu$_4$NBr) = 0.3 mol L^{-1}] was added to each chamber. Subsequently, TMSCl (3.0 equiv.) and the corresponding α-(*ortho*-iodophenyl)-β-oxoester **5** (1.0 equiv.) were added into the cathodic chamber and the reaction mixture was electrolyzed under constant current (8 mA, 2.0 F mol^{-1}). The reaction mixture was diluted with sat. aq. NH_4Cl solution (40 mL) and extracted with Et_2O (3 × 20 mL). The combined organic layers were dried ($MgSO_4$) and filtered. After evaporation, the residue was submitted to column chromatography to furnish the respective products **2**.

2.3 Ethyl 9-Oxo-6,7,8,9-tetrahydro-5*H*-benzocycloheptene-5-carboxylate (2a)

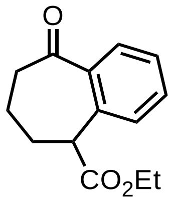

(1) According to GPA, oxoester **5a** (90 mg, 0.25 mmol) and TMSCl (82 mg, 0.75 mmol) were converted to furnish product **2a** (42 mg, 0.18 mmol, 72%) as colorless oil after column chromatography (SiO_2, *n*-pentane/Et_2O 5:1, R_f = 0.20).

(2) Fourfold scale: First of all, nBu$_4$NBr (1.35 g, 4.2 mmol) was weighed into each chamber of the cell. The reaction tube was then evacuated, refilled with inert gas, and *abs.* DMF [14 mL/chamber, c(nBu$_4$NBr) = 0.3 mol L^{-1}] was added to each chamber. Subsequently, TMSCl (326 mg, 3.00 mmol) and the corresponding oxoester **5a** (358 mg, 1.00 mmol) were added into the cathodic chamber and the reaction mixture was electrolyzed under constant current (8 mA, 2.0 F mol^{-1}). The reaction mixture was diluted with sat. aq. NH_4Cl solution (60 mL) and extracted with Et_2O (3 × 25 mL). The combined organic layers were dried ($MgSO_4$) and filtered. After evaporation, the residue was submitted to column chromatography (SiO_2, *n*-pentane/Et_2O 5:1, R_f = 0.20) to furnish the product **2a** (129 mg, 0.56 mmol, 56%) as colorless oil.

(3) By iodine-metal exchange reaction: LiCl (31 mg, 0.73 mmol) was dried under high vacuum at 100 °C for 1 h. Under nitrogen atmosphere and at 0 °C, *i*PrMgCl (2 mol L^{-1} in THF, 0.31 mL, 0.62 mmol) was added dropwise. After stirring for 30 min, anhydrous THF (2 mL) was added. At –40 °C, a solution of oxoester **5a** (0.20 g, 0.56 mmol) in anhydrous THF (5 mL) was added dropwise. The mixture was warmed up to ambient temperature and further stirred for 18 h, then hydrochloric acid (1 mol L^{-1}, 10 mL) was added. The mixture was extracted with MTBE (3 x 20 mL). The combined organic layers were dried ($MgSO_4$) and filtered. The solvent was removed *in vacuo* and the crude product purified by column chromatography (SiO_2, hexanes/MTBE 1:1, R_f = 0.42) to give compound **2a** (22 mg, 95 µmol, 17%) as a colorless oil.

^{1}H NMR (300 MHz, $CDCl_3$): δ = 7.64 (dd, J = 7.6 Hz, J = 1.4 Hz, 1 H), 7.44 (dt, J = 7.5 Hz, J = 1.5 Hz, 1 H), 7.35 (dt, J = 7.5 Hz, J = 1.5 Hz, 1 H), 7.17 (d, J = 7.6 Hz, 1 H), 4.24–4.13 (m, 2 H), 3.99 (dd, J = 7.6 Hz, J = 5.5 Hz, 1 H), 2.80–2.69 (m, 1 H), 2.66–2.55 (m, 1 H), 2.42–2.31 (m, 1 H), 2.12–2.00 (m, 1 H), 1.90–1.77 (m, 2 H), 1.22 (t, J = 7.1 Hz, 3 H) ppm. ^{13}C{^{1}H} NMR (125 MHz, $CDCl_3$): δ = 206.0 (C), 173.3 (C), 139.7 (C), 136.7 (C), 132.0 (CH), 128.78 (CH), 128.76 (CH), 127.8 (CH), 61.3 (CH_2), 49.0 (CH), 41.0 (CH_2), 28.5 (CH_2), 20.4 (CH_2), 14.2 (CH_3) ppm. The spectroscopic data are in accordance with literature values.[S2]

2.4 Ethyl 2-oxo-1-phenylcyclohexane-1-carboxylate (8)

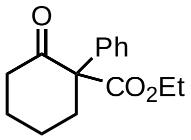

According to GPA, oxoester **5b** (155 mg, 0.420 mmol) and TMSCl (136 mg, 1.25 mmol) were converted to furnish product **8** (18 mg, 73 µmol, 7%) as colorless oil after column chromatography (SiO_2, *n*-pentane/Et_2O 5:1, R_f = 0.37). ^{1}H NMR (500 MHz, $CDCl_3$): δ = 7.38–7.34 (m, 2 H), 7.32–7.28 (m, 1 H), 7.25–7.21 (m, 2 H), 4.26–4.18 (m, 2 H), 2.80–2.74 (m, 1 H), 2.60–2.54 (m, 2 H), 2.40–2.32 (m, 1 H), 2.03–1.95 (m, 1 H), 1.86–1.77 (m, 3 H), 1.23 (t, J = 7.1 Hz, 3 H) ppm. ^{13}C{^{1}H} NMR (125 MHz, $CDCl_3$): δ = 206.8 (C), 171.4 (C), 136.9 (C), 128.5 (2 CH), 127.9 (2 CH), 127.7 (CH), 66.6 (C), 61.8 (CH_2), 41.0 (CH_2), 35.5 (CH_2), 27.8 (CH_2), 22.3 (CH_2), 14.1 (CH_3) ppm. The spectroscopic data are in accordance with literature values.[S3]

2.5 Methyl 11-oxo-6,7,8,9,10,11-hexahydro-5*H*-benzocyclononene-5-carboxylate (2c)

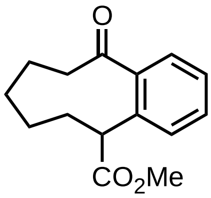

According to GPA, oxoester **5c** (186 mg, 0.500 mmol) and TMSCl (163 mg, 1.50 mmol) were converted to furnish product **2c** (78 mg, 0.32 mmol, 64%) as colorless oil after column chromatography (SiO_2, *n*-pentane/EtOAc 20:1, R_f = 0.18). ^{1}H NMR (500 MHz, $CDCl_3$): δ = 7.39–7.34 (m, 1 H), 7.31–7.27 (m, 2 H), 7.23–7.27 (m, 1 H), 3.95 (dd, *J* = 11.8 Hz, *J* = 4.5 Hz, 1 H), 3.66 (s, 3 H), 2.93–2.86 (m, 1 H), 2.82–2.75 (m, 1 H), 2.05–1.96 (m, 1 H), 1.93–1.83 (m, 2 H), 1.70–1.58 (m, 2 H), 1.58–1.48 (m, 1 H), 1.40–1.30 (m, 1 H), 1.13–1.02 (m, 1 H) ppm. ^{13}C{^{1}H} NMR (125 MHz, $CDCl_3$): δ = 211.3 (C), 174.4 (C), 143.2 (C), 134.8 (C), 130.1 (CH), 127.8 (CH), 127.1 (CH), 124.8 (CH), 52.2 (CH_3), 46.0 (CH), 43.5 (CH_2), 32.3 (CH_2), 26.0 (CH_2), 25.3 (CH_2), 23.7 (CH_2) ppm. The spectroscopic data are in accordance with literature values.[S2]

2.6 Methyl 12-oxo-5,6,7,8,9,10,11,12-octahydrobenzocyclodecene-5-carboxylate (2d)

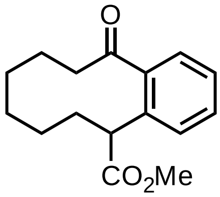

According to GPA, oxoester **5d** (104 mg, 0.269 mmol) and TMSCl (88 mg, 0.81 mmol) were converted to furnish product **2d** (31 mg, 0.12 mmol, 44%) as colorless oil after column chromatography (SiO_2, *n*-pentane/Et_2O 10:1, R_f = 0.08). ^{1}H NMR (500 MHz, $CDCl_3$): δ = 7.45–7.38 (m, 2 H), 7.34–7.27 (m, 2 H), 4.43–4.34 (m, 1 H), 3.70 (s, 3 H), 3.23–3.17 (m, 1 H), 2.55–2.48 (m, 1 H), 1.83–1.73 (m, 3 H), 1.58–1.50 (m, 1 H), 1.44–1.14 (m, 5 H), 0.61–0.50 (m, 1 H) ppm. ^{13}C{^{1}H} NMR (125 MHz, $CDCl_3$): δ = 210.5 (C), 175.1 (C), 141.3 (C), 136.7 (C), 131.1 (CH), 128.3 (CH), 126.6 (CH), 125.8 (CH), 52.2 (CH_3), 44.3 (CH_2), 42.0 (CH), 32.3 (CH_2), 28.2 (CH_2), 22.7 (CH_2), 22.0 (CH_2), 20.9 (CH_2) ppm. IR (ATR): λ^{-1} = 2937 (m), 2851 (m), 1727 (s), 1674 (s), 1600 (w), 1571 (w), 1487 (w), 1249 (m), 1441 (m), 1414 (m), 1347 (m), 1324 (m), 1302 (w), 1267 (s), 1210 (s), 1186 (s), 1148 (m), 1107 (m), 1097 (m), 1028 (m), 1015 (m), 994 (m), 944 (m), 861 (m), 797 (m), 759 (s), 743 (s), 636 (m), 583 (m), 547 (m) cm^{-1}. MS (EI, 70 eV): m/z (%) 260 (11) [M^+], 201 (13), 200 (46), 161 (20), 157 (24), 144 (16), 131 (19), 115 (17), 103 (13), 86 (19), 84 (31), 77 (13), 55 (13), 51 (28), 49 (100). HRMS (EI, 70 eV): calcd. 260.1407 (for $C_{16}H_{20}O_3^+$), found 260.1408 [M^+].

2.7 Ethyl 12-oxo-5,6,7,12-tetrahydrodibenzo[a,d]cyclooctadiene-5-carboxylate (2f)

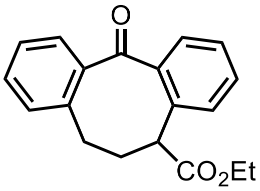

According to GPA, oxoester **5f** (163 mg, 0.388 mmol) and TMSCl (130 mg, 1.20 mmol) were converted to furnish product **2f** (11 mg, 37 μmol, 10%) as colorless oil after column chromatography (SiO_2, *n*-pentane/Et_2O 10:1, R_f = 0.16). ^1H NMR (500 MHz, $CDCl_3$): δ = 8.21 (dd, *J* = 8.0 Hz, *J* = 1.4 Hz, 1 H), 7.93 (dd, *J* = 7.9 Hz, *J* = 1.4 Hz, 1 H), 7.55–7.50 (m, 2 H), 7.44–7.38 (m, 2 H), 7.23–7.18 (m, 2 H), 4.19–4.01 (m, 2 H), 3.85 (dd, *J* = 12.0 Hz, *J* = 4.6 Hz, 1 H), 2.74–2.50 (m, 2 H), 2.15–2.07 (m, 1 H), 2.06–1.97 (m, 1 H), 1.16 (t, *J* = 7.1 Hz, 3 H) ppm. $^{13}C\{^1H\}$ NMR (125 MHz, $CDCl_3$): δ = 194.3 (C), 173.2 (C), 141.4 (C), 140.8 (C), 139.1 (C), 136.9 (C), 133.8 (CH), 132.9 (CH), 131.3 (CH), 130.9 (CH), 130.8 (CH), 127.6 (CH), 127.1 (CH), 126.1 (CH), 61.1 (CH_2), 45.3 (CH), 35.6 (CH_2), 30.7 (CH_2), 14.2 (CH_3) ppm. The spectroscopic data are in accordance with literature values.[S2]

2.8 Ethyl 2-(2-acetylphenyl)propanoate (2g)

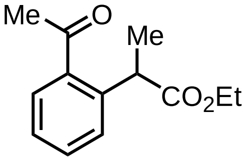

According to GPA, oxoester **5g** (176 mg, 0.508 mmol) and TMSCl (166 mg, 1.52 mmol) were converted to furnish product **2g** (59 mg, 0.27 mmol, 54%) as colorless oil after column chromatography (SiO_2, *n*-pentane/EtOAc 20:1, R_f = 0.14). ^1H NMR (500 MHz, $CDCl_3$): δ = 7.66 (d, *J* = 7.8 Hz, 1 H), 7.45 (t, *J* = 7.6 Hz, 1 H), 7.39 (d, *J* = 7.7 Hz, 1 H), 7.32 (t, *J* = 7.4 Hz, 1 H), 4.41 (q, *J* = 7.2 Hz, 1 H), 4.16–4.05 (m, 2 H), 2.59 (s, 3 H), 1.50 (d, *J* = 7.2 Hz, 3 H), 1.18 (t, *J* = 7.1 Hz, 3 H) ppm. $^{13}C\{^1H\}$ NMR (125 MHz, $CDCl_3$): δ = 202.2 (C), 174.6 (C), 140.3 (C), 137.8 (C), 131.8 (CH), 129.1 (CH), 128.7 (CH), 126.8 (CH), 60.7 (CH_2), 41.7 (CH), 29.8 (CH_3), 18.3 (CH_3), 14.2 (CH_3) ppm. The spectroscopic data are in accordance with literature values.[S2]

2.9 Ethyl 2-(2-benzoylphenyl)acetate (2h)

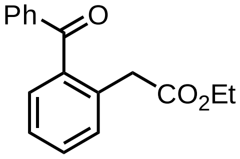

According to GPA, oxoester **5h** (125 mg, 0.317 mmol) and TMSCl (104 mg, 0.957 mmol) were converted to furnish product **2h** (16 mg, 60 μmol, 18%) as colorless oil after column

chromatography (SiO_2, *n*-pentane/Et_2O 10:1, R_f = 0.27). ^{1}H NMR (500 MHz, $CDCl_3$): δ = 7.84–7.80 (m, 2 H), 7.58 (t, J = 7.4 Hz, J = 1.3 Hz, 1 H), 7.50–7.43 (m, 3 H), 7.41–7.36 (m, 2 H), 7.33 (dt, J = 7.5 Hz, J = 1.2 Hz, 1 H), 4.02 (q, J = 7.1 Hz, 2 H), 3.88 (s, 2 H), 1.11 (t, J = 7.1 Hz, 3 H) ppm. ^{13}C{^{1}H} NMR (125 MHz, $CDCl_3$): δ = 198.1 (C), 171.4 (C), 138.5 (C), 138.0 (C), 134.2 (C), 133.0 (CH), 131.9 (CH), 131.0 (CH), 130.5 (2 CH), 130.1 (CH), 128.4 (2 CH), 126.6 (CH), 61.0 (CH_2), 39.0 (CH_2), 14.2 (CH_3) ppm. The spectroscopic data are in accordance with literature values.[S4]

2.10 Ethyl 1-methyl-9-oxo-6,7,8,9-tetrahydro-5*H*-benzocycloheptene-5-carboxylate (2i)

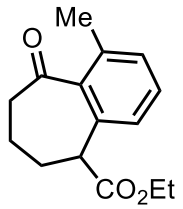

According to GPA, oxoester **5i** (108 mg, 0.290 mmol) and TMSCl (95 mg, 0.87 mmol) were converted to furnish product **2i** (32 mg, 0.13 mmol, 45%) as colorless oil after column chromatography (SiO_2, *n*-pentane/Et_2O 5:1, R_f = 0.27). ^{1}H NMR (500 MHz, $CDCl_3$): δ = 7.28–7.23 (m, 1 H), 7.16 (d, J = 7.7 Hz, 1 H), 6.96 (d, J = 7.7 Hz, 1 H), 4.17 (q, J = 7.2 Hz, 2 H), 3.82 (dd, J = 9.1 Hz, J = 5.8 Hz, 1 H), 2.64 (ddd, J = 17.7 Hz, J = 7.0 Hz, J = 3.4 Hz, 1 H), 2.52 (ddd, J = 17.6 Hz, J = 11.0 Hz, J = 3.4 Hz, 1 H), 2.31 (s, 3 H), 2.27–2.19 (m, 1 H), 2.02–1.94 (m, 1 H), 1.87–1.77 (m, 1 H), 1.74–1.65 (m, 1 H), 1.22 (t, J = 7.2 Hz, 3 H) ppm. ^{13}C{^{1}H} NMR (125 MHz, $CDCl_3$): δ = 209.4 (C), 173.2 (C), 139.2 (C), 135.8 (C), 134.9 (C), 130.3 (CH), 130.3 (CH), 124.8 (CH), 61.2 (CH_2), 48.2 (CH), 42.0 (CH_2), 28.4 (CH_2), 20.8 (CH_2), 20.0 (CH_3), 14.2 (CH_3) ppm. IR (ATR): λ^{-1} = 2934 (w), 2869 (w), 1729 (s), 1687 (s), 1594 (m), 1464 (m), 1372 (w), 1320 (w), 1242 (m), 1186 (m), 1096 (w), 1066 (w), 1028 (m), 983 (w), 910 (m), 874 (w), 840 (w), 786 (m), 740 (m), 576 (w) cm^{-1}. MS (EI, 70 eV): m/z (%) 246 (90) [M^+], 218 (22), 201 (24), 200 (85), 189 (77), 173 (78), 161 (24), 157 (20), 146 (41), 145 (100), 144 (33), 129 (22), 115 (45), 84 (23), 49 (38). HRMS (EI, 70 eV): calcd. 246.1250 (for $C_{15}H_{18}O_3^+$), found 246.1259 [M^+].

2.11 Ethyl 1-fluoro-9-oxo-6,7,8,9-tetrahydro-5*H*-benzocycloheptene-5-carboxylate (2j)

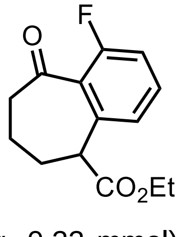

According to GPA, oxoester **5j** (83 mg, 0.22 mmol) and TMSCl (72 mg, 0.66 mmol) were converted to furnish product **2j** (29 mg, 0.12 mmol, 55%) as colorless oil after column chromatography (SiO_2, *n*-pentane/Et_2O 5:1, R_f = 0.24). ^{1}H NMR (500 MHz, $CDCl_3$): δ = 7.42–7.30

(m, 1 H), 7.06 (t, J = 9.0 Hz, 1 H), 6.95 (d, J = 7.7 Hz, 1 H), 4.24–4.10 (m, 2 H), 3.89 (dd, J = 7.8 Hz, J = 5.7 Hz, 1 H), 2.73 (ddd, J = 17.1 Hz, J = 7.4 Hz, J = 3.8 Hz, 1 H), 2.59 (ddd, J = 17.0 Hz, J = 9.8 Hz, J = 4.0 Hz, 1 H), 2.39–2.24 (m, 1 H), 2.10–1.95 (m, 1 H), 1.93–1.73 (m, 2 H), 1.21 (t, J = 7.1 Hz, 3 H) ppm. $^{13}C\{^1H\}$ NMR (125 MHz, $CDCl_3$): δ = 203.0 (C), 172.6 (C), 159.3 (d, J_{CF} = 254.5 Hz, CF), 137.5 (d, J_{CF} = 1.6 Hz, C), 132.2 (d, J_{CF} = 9.2 Hz, CH), 128.1 (d, J_{CF} = 13.5 Hz, C), 123.8 (d, J_{CF} = 3.2 Hz, CH), 115.8 (d, J_{CF} = 23.3 Hz, CH), 61.5 (CH_2), 48.8 (CH), 42.1 (CH_2), 28.5 (CH_2), 20.9 (CH_2), 14.2 (CH_3) ppm. $^{19}F\{^1H\}$ NMR (470 MHz, $CDCl_3$): δ = –116.64 ppm. IR (ATR): λ^{-1} = 2936 (m), 2872 (w), 1729 (s), 1696 (s), 1610 (m), 1576 (m), 1457 (m), 1372 (m), 1320 (w), 1244 (s), 1179 (s), 1096 (w), 1066 (w), 1026 (m), 1000 (m), 923 (m), 913 (m), 874 (w), 795 (m), 734 (m), 684 (w), 597 (w), 516 (w) cm^{-1}. MS (EI, 70 eV): m/z (%) 250 (68) [M^+], 204 (53), 193 (25), 177 (54), 176 (50), 165 (20), 149 (100), 147 (18), 109 (25), 101 (30), 86 (21), 84 (36), 51 (17), 49 (53). HRMS (EI, 70 eV): calcd. 250.1000 (for $C_{14}H_{15}FO_3^+$), found 250.0998 [M^+].

2.12 Ethyl 3-bromo-9-oxo-6,7,8,9-tetrahydro-5*H*-benzocycloheptene-5-carboxylate (2k)

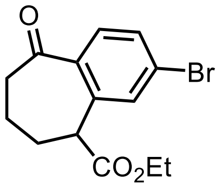

According to GPA, oxoester **5k** (161 mg, 0.368 mmol) and TMSCl (120 mg, 1.10 mmol) were converted to furnish product **2k** (64 mg, 0.21 mmol, 57%) as colorless oil after column chromatography [SiO_2, *n*-pentane/Et_2O 10:1→5:1, R_f(10:1) = 0.17]. ^1H NMR (500 MHz, $CDCl_3$): δ = 7.53–7.47 (m, 2 H), 7.37–7.34 (m, 1 H), 4.22–4.14 (m, 2 H), 3.94 (t, J = 6.3 Hz, 1 H), 2.77–2.69 (m, 1 H), 2.64–2.56 (m, 1 H), 2.42–2.35 (m, 1 H), 2.10–2.01 (m, 1 H), 1.87–1.80 (m, 2 H), 1.22 (t, J = 7.2 Hz, 3 H) ppm. $^{13}C\{^1H\}$ NMR (125 MHz, $CDCl_3$): δ = 204.7 (C), 172.7 (C), 138.6 (C), 138.4 (C), 132.0 (CH), 131.0 (CH), 130.5 (CH), 126.5 (C), 61.6 (CH_2), 48.9 (CH), 40.8 (CH_2), 28.3 (CH_2), 20.2 (CH_2), 14.2 (CH_3) ppm. IR (ATR): λ^{-1} = 2937 (m), 2870 (w), 1727 (s), 1683 (s), 1584 (s), 1558 (w), 1446 (m), 1397 (w), 1369 (w), 1321 (w), 1253 (m), 1183 (s), 1092 (m), 1023 (m), 983 (m), 916 (w), 821 (m), 770 (w), 731 (w), 657 (w), 574 (w), 527 (w) cm^{-1}. MS (EI, 70 eV): m/z (%) 310 (55) [M^+], 264 (50), 255 (25), 239 (32), 238 (38), 236 (35), 225 (18), 209 (40), 158 (20), 130 (100), 129 (50), 115 (35), 102 (90), 55 (22). HRMS (EI, 70 eV): calcd. 310.0199 (for $C_{14}H_{15}BrO_3^+$), found 310.0196 [M^+].

2.13 Ethyl 9-oxo-3-(trifluoromethyl)-6,7,8,9-tetrahydro-5*H*-benzocycloheptene-5-carboxylate (2l)

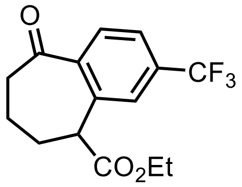

According to GPA, oxoester **5l** (111 mg, 0.260 mmol) and TMSCl (85 mg, 0.78 mmol) were converted to furnish product **2l** (43 mg, 0.14 mmol, 55%) as colorless oil after column chromatography [SiO_2, *n*-pentane/Et_2O 10:1→5:1, R_f(10:1) = 0.15]. ^{1}H NMR (300 MHz, $CDCl_3$): δ = 7.74 (d, J = 8.0 Hz, 1 H), 7.63 (d, J = 7.9 Hz, 1 H), 7.49–7.40 (m, 1 H), 4.25–4.14 (m, 2 H), 4.04 (t, J = 6.2 Hz, 1 H), 2.84–2.71 (m, 1 H), 2.70–2.58 (m, 1 H), 2.47–2.37 (m, 1 H), 2.18–2.00 (m, 1 H), 1.95–1.80 (m, 2 H), 1.23 (t, J = 7.2 Hz, 3 H) ppm. $^{13}C\{^1H\}$ NMR (125 MHz, $CDCl_3$): δ = 204.9 (C), 172.6 (C), 142.7 (C), 137.3 (C), 133.4 (q, J_{CF} = 32.6 Hz, C), 129.4 (CH), 126.1 (q, J_{CF} = 3.6 Hz, CH), 124.8 (q, J_{CF} = 3.4 Hz, CH), 123.7 (q, J_{CF} = 273.9 Hz, CF_3), 61.7 (CH_2), 49.2 (CH), 41.0 (CH_2), 28.4 (CH_2), 20.4 (CH_2), 14.1 (CH_3) ppm. $^{19}F\{^1H\}$ NMR (470 MHz, $CDCl_3$): δ = –63.02 ppm. IR (ATR): λ^{-1} = 2937 (m), 2874 (w), 1729 (s), 1689 (s), 1577 (w), 1494 (w), 1449 (m), 1417 (m), 1372 (w), 1329 (s), 1303 (m), 1253 (m), 1166 (s), 1124 (s), 1084 (s), 1069 (m), 987 (m), 919 (w), 899 (w), 886 (w), 836 (m), 736 (w), 703 (w), 650 (w), 574 (w), 529 (w) cm^{-1}. MS (EI, 70 eV): m/z (%) 300 (77) [M^+], 281 (28), 272 (17), 254 (80), 243 (50), 227 (46), 226 (66), 215 (23), 199 (100), 171 (21), 159 (22), 151 (42), 84 (17), 51(16), 49 (48). HRMS (EI, 70 eV): calcd. 300.0968 (for $C_{15}H_{15}O_3F_3^+$), found 300.0963 [M^+].

2.14 9-Ethyl 2-methyl-5-oxo-6,7,8,9-tetrahydro-5*H*-benzocycloheptene-2,9-dicarboxylate (2m)

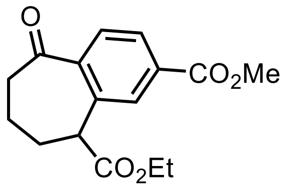

According to GPA, oxoester **5m** (93 mg, 0.22 mmol) and TMSCl (73 mg, 0.67 mmol) were converted to furnish product **2m** (38 mg, 0.13 mmol, 59%) as colorless oil after column chromatography [SiO_2, *n*-pentane/Et_2O 5:1→3:1, R_f(5:1) = 0.21]. ^{1}H NMR (500 MHz, $CDCl_3$): δ = 8.00 (dd, J = 8.0 Hz, J = 1.6 Hz, 1 H), 7.87 (d, J = 1.4 Hz, 1 H), 7.67 (d, J = 8.0 Hz, 1 H), 4.22–4.13 (m, 2 H), 4.04 (t, J = 6.1 Hz, 1 H), 3.92 (s, 3 H), 2.80–2.72 (m, 1 H), 2.67–2.59 (m, 1 H), 2.46–2.38 (m, 1 H), 2.12–2.04 (m, 1 H), 1.88–1.81 (m, 2 H), 1.21 (t, J = 7.1 Hz, 3 H) ppm. $^{13}C\{^1H\}$ NMR (125 MHz, $CDCl_3$): δ = 205.4 (C), 172.9 (C), 166.3 (C), 143.4 (C), 136.8 (C), 133.0 (C), 130.5 (CH), 128.9 (2 CH), 61.6 (CH_2), 52.5 (CH_3), 49.3 (CH), 41.0 (CH_2), 28.4 (CH_2), 20.5 (CH_2), 14.2 (CH_3) ppm. IR (ATR): λ^{-1} = 2953 (m), 2872 (w), 1722 (s), 1684 (s),

1609 (w), 1569 (w), 1437 (m), 1409 (w), 1370 (w), 1286 (s), 1244 (s), 1189 (s), 1109 (s), 1066 (m), 1024 (m), 998 (m), 920 (w), 910 (w), 886 (w), 857 (m), 770 (m), 761 (m), 707 (w), 659 (w) cm^{-1}. MS (EI, 70 eV): m/z (%) 290 (15) [M^{+}], 244 (30), 233 (28), 217 (35), 216 (25), 189 (52), 157 (35), 155 (20), 131 (25), 130 (60), 128 (97), 115 (97), 102 (89), 91 (49), 89 (30), 77 (50), 76 (37), 63 (27), 59 (100), 49 (82). HRMS (EI, 70 eV): calcd. 290.1149 (for $C_{16}H_{18}O_5^{+}$), found 290.1141 [M^{+}].

3. α-(*ortho*-Iodophenylation) of β-oxo esters 1

3.1 General procedure B (GPB) for the preparation of α-arylated β-oxo esters 5

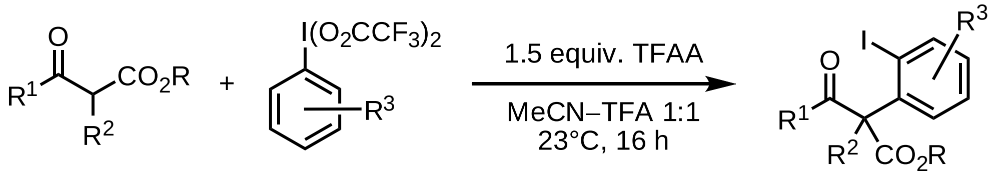

1a–**1g** **9a**, **9i**–**9m** (1.3 equiv.) **5a**–**5m**

Following the protocol published by Jia *et al.*,[S5] anhydrous MeCN (2.5 L mol^{-1}) and TFA (2.5 L mol^{-1}) were added under nitrogen atmosphere and at ambient temperature to the PIFA derivative **9** (1.3 equiv.). Trifluoroacetic anhydride (TFAA, 1.5 equiv.) was then added and the resulting solution was stirred for 15 min. Subsequently, the β-oxo ester **1** (1.0 equiv.) was added and the resulting mixture was stirred at ambient temperature for further 18 h. All volatiles were removed *in vacuo* and the crude product was purified by column chromatography to give the α-arylated β-oxo esters **5**.

3.2 Ethyl 1-(2-iodophenyl)-2-oxocyclopentane-1-carboxylate (5a)

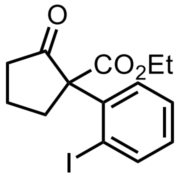

According to GPB, PIFA (**9a**) (2.80 g, 6.50 mmol), ethyl 2-oxocyclopentane-1-carboxylate (**1a**) (0.74 mL, 0.78 g, 5.0 mmol) and TFAA (1.0 mL, 1.6 g, 7.5 mmol) were converted to furnish compound **5a** (935 mg, 2.61 mmol, 52%) after column chromatography (SiO_2, hexanes/MTBE 3:1, R_f = 0.34) as a colorless oil. ^{1}H NMR (300 MHz, $CDCl_3$): δ = 7.93 (dd, J = 7.7 Hz, J = 1.1 Hz, 1 H), 7.28 (td, J = 7.6 Hz, J = 1.2 Hz, 1 H), 6.97–6.92 (m, 2 H), 4.27–4.16 (m, 2 H), 3.20 (ddd, J = 13.5 Hz, J = 9.8 Hz, J = 7.0 Hz, 1 H), 2.58–2.48 (m, 3 H), 2.14–2.03 (m, 1 H), 1.76–1.66 (m, 1 H), 1.24 (t, J = 7.1 Hz, 3 H) ppm. The spectroscopic data are in accordance with literature values.[S5]

3.3 Ethyl 1-(2-iodophenyl)-2-oxocyclohexane-1-carboxylate (5b)

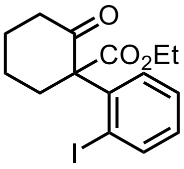

According to GPB, PIFA (**9a**) (1.74 g, 4.04 mmol), ethyl 2-oxocyclohexane-1-carboxylate (**1b**) (0.50 mL, 0.53 g, 3.1 mmol) and TFAA (0.65 mL, 0.98 g, 4.7 mmol) were converted to furnish compound **5b** (463 mg, 1.24 mmol, 40%) after column chromatography (SiO_2, hexanes/MTBE 3:1, R_f = 0.30) as a colorless oil. ^{1}H NMR (300 MHz, $CDCl_3$): δ = 7.96 (dd, J = 7.7 Hz, J = 1.2 Hz, 1 H), 7.32 (td, J = 7.7 Hz, J = 1.2 Hz, 1 H), 7.06 (dd, J = 7.7 Hz, J = 1.5 Hz, 1 H), 6.96 (td, J = 7.7 Hz, J = 1.5 Hz, 1 H), 4.34–4.22 (m, 2 H), 2.85–2.57 (m, 4 H), 2.11–1.97 (m, 2 H), 1.89–1.76 (m, 2 H), 1.27 (t, J = 7.1 Hz, 3 H) ppm. The spectroscopic data are in accordance with literature values.[S5]

3.4 Methyl 1-(2-iodophenyl)-2-oxocycloheptane-1-carboxylate (5c)

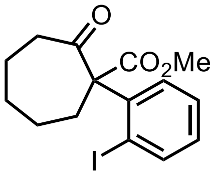

According to GPB, PIFA (**9a**) (1.81 g, 4.20 mmol), methyl 2-oxocycloheptane-1-carboxylate (**1c**) (0.50 mL, 0.55 g, 3.2 mmol) and TFAA (0.68 mL, 1.0 g, 4.9 mmol) were converted to furnish compound **5c** (554 mg, 1.49 mmol, 46%) after column chromatography (SiO_2, hexanes/MTBE 3:1, R_f = 0.41) as a colorless oil. ^{1}H NMR (300 MHz, $CDCl_3$): δ = 7.94 (dd, J = 7.7 Hz, J = 1.4 Hz, 1 H), 7.31 (td, J = 7.7 Hz, J = 1.2 Hz, 1 H), 7.02 (dd, J = 7.7 Hz, J = 1.2 Hz, 1 H), 6.95 (td, J = 7.7 Hz, J = 1.4 Hz, 1 H), 3.73 (s, 3 H), 3.22–3.15 (m, 1 H), 3.03–2.96 (m, 1 H), 2.80–2.72 (m, 1 H), 2.19–2.11 (m, 1 H), 1.80–1.68 (m, 5 H), 1.56–1.47 (m, 1 H) ppm. The spectroscopic data are in accordance with literature values.[S5]

3.5 Methyl 2-oxocyclooctane-1-carboxylate (1d)

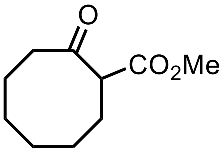

A solution of cyclooctanone (2.00 g, 15.8 mmol) in THF (15 mL) was dropwise added to a stirred suspension of NaH (1.33 g, 60% dispersion in mineral oil, 33.2 mmol) and dimethyl carbonate (447 mg, 3.18 mmol) in THF (30 mL). After heating the mixture to reflux for 4 h, hydrochloric acid (1 mol L^{-1}, 25 mL) was added. The mixture was extracted with MTBE (3 × 50 mL). The combined organic layers were dried over $MgSO_4$ and filtered. The solvent was removed *in vacuo* and the crude product was purified by column chromatography (SiO_2, hex-

anes/MTBE 10:1, R_f = 0.37) to give compound **1d** (2.35 g, 12.8 mmol, 81%) as a colorless liquid. According to ^{1}H NMR, the compound is predominantly the enol tautomer (enol/keto 88:12). ^{1}H NMR (300 MHz, $CDCl_3$), enol tautomer: δ = 12.51 (s, 1 H), 3.75 (s, 3 H), 2.42–2.32 (m, 4 H), 1.76–1.68 (m, 2 H), 1.57–1.42 (m, 6 H) ppm; the signals of the keto tautomer could not be assigned with certainty due to overlapping with the enol tautomer. The spectroscopic data are in accordance with literature values.[S6]

3.6 Methyl 1-(2-iodophenyl)-2-oxocyclooctane-1-carboxylate (5d)

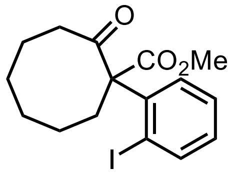

According to GPB, PIFA (**9a**) (3.04 g, 7.06 mmol), methyl 2-oxocyclooctane-1-carboxylate (**1d**) (1.00 g, 5.43 mmol) and TFAA (1.1 mL, 1.7 g, 8.2 mmol) were converted to furnish compound **5d** (1.56 g, 4.04 mmol, 74%) after column chromatography (SiO_2, hexanes/MTBE 5:1, R_f = 0.32) as a colorless oil. ^{1}H NMR (300 MHz, $CDCl_3$): δ = 7.94 (dd, *J* = 7.8 Hz, *J* = 1.2 Hz, 1 H), 7.44 (dd, *J* = 8.0 Hz, *J* = 1.7 Hz, 1 H), 7.36 (td, *J* = 8.1 Hz, *J* = 1.4 Hz, 1 H), 6.96 (td, *J* = 7.7 Hz, *J* = 1.8 Hz, 1 H), 3.70 (s, 3 H), 3.13 (ddd, *J* = 13.1 Hz, *J* = 9.4 Hz, *J* = 4.0 Hz, 1 H), 2.78 (ddd, *J* = 15.1 Hz, *J* = 8.9 Hz, *J* = 3.6 Hz, 1 H), 2.66 (ddd, *J* = 15.1 Hz, *J* = 8.1 Hz, *J* = 3.0 Hz, 1 H), 2.49 (ddd, *J* = 12.4 Hz, *J* = 8.1 Hz, *J* = 4.0 Hz, 1 H), 1.94–1.78 (m, 3 H), 1.65–1.49 (m, 3 H), 1.42–1.32 (m, 2 H) ppm. $^{13}C\{^1H\}$ NMR (75 MHz, $CDCl_3$): δ = 209.5 (C), 171.1 (C), 142.3 (CH), 140.4 (C), 130.1 (CH), 129.0 (CH), 127.9 (CH), 100.0 (C), 71.4 (C), 53.3 (CH_3), 39.8 (CH_2), 33.2 (CH_2), 28.9 (CH_2), 26.5 (CH_2), 25.9 (CH_2), 24.6 (CH_2) ppm. IR (ATR): λ^{-1} = 2927 (m), 2856 (w), 1729 (vs), 1706 (vs), 1216 (vs) cm^{-1}. MS (EI, 70 eV): *m/z* (%) 386 (53) [M^+], 354 (6), 326 (15), 289 (23), 259 (100), 229 (71), 227 (70), 217 (59), 199 (63), 189 (30), 171 (30), 161 (87), 129 (76), 115 (81), 103 (71), 91 (60), 55 (57). HRMS (EI, 70 eV): calcd. 386.0373 (for $C_{16}H_{19}IO_3^+$), found 386.0374 [M^+].

3.7 Methyl 2-(2-iodophenyl)-1-oxoindane-2-carboxylate (5e)

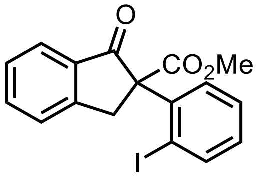

According to GPB, PIFA (**9a**) (851 mg, 1.98 mmol), methyl 1-oxoindane-2-carboxylate (**1e**) (290 mg, 1.52 mmol) and TFAA (0.32 mL, 0.48 g, 2.3 mmol) were converted to furnish compound **5e** (301 mg, 0.77 mmol, 51%) after column chromatography (SiO_2, hexanes/MTBE 3:1, R_f = 0.41) as a colorless oil. ^{1}H NMR (300 MHz, $CDCl_3$): δ = 8.03 (dd, *J* = 7.9 Hz, *J* = 1.2 Hz, 1 H), 7.97 (d, *J* = 7.7 Hz, 1 H), 7.74 (dt, *J* = 7.6 Hz, *J* = 1.0 Hz, 1 H), 7.57–7.49 (m, 2 H),

7.34 (td, J = 7.6 Hz, J = 1.3 Hz, 1 H), 7.25 (dd, J = 7.9 Hz, J = 1.7 Hz, 1 H), 7.04 (td, J = 7.6 Hz, J = 1.7 Hz, 1 H), 4.75 (d, J = 17.5 Hz, 1 H), 3.86 (s, 3 H), 3.42 (d, J = 17.5 Hz, 1 H) ppm. The spectroscopic data are in accordance with literature values.[S5]

3.8 Ethyl 2-(2-iodophenyl)-1-oxo-1,2,3,4-tetrahydronaphthalene-2-carboxylate (5f)

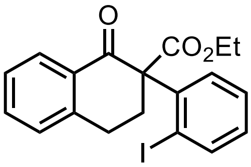

According to GPB, PIFA (**9a**) (1.28 g, 2.98 mmol), ethyl 1-oxo-1,2,3,4-tetrahydronaphthalene-2-carboxylate (**1f**) (500 mg, 2.29 mmol) and TFAA (0.48 mL, 0.72 g, 3.4 mmol) were converted to furnish compound **5f** (179 mg, 0.426 mmol, 19%) after column chromatography (SiO_2, hexanes/MTBE 3:1, R_f = 0.36) as a colorless oil. ^{1}H NMR (300 MHz, $CDCl_3$): δ = 8.17 (dd, J = 7.8 Hz, J = 0.8 Hz, 1 H), 7.96 (dd, J = 7.8 Hz, J = 1.1 Hz, 1 H), 7.50 (td, J = 7.6 Hz, J = 1.2 Hz, 1 H), 7.37 (t, J = 7.5 Hz, 1 H), 7.17 (d, J = 8.0 Hz, 1 H), 7.12 (td, J = 7.7 Hz, J = 1.2 Hz, 1 H), 6.92 (td, J = 7.7 Hz, J = 1.4 Hz, 1 H), 6.84 (dd, J = 7.9 Hz, J = 1.4 Hz, 1 H), 4.36 (dq, J = 10.7 Hz, J = 7.1 Hz, 1 H), 4.27 (dq, J = 10.7 Hz, J = 7.1 Hz, 1 H), 3.49–3.41 (m, 1 H), 2.90–2.77 (m, 2 H), 2.52–2.41 (m, 1 H), 1.29 (t, J = 7.1 Hz, 3 H) ppm. ^{13}C{^{1}H} NMR (75 MHz, $CDCl_3$): δ = 194.9 (C), 170.7 (C), 143.6 (C), 142.6 (CH), 139.4 (C), 134.0 (CH), 132.7 (C), 130.1 (CH), 129.1 (CH), 128.9 (CH), 128.3 (CH), 128.0 (CH), 127.2 (CH), 97.6 (C), 67.0 (C), 62.3 (CH_2), 31.2 (CH_2), 25.8 (CH_2), 14.1 (CH_3) ppm. IR (ATR): λ^{-1} = 2993 (w), 1729 (s), 1681 (s), 1232 (s), 1009 (s), 739 (vs) cm^{-1}. HRMS (ESI): calcd. 443.0115 (for $C_{19}H_{17}INaO_3^+$), found 443.0110 [M + Na$^+$].

3.9 Ethyl 2-(2-iodophenyl)-2-methyl-3-oxobutanoate (5g)

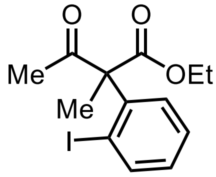

According to GPB, PIFA (**9a**) (1.94 g, 4.51 mmol), ethyl 2-methyl-3-oxobutanoate **1g** (500 mg, 3.47 mmol) and TFAA (0.72 mL, 1.1 g, 5.2 mmol) were converted to furnish compound **5g** (533 mg, 1.54 mmol, 44%) after column chromatography (SiO_2, hexanes/MTBE 5:1, R_f = 0.25) as a colorless oil. ^{1}H NMR (300 MHz, $CDCl_3$): δ = 7.94 (t, J = 7.7 Hz, 1 H), 7.36 (t, J = 7.7 Hz, 1 H), 7.11 (d, J = 7.7 Hz, 1 H), 6.98 (t, J = 7.7 Hz, 1 H), 4.32–4.21 (m, 2 H), 2.44 (s, 3 H), 1.86 (s, 3 H), 1.28 (t, J = 7.2 Hz, 3 H) ppm. ^{13}C{^{1}H} NMR (75 MHz, $CDCl_3$): δ = 205.2 (C), 171.3 (C), 142.9 (C), 142.2 (CH), 129.08 (CH), 129.02 (CH), 128.5 (CH), 98.7 (C), 68.6 (C), 62.4 (CH_2), 29.4 (CH_3), 22.2 (CH_3), 14.0 (CH_3) ppm. IR (ATR): λ^{-1} = 2984 (w), 1712 (vs), 1232 (s), 1167 (s), 1103 (s), 1010 (s), 754 (s) cm^{-1}. MS (EI, 70 eV): m/z (%) 346 (<1) [M$^+$],

304 (50), 258 (5), 219 (7), 177 (31), 149 (24), 148 (20), 146 (16), 131 (23), 121 (12), 103 (52), 91 (10), 77 (24), 41 (9). HRMS (EI, 70 eV): calcd. 346.0060 (for $C_{13}H_{15}IO_3^+$), found 346.0069 [M^+].

3.10 Ethyl 2-iodophenylacetate

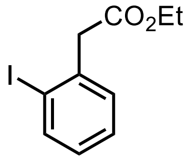

Conc. sulfuric acid (0.13 g, 1.4 mmol) was added to a stirred solution of 2-iodoacetic acid (2.00 g, 7.63 mmol) in EtOH (30 mL). After heating the mixture to reflux for 4 h, water (60 mL) was added. The mixture was extracted with MTBE (3 × 50 mL). The combined organic layers were washed with $NaHCO_3$ (saturated aqueous solution, 30 mL), dried over $MgSO_4$ and filtered. The solvent was removed *in vacuo* to give ethyl 2-iodophenylacetate (1.74 g, 6.00 mmol, 79%) as a yellow liquid. ^{1}H NMR (300 MHz, $CDCl_3$): δ = 7.85 (d, J = 8.0 Hz, 1 H), 7.35–7.27 (m, 2 H), 6.99–6.93 (m, 1 H), 4.19 (q, J = 7.1 Hz, 2 H), 3.79 (s, 2 H), 1.28 (t, J = 7.1 Hz, 3 H) ppm. The spectroscopic data are in accordance with literature values.[S7]

3.11 Ethyl 2-(2-iodophenyl)-3-phenyl-3-oxopropionate (5h)

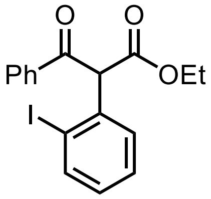

Under nitrogen atmosphere, 1-methylimidazole (0.30 mL, 0.31 g, 3.8 mmol) was added drop-wise to a stirred solution of ethyl 2-iodophenylacetate (923 mg, 3.18 mmol) and benzoyl chloride (447 mg, 3.18 mmol) in anhydrous CH_2Cl_2 (10 mL) at –45 °C. At this temperature, $TiCl_4$ (1.2 mL, 2.1 g, 11 mmol) and NEt_3 (1.8 mL, 1.3 g, 13 mmol) were dropwise added. After stirring the mixture at this temperature for 1 h, water (20 mL) was added. The mixture was extracted with MTBE (3 × 20 mL). The combined organic layers were dried over $MgSO_4$ and filtered. The solvent was removed *in vacuo* and the crude product was purified by column chromatography (SiO_2, hexanes/MTBE 3:1, R_f = 0.54) to give compound **5h** (1.16 g, 2.94 mmol, 92%) as a yellow liquid. The NMR spectra showed a doubled signal set due to keto/enol tautomers (ratio 20:80). ^{1}H NMR (300 MHz, $CDCl_3$), enol tautomer: δ = 13.50 (s, 1 H), 7.74 (dd, J = 7.9 Hz, J = 1.0 Hz, 1 H), 7.18–7.11 (m, 3 H), 7.08–7.03 (m, 3 H), 6.93 (dd, J = 7.6 Hz, J = 1.7 Hz, 1 H), 6.80 (td, J = 7.6 Hz, J = 1.8 Hz, 1 H), 4.30–4.01 (m, 2 H), 1.19–1.14 (m, 3 H) ppm; keto tautomer: δ = 7.85–7.82 (m, 2 H), 7.81–7.78 (m, 1 H), 7.36–7.29 (m, 2 H), 7.25–7.21 (m, 1 H), 7.18–7.11 (m, 2 H), 6.90–6.84 (m, 1 H), 5.94 (s, 1 H), 4.30–4.01 (m, 2 H), 1.19–1.14 (m, 3 H) ppm. ^{13}C{^{1}H} NMR (75 MHz, $CDCl_3$), enol tautomer: δ = 172.4

(C), 171.0 (C), 140.3 (C), 138.9 (CH), 134.5 (C), 132.8 (CH), 129.9 (CH), 128.8 (CH), 128.6 (2 CH), 128.2 (CH), 127.8 (2 CH), 108.1 (C), 105.3 (C), 61.3 (CH_2), 14.3 (CH_3) ppm; keto tautomer: δ = 193.7 (C), 168.3 (C), 139.9 (CH), 136.7 (C), 135.5 (C), 133.8 (CH), 130.5 (CH), 130.2 (CH), 129.9 (CH), 129.0 (2 CH), 128.9 (2 CH), 101.8 (C), 65.2 (CH), 62.0 (CH_2), 14.2 (CH_3) ppm. IR (ATR): λ^{-1} = 3059 (w), 2987 (w), 2934 (w), 1714 (vs), 1334 (s), 1250 (vs), 1134 (s), 744 (s), 696 (s) cm^{-1}. MS (EI, 70 eV): *m/z* (%) 394 (48) [M^+], 267 (31), 244 (30), 221 (86), 194 (12), 177 (10), 165 (56), 105 (100), 89 (23), 77 (83), 51 (19). HRMS (EI, 70 eV): calcd. 394.0060 (for $C_{17}H_{15}IO_3^+$), found 394.0053 [M^+].

3.12 Ethyl 1-(2-iodo-3-methylphenyl)-2-oxocyclopentane-1-carboxylate (5i)

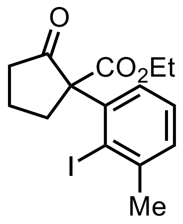

According to GPB, 2-methylphenyliodine bis(trifluoroacetate) (**9i**) (2.65 g, 5.97 mmol), ethyl 2-oxocyclopentane-1-carboxylate (**1a**) (0.68 mL, 0.72 g, 4.6 mmol) and TFAA (0.96 mL, 1.5 g, 6.9 mmol) were converted to furnish compound **5i** (866 mg, 2.33 mmol, 51%) after column chromatography (SiO_2, hexanes/MTBE 5:1, R_f = 0.26) as a light yellow oil. ^{1}H NMR (500 MHz, DMSO-d_6): δ = 7.28 (dd, J = 7.5 Hz, J = 1.5 Hz, 1 H), 7.24 (t, J = 7.5 Hz, 1 H), 6.73 (dd, J = 7.5 Hz, J = 1.5 Hz, 1 H), 4.15 (dq, J = 10.9 Hz, J = 7.1 Hz, 1 H), 4.07 (dq, J = 10.9 Hz, J = 7.1 Hz, 1 H), 3.04 (ddd, J = 13.3 Hz, J = 10.6 Hz, J = 6.9 Hz, 1 H), 2.69 (dddd, J = 19.1 Hz, J = 8.5 Hz, J = 3.8 Hz, J = 1.8 Hz, 1 H), 2.52–2.47 (m, 2 H), 2.45 (s, 3 H), 2.03–1.96 (m, 1 H), 1.58–1.50 (m, 1 H), 1.15 (t, J = 7.1 Hz, 3 H) ppm. ^{13}C{^{1}H} NMR (125 MHz, DMSO-d_6): δ = 213.7 (C), 169.2 (C), 143.4 (C), 141.9 (C), 128.6 (CH), 127.7 (CH), 126.3 (CH), 106.7 (C), 70.5 (C), 61.4 (CH_2), 38.8 (CH_2), 35.9 (CH_2), 30.2 (CH_3), 18.8 (CH_2), 13.8 (CH_3) ppm. IR (ATR): λ^{-1} = 2977 (w), 1749 (m), 1717 (vs), 1220 (s), 1004 (s) cm^{-1}. MS (EI, 70 eV): *m/z* (%) 372 (66) [M^+], 315 (25), 299 (44), 284 (40), 271 (66), 245 (100), 243 (83), 217 (85), 199 (58), 189 (80), 172 (81), 162 (80), 143 (78), 130 (82), 128 (80), 105 (24), 91 (33), 77 (25), 55 (59). HRMS (EI, 70 eV): calcd. 372.0217 (for $C_{15}H_{17}IO_3^+$), found 372.0206 [M^+].

3.13 Ethyl 1-(3-fluoro-2-iodophenyl)-2-oxocyclopentane-1-carboxylate (5j)

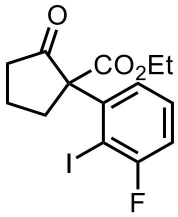

According to GPB, 2-fluorophenyliodine bis(trifluoroacetate) (**9j**) (2.39 g, 5.33 mmol), ethyl 2-oxocyclopentane-1-carboxylate (**1a**) (0.61 mL, 0.64 g, 4.1 mmol) and TFAA (0.85 mL, 1.3 g, 6.2 mmol) were converted to furnish compound **5j** (421 mg, 1.12 mmol, 27%) after column chromatography (SiO_2, hexanes/MTBE 5:1, R_f = 0.21) as a colorless oil. ^{1}H NMR (500 MHz, $CDCl_3$): δ = 7.26–7.22 (m, 1 H), 7.00–6.97 (m, 1 H), 6.76–6.74 (m, 1 H), 4.24 (dq, J = 10.7 Hz, J = 7.1 Hz, 1 H), 4.18 (dq, J = 10.7 Hz, J = 7.1 Hz, 1 H), 3.22 (ddd, J = 13.7 Hz, J = 9.6 Hz, J = 7.0 Hz, 1 H), 2.57–2.47 (m, 3 H), 2.14–2.07 (m, 1 H), 1.76–1.68 (m, 1 H), 1.22 (t, J = 7.1 Hz, 3 H) ppm. ^{13}C{^{1}H} NMR (125 MHz, $CDCl_3$): δ = 213.4 (C), 169.5 (C), 162.4 (d, J = 244.4 Hz, C), 144.2 (C), 129.9 (d, J = 8.6 Hz, CH), 124.4 (d, J = 2.7 Hz, CH), 114.5 (d, J = 25.6 Hz, CH), 87.2 (d, J = 26.2 Hz, C), 69.9 (d, J = 1.6 Hz, C), 62.6 (CH_2), 39.6 (CH_2), 36.3 (CH_2), 19.5 (CH_2), 14.0 (CH_3) ppm. ^{19}F{^{1}H} NMR (470 MHz, $CDCl_3$): δ = –84.60 ppm. IR (ATR): λ^{-1} = 2980 (w), 1752 (s), 1710 (vs), 1223 (vs), 527 (s) cm^{-1}. MS (EI, 70 eV): *m/z* (%) 376 (40) [M^+], 348 (10), 250 (94), 222 (20), 193 (51), 177 (40), 162 (23), 148 (75), 135 (97), 121 (100), 109 (30), 101 (36), 75 (8), 55 (23). HRMS (EI, 70 eV): calcd. 375.9966 (for $C_{14}H_{14}FIO_3^+$), found 375.9966 [M^+].

3.14 Ethyl 1-(5-bromo-2-iodophenyl)-2-oxocyclopentane-1-carboxylate (5k)

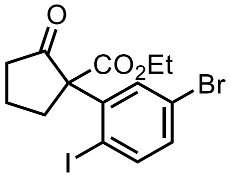

According to GPB, 4-bromophenyliodine bis(trifluoroacetate) (**9k**) (1.81 g, 3.56 mmol), ethyl 2-oxocyclopentane-1-carboxylate (**1a**) (0.41 mL, 0.43 g, 2.7 mmol) and TFAA (0.57 mL, 0.86 g, 4.1 mmol) were converted to furnish compound **5k** (534 mg, 1.22 mmol, 45%) after column chromatography (SiO_2, hexanes/MTBE 5:1, R_f = 0.32) as a colorless oil. ^{1}H NMR (300 MHz, $CDCl_3$): δ = 7.76–7.73 (m, 1 H), 7.09–7.06 (m, 2 H), 4.27–4.12 (m, 2 H), 3.21 (ddd, J = 13.6 Hz, J = 9.0 Hz, J = 7.1 Hz, 1 H), 2.57–2.51 (m, 2 H), 2.47–2.39 (m, 1 H), 2.17–2.04 (m, 1 H), 1.81–1.66 (m, 1 H), 1.22 (t, J = 7.2 Hz, 3 H) ppm. ^{13}C{^{1}H} NMR (75 MHz, $CDCl_3$): δ = 212.9 (C), 168.9 (C), 143.8 (C), 143.1 (CH), 132.0 (CH), 131.8 (CH), 122.6 (C), 96.8 (C), 69.6 (C), 62.6 (CH_2), 39.5 (CH_2), 36.1 (CH_2), 19.4 (CH_2), 14.0 (CH_3) ppm. IR (ATR): λ^{-1} = 2979 (w), 1718 (s), 1227 (s), 1008 (s) cm^{-1}. MS (EI, 70 eV): *m/z* (%) 436 (13) [M^+], 408 (12), 381 (22), 353 (21), 337 (24), 309 (100), 281 (42), 253 (44), 235 (38), 226 (46), 211 (27), 186

(29), 180 (31), 128 (68), 115 (60), 101 (44), 75 (38), 63 (18), 55 (47). HRMS (EI, 70 eV): calcd. 435.9166 (for $C_{14}H_{14}BrIO_3^+$), found 435.9157 [M^+].

3.15 Ethyl 1-(2-iodo-5-trifluoromethylphenyl)-2-oxocyclopentane-1-carboxylate (5l)

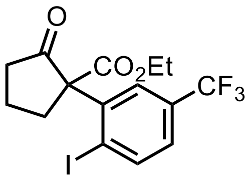

According to GPB, 4-trifluoromethylphenyliodine bis(trifluoroacetate) (**9l**) (1.56 g, 3.13 mmol), ethyl 2-oxocyclopentane-1-carboxylate (**1a**) (0.36 mL, 0.38 g, 2.4 mmol) and TFAA (0.50 mL, 0.76 g, 3.6 mmol) were converted to furnish compound **5l** (404 mg, 0.948 mmol, 39%) after column chromatography (SiO_2, hexanes/MTBE 5:1, R_f = 0.32) as a colorless oil. ^{1}H NMR (500 MHz, $CDCl_3$): δ = 8.06–8.05 (m, 1 H), 7.20–7.18 (m, 2 H), 4.23–4.17 (m, 2 H), 3.25 (ddd, J = 13.6 Hz, J = 8.5 Hz, J = 7.1 Hz, 1 H), 2.58–2.54 (m, 2 H), 2.47–2.42 (m, 1 H), 2.17–2.09 (m, 1 H), 1.79–1.70 (m, 1 H), 1.22 (t, J = 7.1 Hz, 3 H) ppm. ^{13}C{^{1}H} NMR (125 MHz, $CDCl_3$): δ = 212.5 (C), 168.9 (C), 143.0 (C), 142.6 (CH), 130.6 (q, J = 32.8 Hz, C), 125.5 (q, J = 3.6 Hz, CH), 125.1 (q, J = 3.6 Hz, CH), 123.7 (q, J = 273.3 Hz, CF_3), 103.3 (C), 69.7 (C), 62.6 (CH_2), 39.5 (CH_2), 36.2 (CH_2), 19.4 (CH_2), 13.9 (CH_3) ppm. ^{19}F{^{1}H} NMR (470 MHz, $CDCl_3$): δ = –62.90 (CF_3) ppm. IR (ATR): λ^{-1} = 2982 (w), 1749 (w), 1717 (vs), 1713 (vs), 1224 (s), 1126 (vs), 1093 (s), 1083 (s), 1011 (s) cm^{-1}. HRMS (LIFDI): calcd. 425.9934 (for $C_{15}H_{14}F_3IO_3^+$), found 425.9934 [M^+].

3.16 Ethyl 1-(2-iodo-5-methoxycarbonylphenyl)-2-oxocyclopentane-1-carboxylate (5m)

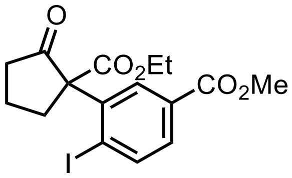

According to GPB, 4-methoxycarbonylphenyliodine bis(trifluoroacetate) (**9m**) (0.45 g, 0.91 mmol), ethyl 2-oxocyclopentane-1-carboxylate (**1a**) (0.11 g, 0.70 mmol) and TFAA (0.22 g, 1.1 mmol) were converted to furnish compound **5m** (237 mg, 0.570 mmol, 81%) after column chromatography (SiO_2, hexanes/MTBE 5:1, R_f = 0.14) as a colorless oil. ^{1}H NMR (300 MHz, $CDCl_3$): δ = 8.01–7.98 (m, 1 H), 7.57–7.54 (m, 2 H), 4.23–4.10 (m, 2 H), 3.83 (s, 3 H), 3.19 (ddd, J = 13.5 Hz, J = 9.2 Hz, J = 7.1 Hz, 1 H), 2.66–2.40 (m, 3 H), 2.16–2.05 (m, 1 H), 1.79–1.63 (m, 1 H), 1.19 (t, J = 7.1 Hz, 3 H) ppm. ^{13}C{^{1}H} NMR (75 MHz, $CDCl_3$): δ = 212.8 (C), 169.1 (C), 166.1 (C), 142.22 (CH), 142.21 (C), 130.1 (C), 129.5 (CH), 129.2 (CH), 105.1 (C), 69.7 (C), 62.5 (CH_2), 52.4 (CH_3), 39.4 (CH_2), 36.0 (CH_2), 19.4 (CH_2), 13.9 (CH_3) ppm. IR (ATR): λ^{-1} = 2982 (w), 2954 (w), 2900 (w), 1741 (m), 1710 (vs), 1257 (s), 1223 (s), 1084 (s), 754 (s) cm^{-1}. MS (EI, 70 eV): *m/z* (%) 416 (80) [M^+], 388 (23), 359 (20), 329 (61), 314 (46),

301 (75), 289 (100), 282 (70), 261 (39), 257 (20), 233 (28), 215 (34), 205 (37), 191 (28), 174 (38), 128 (78), 115 (51), 101 (23), 55 (33). HRMS (EI, 70 eV): calcd. 416.0115 (for $C_{16}H_{17}IO_5^+$), found 416.0110 [M^+].

4. Synthesis of PIFA derivatives 9

4.1 General procedure C (GPC) for the synthesis of PIFA derivatives 9

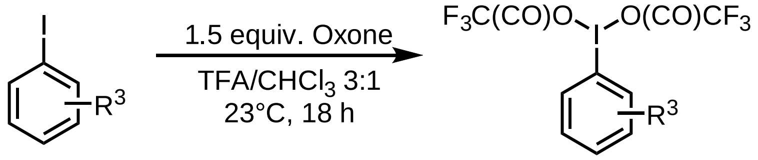

Following the procedure published by Zagulyaeva *et al.*,[S8] oxone (1.5 equiv.) was added to a solution of the iodobenzene derivative (1.0 equiv.) in TFA (1.5 L mol^{-1}) and $CHCl_3$ (0.5 L mol^{-1}). The resulting mixture was stirred for 18 h at 23 °C. All volatiles were removed *in vacuo* and the crude product was extracted with $CHCl_3$ (15 L mol^{-1}). The solvent was evaporated to give the PIFA derivatives **9i–9m**.

4.2 2-Methylphenyliodine bis(trifluoroacetate) (9i)

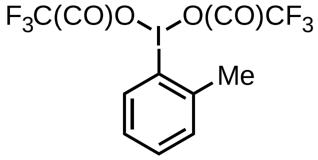

According to GPC, oxone (4.24 g, 13.8 mmol) and *ortho*-iodotoluene (2.00 g, 9.17 mmol) were converted to furnish compound **9i** (2.65 g, 5.97 mmol, 65%) as a light yellow solid (mp 95–96 °C). ^{1}H NMR (300 MHz, $CDCl_3$): δ = 8.27 (d, J = 8.2 Hz, 1 H), 7.68–7.60 (m, 2 H), 7.35 (td, J = 8.4 Hz, J = 2.2 Hz, 1 H), 2.79 (s, 3 H) ppm. The spectroscopic data are in accordance with literature values.[S5]

4.3 2-Fluorophenyliodine bis(trifluoroacetate) (9j)

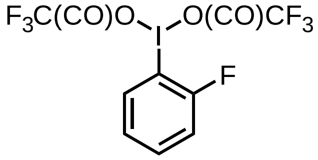

According to GPC, oxone (4.15 g, 13.5 mmol) and 2-fluoroiodobenzene (2.00 g, 9.01 mmol) were converted to furnish compound **9j** (2.51 g, 5.60 mmol, 62%) as a colorless solid (mp 129–132 °C). ^{1}H NMR (500 MHz, $CDCl_3$): δ = 8.27 (ddd, J = 8.1 Hz, J = 5.6 Hz, J = 1.5 Hz, 1 H), 7.79–7.74 (m, 1 H), 7.51 (td, J = 8.3 Hz, J = 1.4 Hz, 1 H), 7.38 (td, J = 7.7 Hz, J = 1.4 Hz, 1 H) ppm. ^{13}C{^{1}H} NMR (125 MHz, $CDCl_3$): δ = 161.5 (q, J = 41.6 Hz, C), 159.2 (d, J = 257.7 Hz, C), 137.2 (d, J = 8.1 Hz, CH), 137.1 (CH), 127.4 (d, J = 3.2 Hz, CH), 117.4 (d, J = 21.6

Hz, CH), 113.1 (q, J = 287.6 Hz, CF_3), 110.2 (d, J = 22.4 Hz, C) ppm. $^{19}F\{^1H\}$ NMR (470 MHz, $CDCl_3$): δ = –73.45 (2 CF_3), –94.12 (CF) ppm. IR (ATR): λ^{-1} = 3096 (w), 1704 (s), 1663 (s), 1136 (vs), 1114 (vs) cm^{-1}. MS (EI, 70 eV): *m/z* (%) 335 (45) $[M–F_3CCO_2]^+$, 253 (35), 222 (100), 208 (15), 127 (10), 111 (15), 95 (98), 75 (80), 69 (71), 50 (24), 45 (29).

4.4 4-Bromophenyliodine bis(trifluoroacetate) (9k)

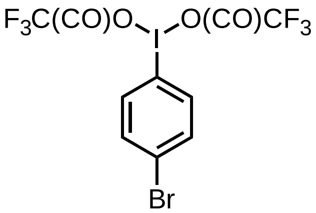

According to GPC, oxone (3.26 g, 10.6 mmol) and 4-bromoiodobenzene (2.00 g, 7.07 mmol) were converted to furnish compound **9k** (1.81 g, 3.56 mmol, 50%) as a colorless solid (mp 122–123 °C). ^1H NMR (300 MHz, $CDCl_3$): δ = 8.07–8.04 (m, 2 H), 7.76–7.73 (m, 2 H) ppm. The spectroscopic data are in accordance with literature values.[S8]

4.5 4-Trifluoromethylphenyliodine bis(trifluoroacetate) (9l)

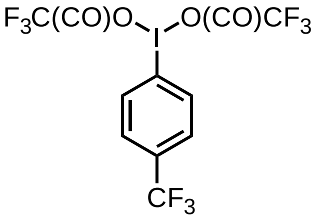

According to GPC, oxone (3.38 g, 11.0 mmol) and 4-trifluoromethyliodobenzene (2.00 g, 7.35 mmol) were converted to furnish compound **9l** (1.56 g, 3.13 mmol, 43%) as a colorless solid (mp 120–122 °C). ^1H NMR (300 MHz, $CDCl_3$ + 1 vol% TFA): δ = 8.36–8.33 (m, 2 H), 7.89–7.87 (m, 2 H) ppm. The spectroscopic data are in accordance with literature values.[S8]

4.6 4-Methoxycarbonylphenyliodine bis(trifluoroacetate) (9m)

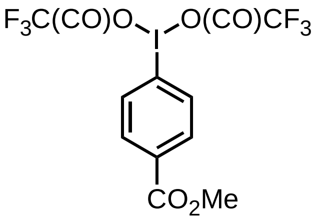

According to GPC, oxone (1.23 g, 4.01 mmol) and methyl 4-iodobenzoate (700 mg, 2.67 mmol) were converted to furnish compound **9m** (445 mg, 0.911 mmol, 39%) as a colorless solid (mp 127–129 °C). ^1H NMR (300 MHz, $CDCl_3$): δ = 8.25 (s, 4 H), 3.99 (s, 3 H) ppm. The spectroscopic data are in accordance with literature values.[S5]

5. Computational Details

All quantum chemical calculations were carried out using the Gaussian16 package.[S9] The molecular structure optimizations were performed using the M06-2X functional[S10] along with the Def2-TZVP basis set.[S11] Every stationary point was identified by a subsequent frequency calculation either as a minimum (Number of imaginary frequencies NIMAG = 0) or as a transition state (NIMAG = 1). Transition states were connected to the appropriate minima by following the intrinsic reaction coordinate (IRC) using the algorithm as implemented in the Gaussian16 program package.[S12] In the case of the TS transforming **11** into **12**, the imaginary frequency was inspected. The structure of the stationary point was distorted along this vibrational mode in both directions. The resulting two structures were optimized giving **11** and **12** as minima. The solvent was modeled using the polarizable continuum model (PCM) in the self-consistent reaction field method (SCRF) for the parameters provided by the Gaussian16 package for *N*,*N*-dimethyl formamide (DMF).[S13] Table S2 summarizes the RHF energies E^{RHF}, and the absolute Gibbs energies G^{298} [T = 298.15 K, p = 0.101 MPa (1 atm)] obtained with this method for all optimized structures. Relative energies and Gibbs energies, E^{rel} and $G^{298,rel}$, are calculated using the respective quantities of compound **11** or **14** as reference points. The corresponding computed molecular structures are given in the xyz-files and are ordered by the reaction scheme numbers.

Table S2. Calculated absolute and relative SCRF energies (E, E^{rel}) and free enthalpies at 298 K (G^{298}, $G^{298,rel}$) for compounds and transition states (TS) of interest. The results for SCRF computations were obtained with DMF as solvent. E^{rel} and $G^{298,rel}$ are calculated relative to the energies of compound **11** (grey background) or **14** (white).

Compound or TS	E^{RHF} (a.u.)	G^{298} (a.u.)	NIMAG ($\tilde{\nu}$ / cm^{-1})	E^{rel} / kJ mol^{-1}	$G^{298,rel}$ / kJ mol^{-1}
**11**	–1138.03517	–1137.74121	0	0	0
TS(**11**→**12**)	–1138.03264	–1137.73821	1 (–56)	+7	+8
**12**	–1138.14288	–1137.84332	0	–283	–268
TS(**12**→**13**)	–1138.06166	–1137.75998	1 (–122)	–69	–49
(*E*)-**13**	–1138.14170	–1137.84434	0	–279	–272
(*Z*)-**13**	–1138.14405	–1137.84574	0	–286	–274
**14**	–805.89723	–805.66922	0	0	0
TS(**14**→**15**)	–805.86965	–805.64585	1 (–928)	+72	+61
(*E*)-**15**	–805.89414	–805.66888	0	+8	+1
(*Z*)-**15**	–805.89461	–805.66893	0	+7	+1
**2a'**	–805.92718	–805.70063	0	–79	–82

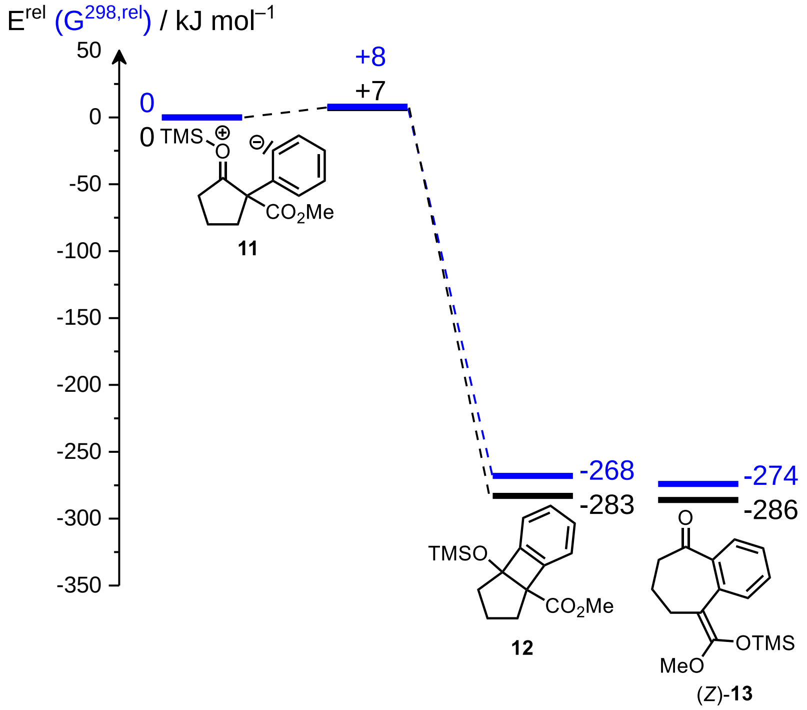

Figure S2. Calculated reaction path for the formation of the product **13** from zwitterion **11**. Relative energies E^{rel} (black) and Gibbs energies $G^{298,rel}$ (blue) are computed at the M06-2X/Def2-TZVP level with DMF as solvent and are given relative to the values of zwitterion **11**.

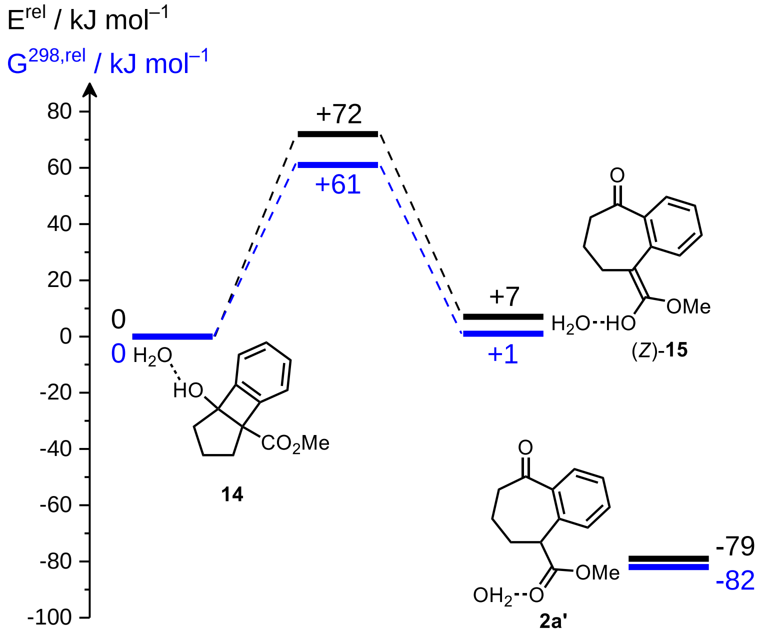

Figure S3. Calculated reaction path for the formation of the product **2a'** from compound **14**. Relative energies E^{rel} (black) and Gibbs energies $G^{298,rel}$ (blue) are computed at the M06-2X/Def2-TZVP level with DMF as solvent and are given relative to the values of compound **14**.

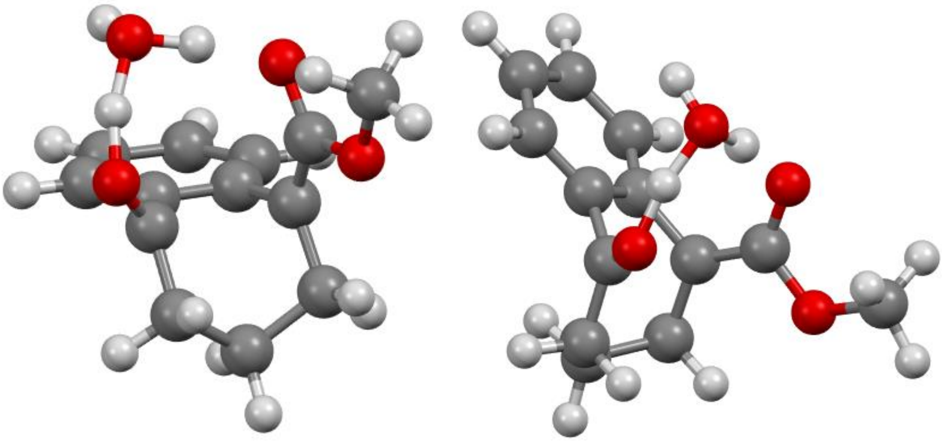

Figure S4. Calculated structure for the transition state **14**→**15**.

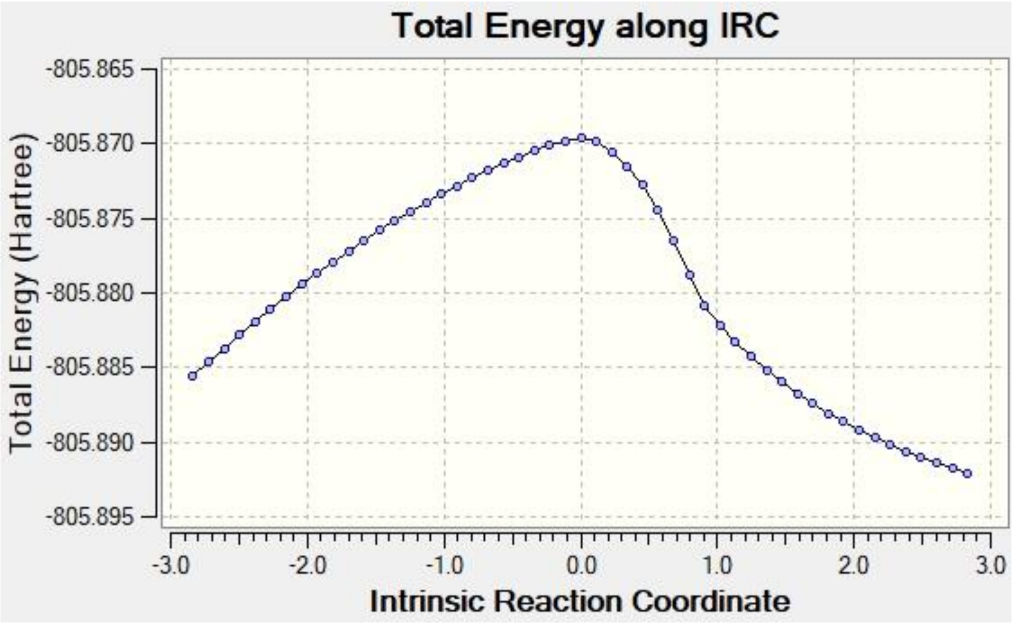

Figure S5. Graphical representation of the IRC path calculation for the formation of **15** from **14** [M06-2X/Def2-TZVP SCRF].

6. References

[S1] I. Geibel, J. Christoffers, *Eur. J. Org. Chem.* **2016**, 918–920.

[S2] U. K. Tambar, B. M. Stoltz, *J. Am. Chem. Soc.* **2005**, *127*, 5340–5341.

[S3] J. H. Kim, H. Shin, S.-g. Lee, *J. Org. Chem.* **2012**, *77*, 1560–1565.

[S4] Q. Chen, H. Yu, Z. Xu, L. Lin, X. Jiang, R. Wang, *J. Org. Chem.* **2015**, *80*, 6890–6896.

[S5] Z. Jia, E. Gálvez, R. M. Sebastián, R. Pleixats, Á. Álvarez-Larena, E. Martin, A. Vallribera, A. Shafir, *Angew. Chem. Int. Ed.* **2014**, *53*, 11298–11301; *Angew. Chem.* **2014**, *126*, 11480–11483.

[S6] F. Barabe, G. Betournay, G. Bellavance, L. Barriault, *Org. Lett.* **2009**, *11*, 4236–4238.

[S7] G. Chouhan, H. Alper, *Org. Lett.* **2008**, *10*, 4987–4990.

[S8] A. A. Zagulyaeva, M. S. Yusubov, V. V. Zhdankin, *J. Org. Chem.* **2010**, *75*, 2119–2122.

[S9] M. J. Frisch, G. W. Trucks, H. B. Schlegel, G. E. Scuseria, M. A. Robb, J. R. Cheeseman, G. Scalmani, V. Barone, B. Mennucci, G. A. Petersson, H. Nakatsuji, M. Caricato, X. Li, H. P. Hratchian, A. F. Izmaylov, J. Bloino, G. Zheng, J. L. Sonnenberg, M. Hada, M. Ehara, K. Toyota, R. Fukuda, J. Hasegawa, M. Ishida, T. Nakajima, Y. Honda, O. Kitao, H. Nakai, T. Vreven, J. A. Montgomery, Jr., J. E. Peralta, F. Ogliaro, M. Bearpark, J. J. Heyd, E. Brothers, K. N. Kudin, V. N. Staroverov, T. Keith, R. Kobayashi, J. Normand, K. Raghavachari, A. Rendell, J. C. Burant, S. S. Iyengar, J. Tomasi, M. Cossi, N. Rega, J. M. Millam, M. Klene, J. E. Knox, J. B. Cross, V. Bakken, C. Adamo, J. Jaramillo, R. Gomperts, R. E. Stratmann, O. Yazyev, A. J. Austin, R. Cammi, C. Pomelli, J. W. Ochterski, R. L. Martin, K. Morokuma, V. G. Zakrzewski, G. A. Voth, P. Salvador, J. J. Dannenberg, S. Dapprich, A. D. Daniels, O. Farkas, J. B. Foresman, J. V. Ortiz, J. Cioslowski, D. J. Fox, Gaussian 09, Revision D.01., Inc., Wallingford CT, 2013.

[S10] Y. Zhao, N. E. Schultz, D. G. Truhlar, *J. Chem. Theory Comput.* **2006**, *2*, 364–382.

[S11] a) F. Weigend, R. Ahlrichs, *Phys. Chem. Chem. Phys.* **2005**, *7*, 3297–3305; b) D. Andrae, U. Häußermann, M. Dolg, H. Stoll, H. Preuss, *Theor. Chim. Acta* **1990**, *77*, 123-141; c) B. Metz, H. Stoll, M. Dolg, *J. Chem. Phys.* **2000**,*113*, 2563–2569.

[S12] K. Fukui, *Acc. Chem. Res.* **1981**, *14*, 363–368.

[S13] J. Tomasi, B. Mennucci, R. Cammi, *Chem. Rev.* **2005**, *105*, 2999–3093.

7. NMR-Spectra of all Reported Compounds

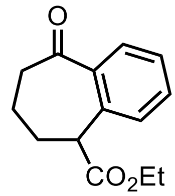

compound **2a**

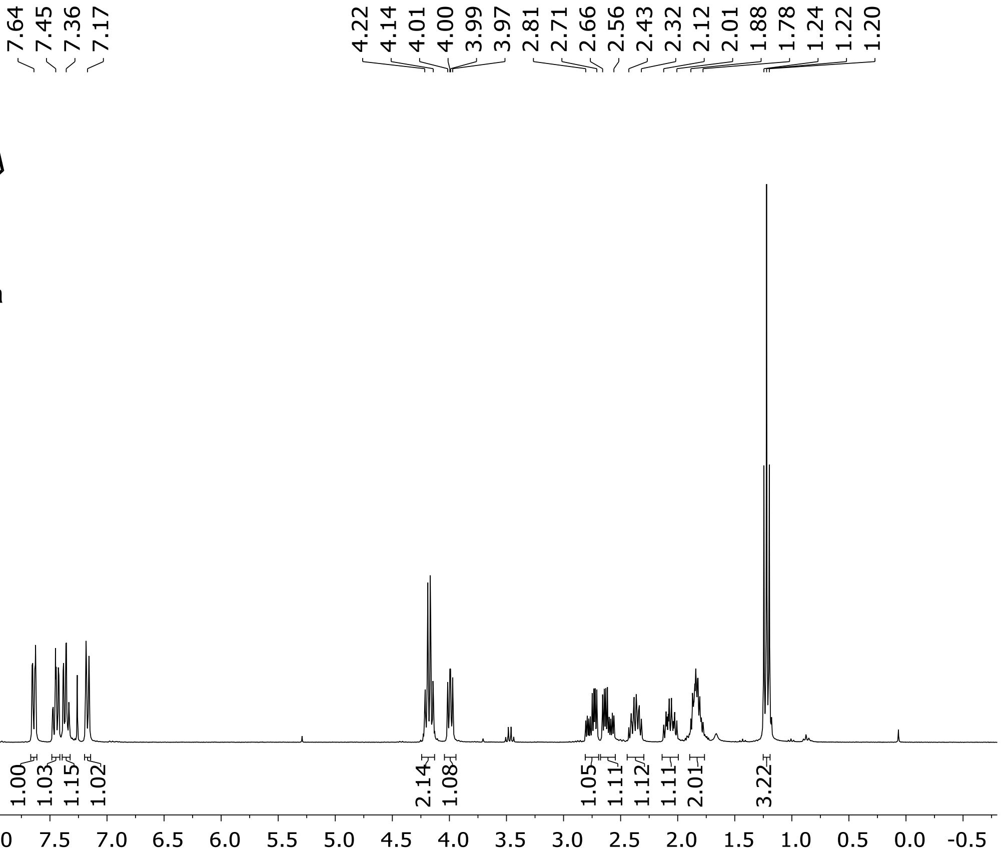

^{1}H NMR (300 MHz, $CDCl_3$) of compound **2a**.

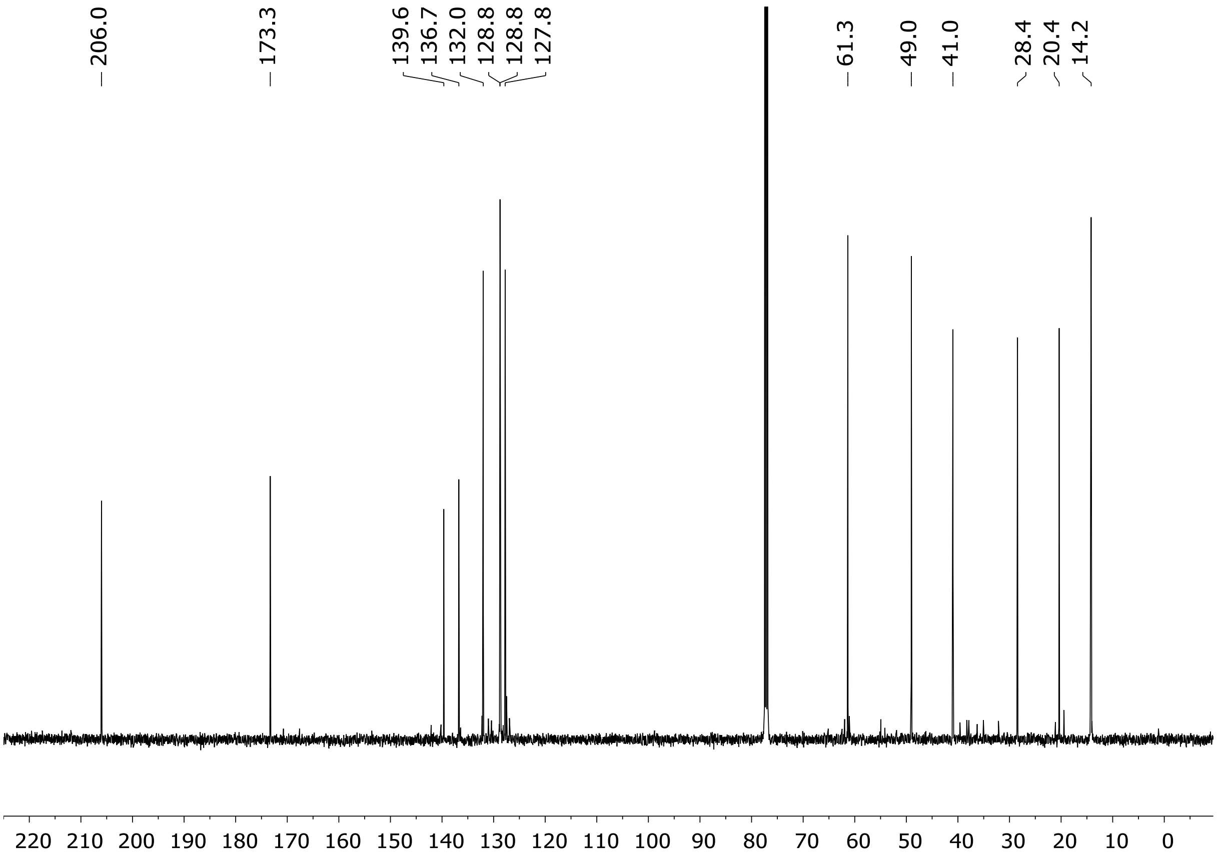

$^{13}C\{^{1}H\}$ NMR (125 MHz, $CDCl_3$) of compound **2a**.

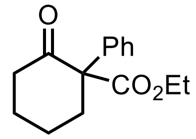

compound **8**

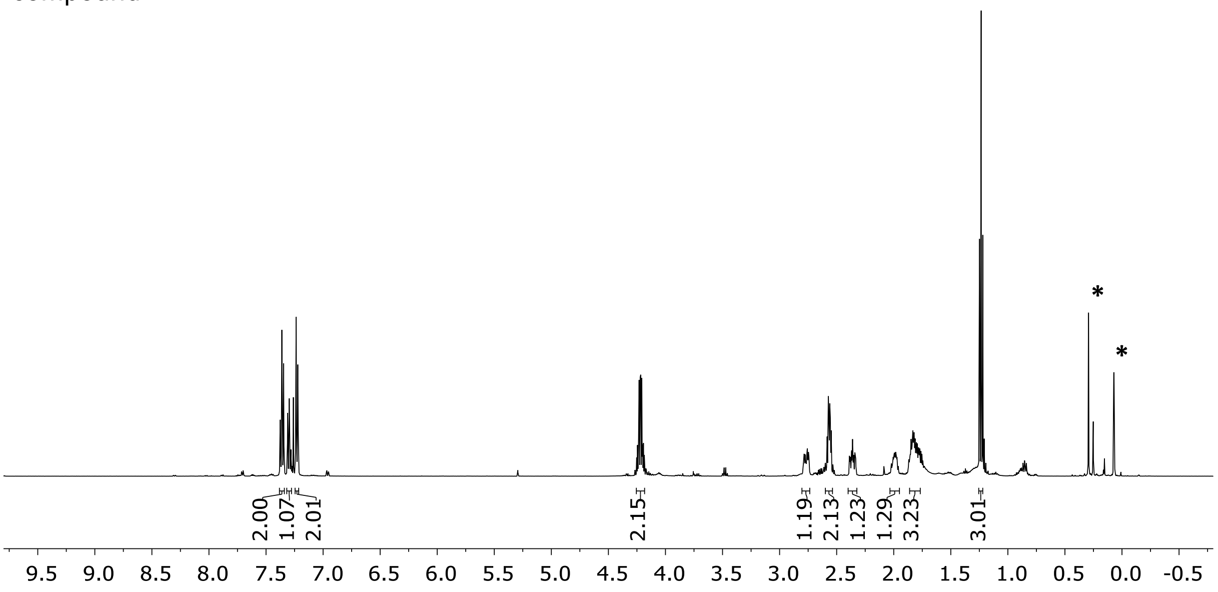

^{1}H NMR (500 MHz, $CDCl_3$) of compound **8**. *Impurities from the solvent.

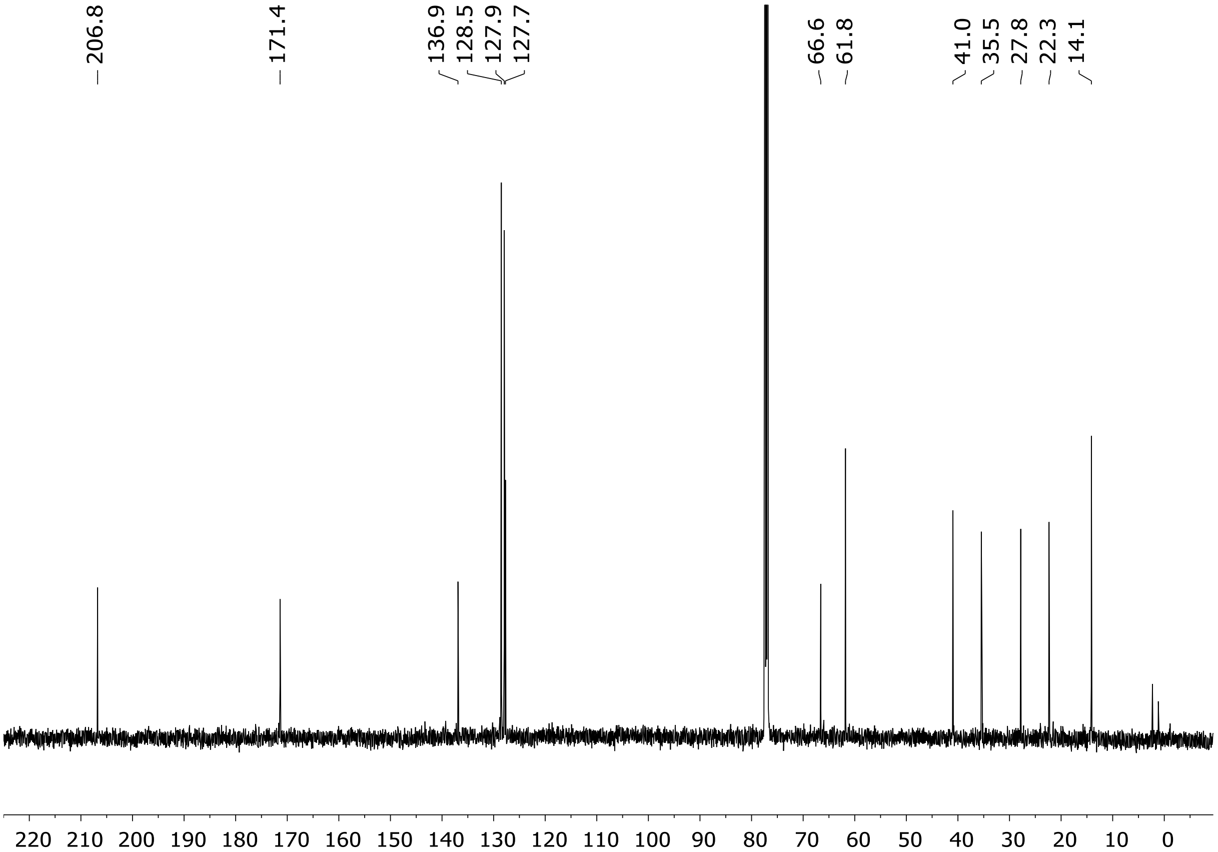

$^{13}C\{^{1}H\}$ NMR (125 MHz, $CDCl_3$) of compound **8**.

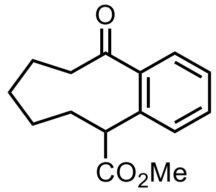

compound **2c**

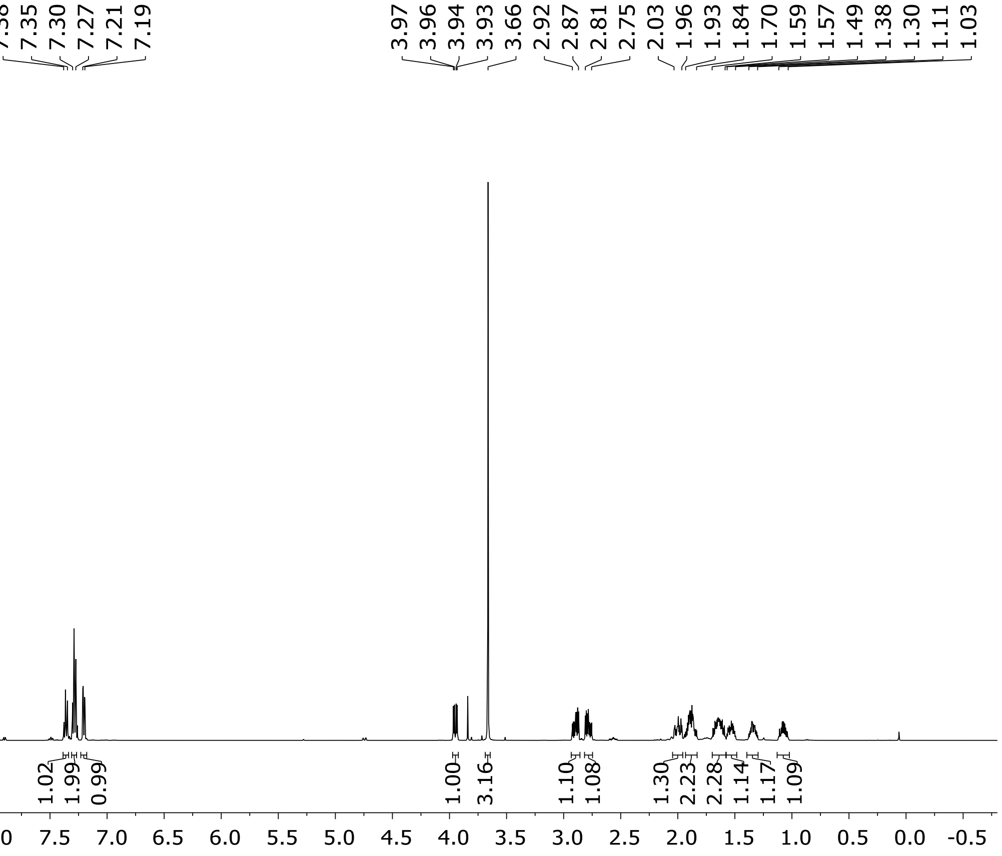

^{1}H NMR (500 MHz, $CDCl_3$) of compound **2c**.

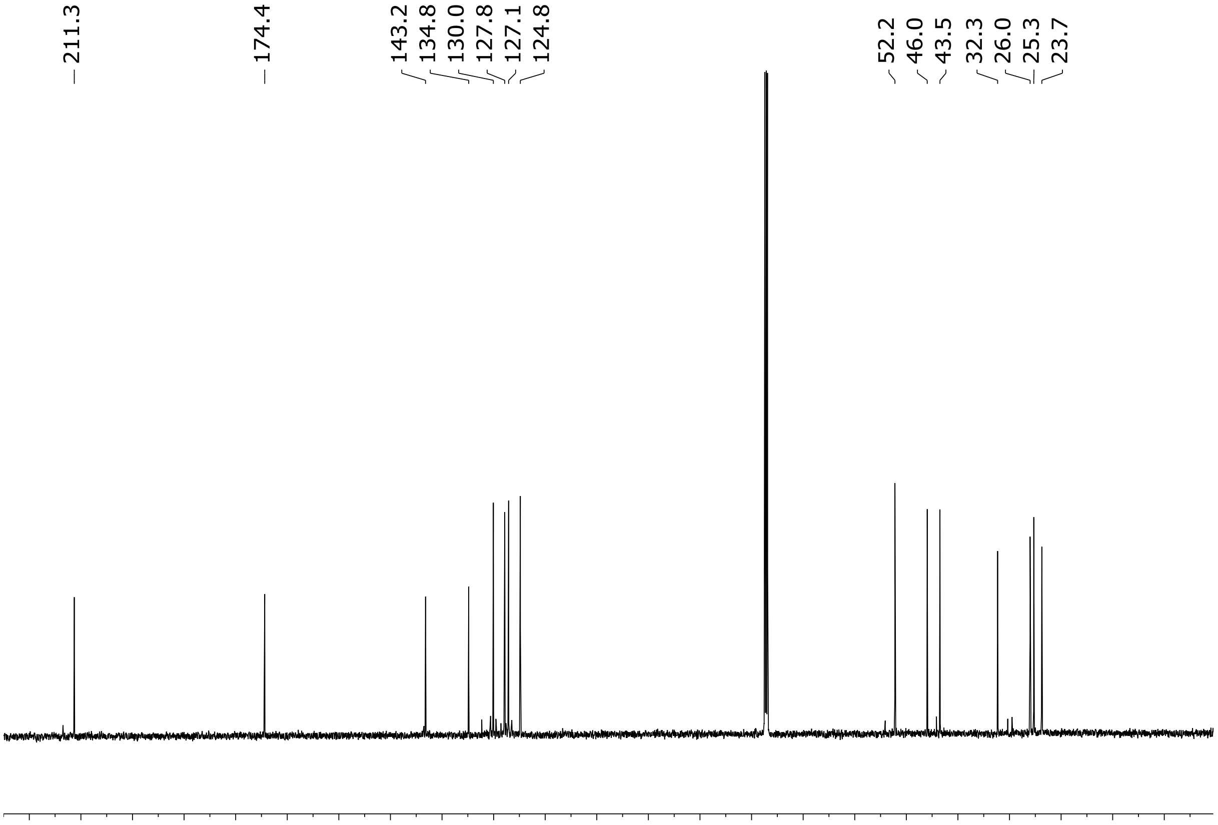

$^{13}C\{^{1}H\}$ NMR (125 MHz, $CDCl_3$) of compound **2c**.

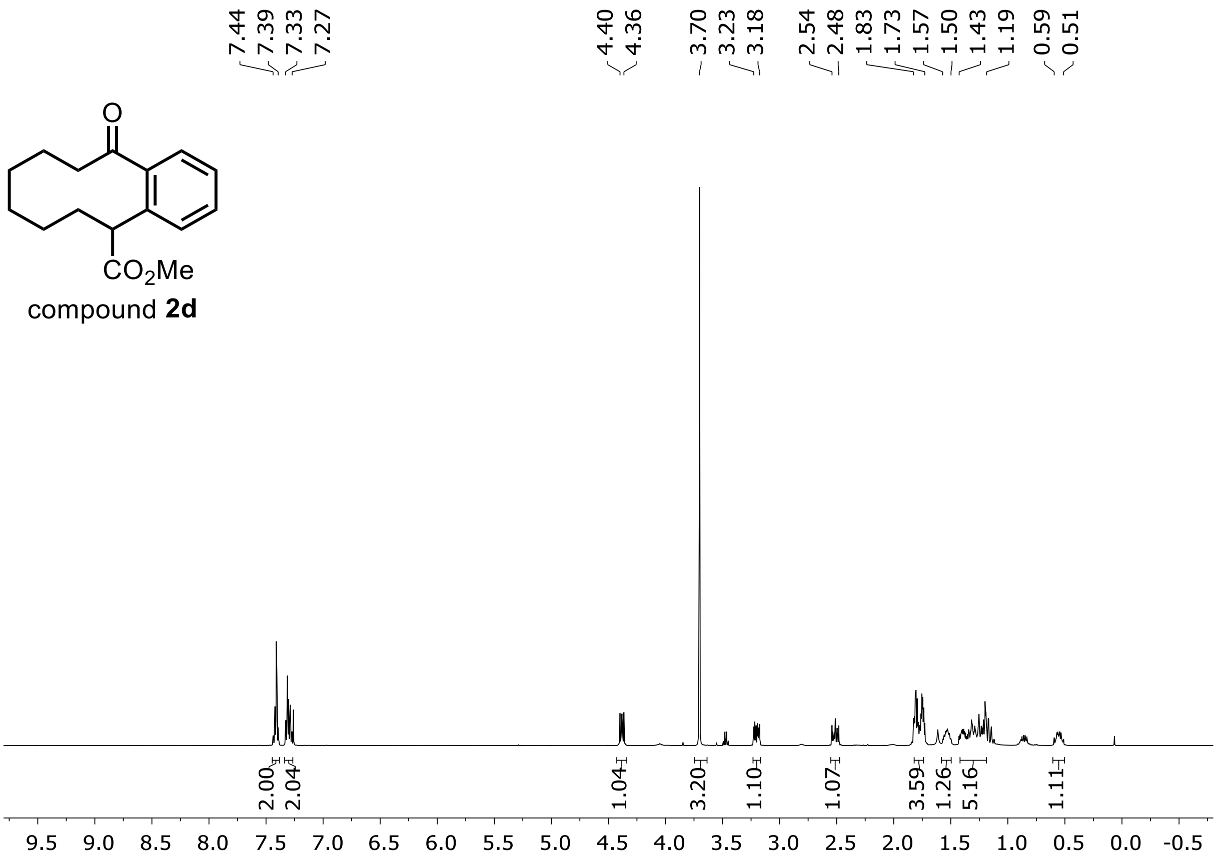

^{1}H NMR (500 MHz, $CDCl_3$) of compound **2d**.

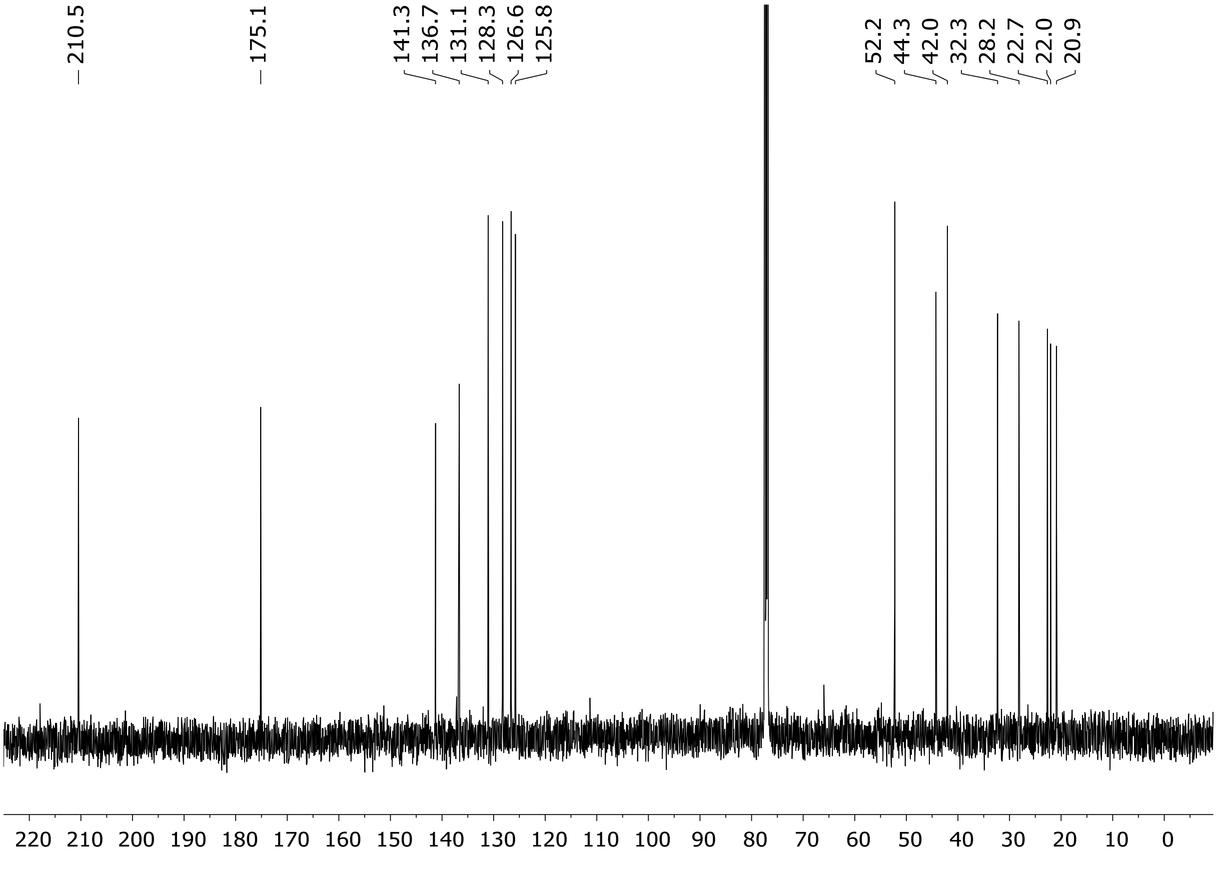

$^{13}C\{^1H\}$ NMR (125 MHz, $CDCl_3$) of compound **2d**.

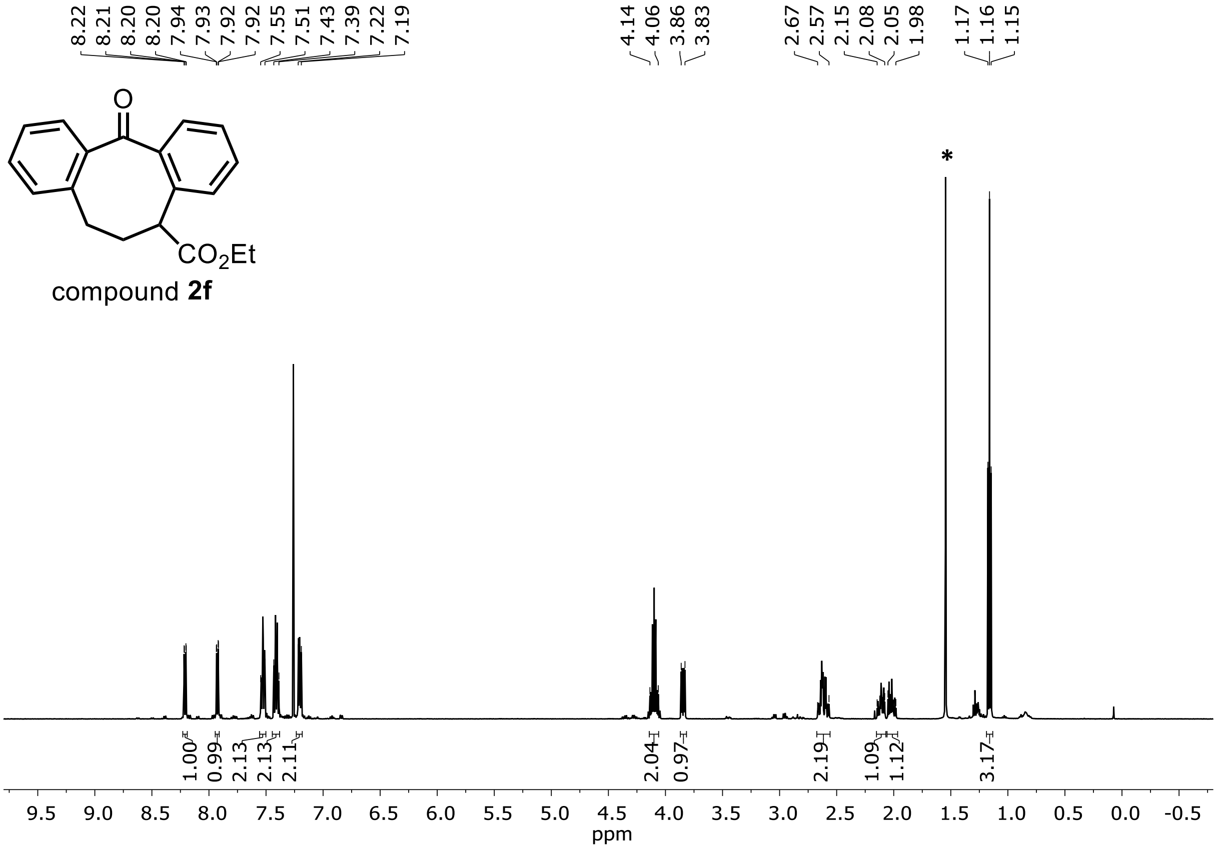

^{1}H NMR (500 MHz, $CDCl_3$) of compound **2f**. *Impurities from the solvent.

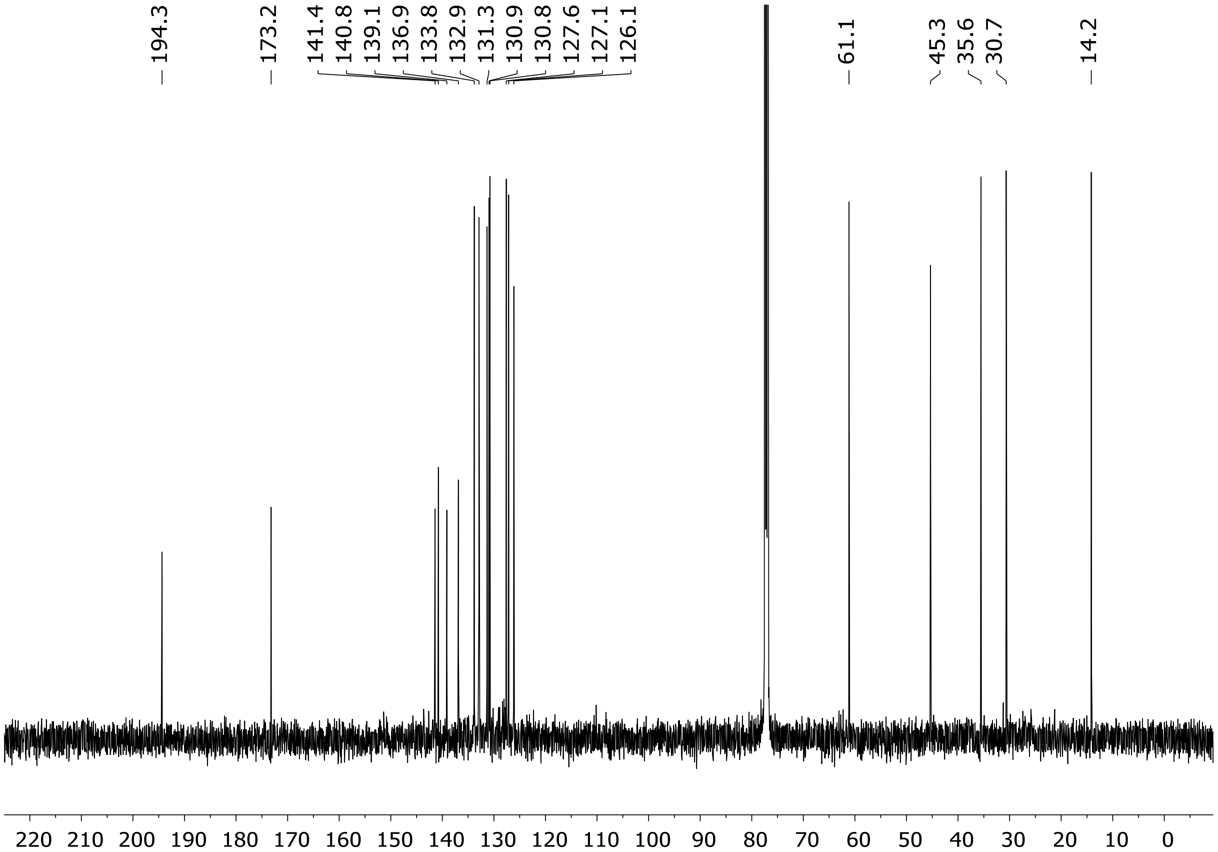

$^{13}C\{^{1}H\}$ NMR (125 MHz, $CDCl_3$) of compound **2f**.

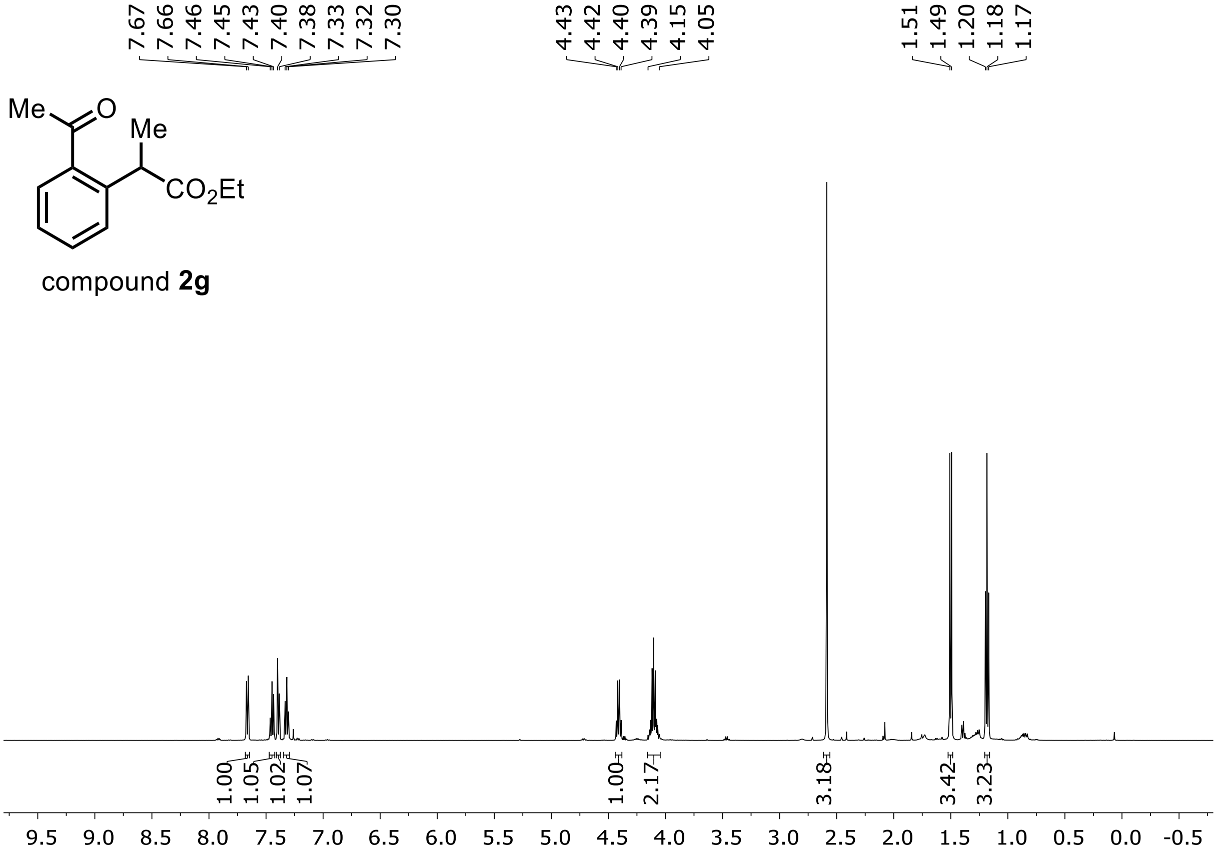

^{1}H NMR (500 MHz, $CDCl_3$) of compound **2g**.

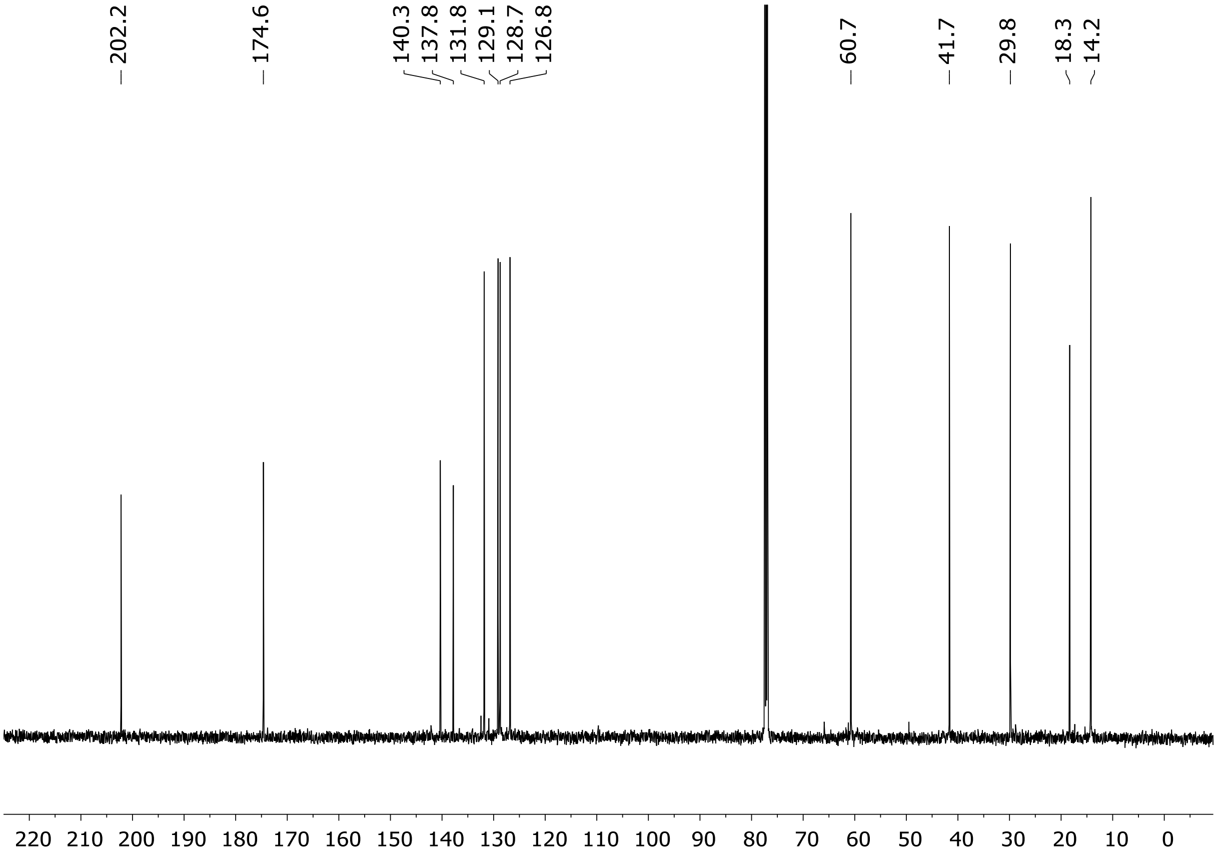

^{13}C{^{1}H} NMR (125 MHz, $CDCl_3$) of compound **2g**.

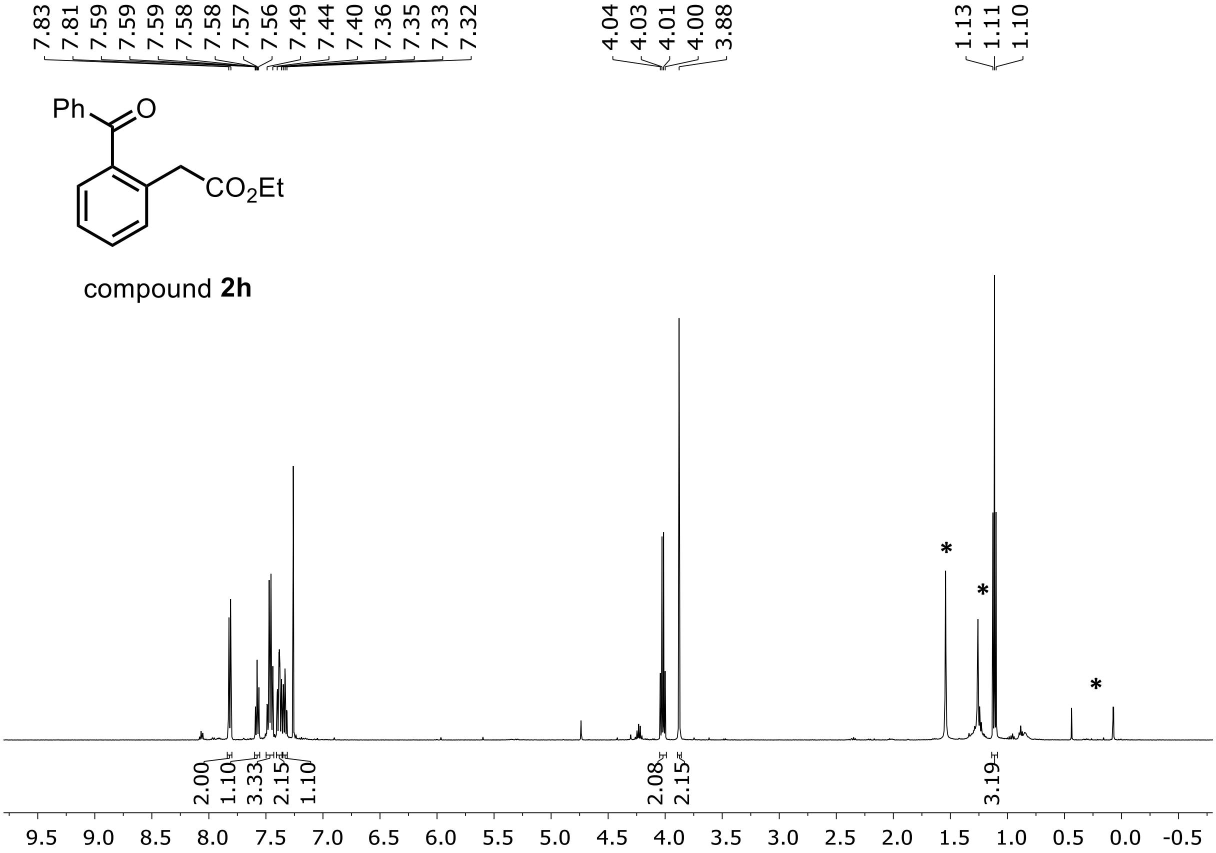

^{1}H NMR (500 MHz, $CDCl_3$) of compound **2h**. ***Impurities from the solvent.

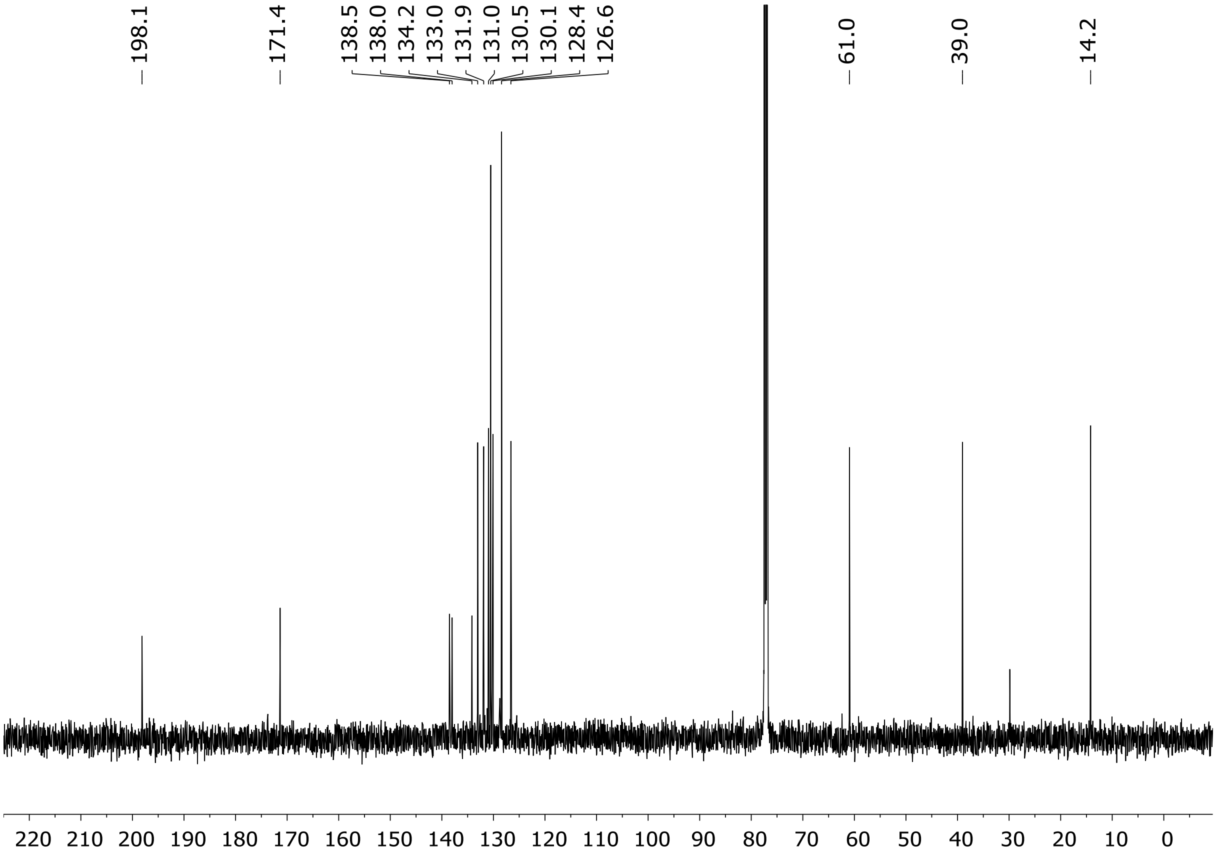

^{13}C{^{1}H} NMR (125 MHz, $CDCl_3$) of compound **2h**.

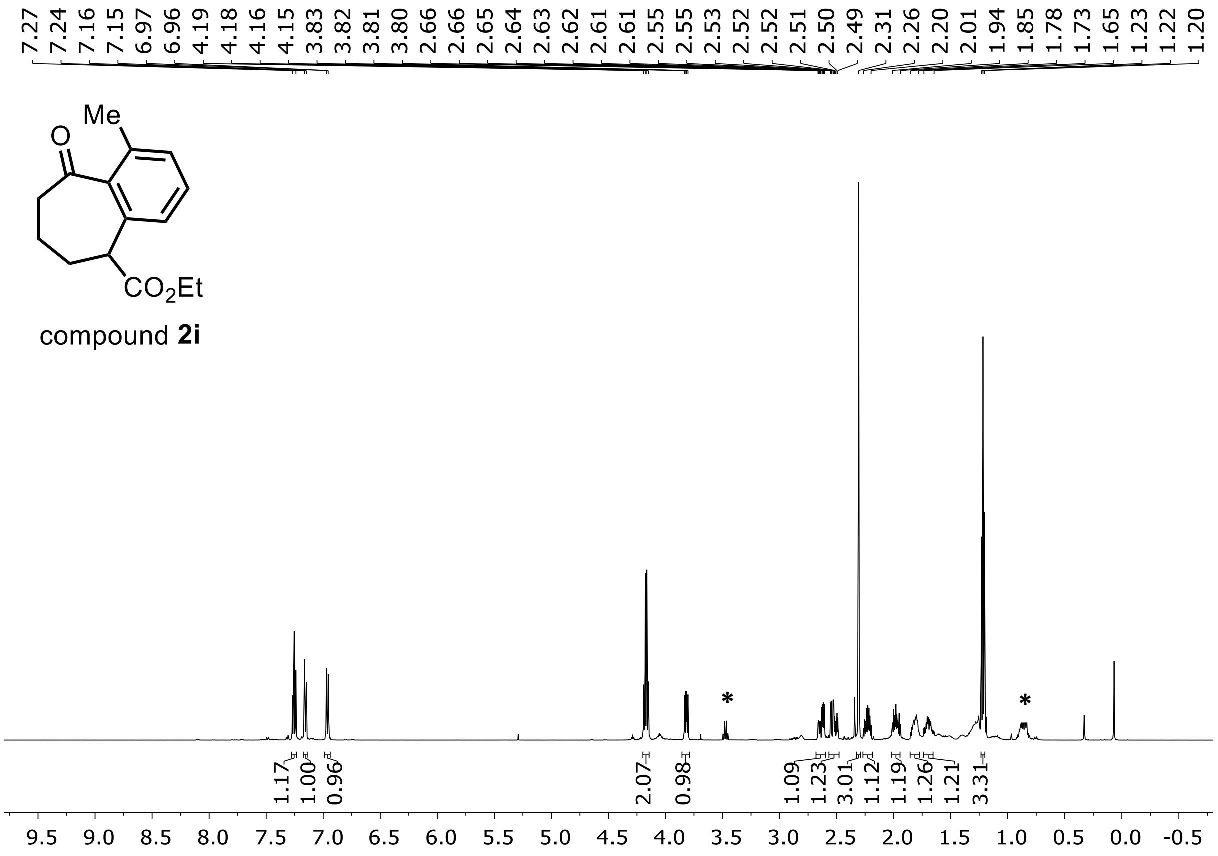

^{1}H NMR (500 MHz, $CDCl_3$) of compound **2i**. *Residual peaks from diethyl ether.

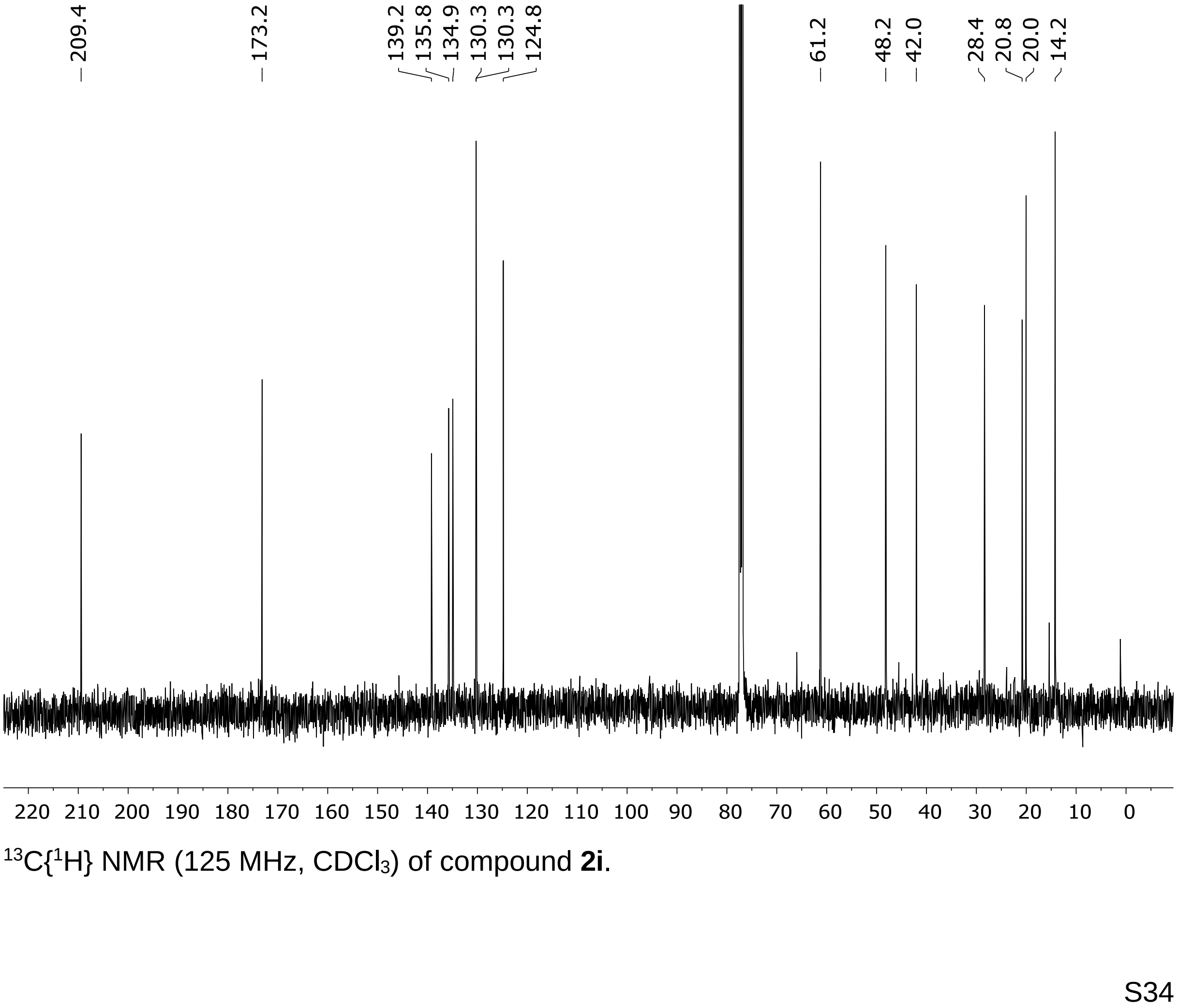

^{13}C{^{1}H} NMR (125 MHz, $CDCl_3$) of compound **2i**.

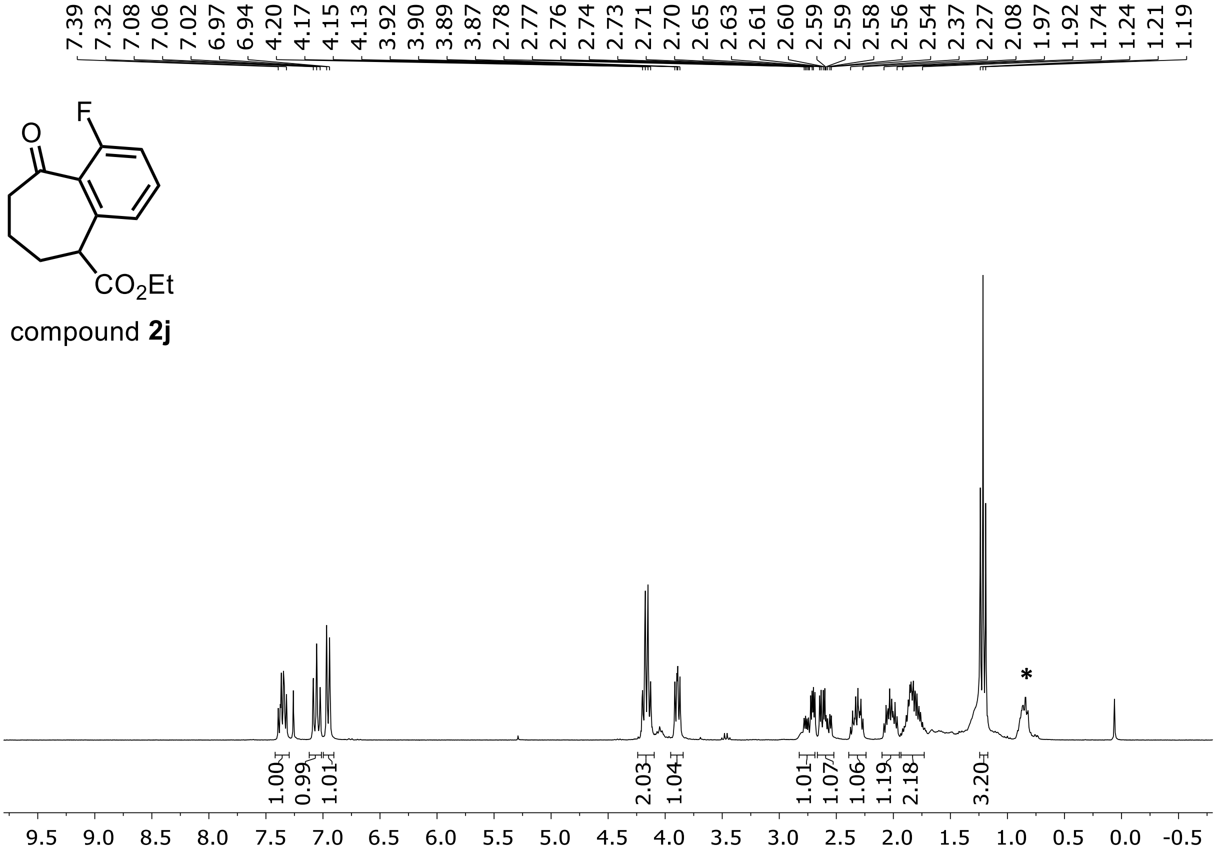

^{1}H NMR (500 MHz, $CDCl_3$) of compound **2j**. *Impurities from the solvent.

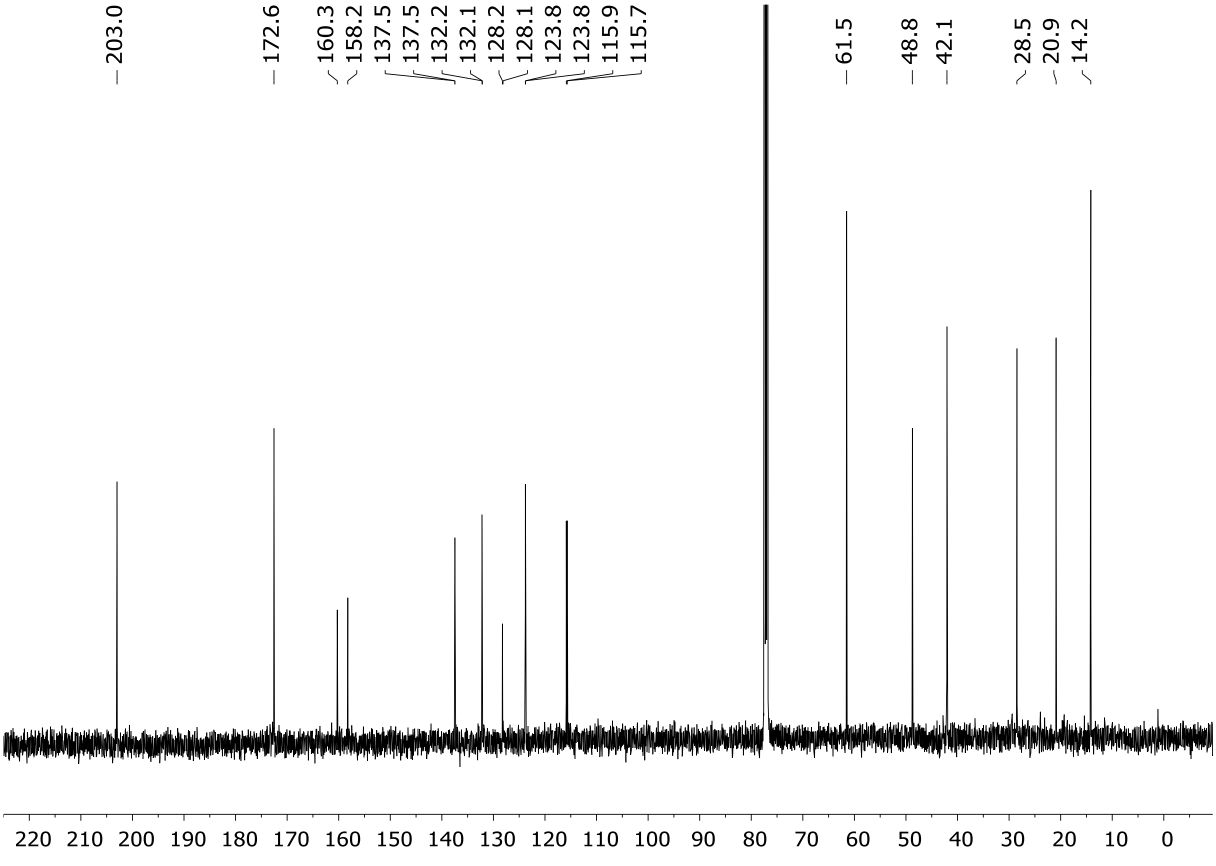

^{13}C{^{1}H} NMR (125 MHz, $CDCl_3$) of compound **2j**.

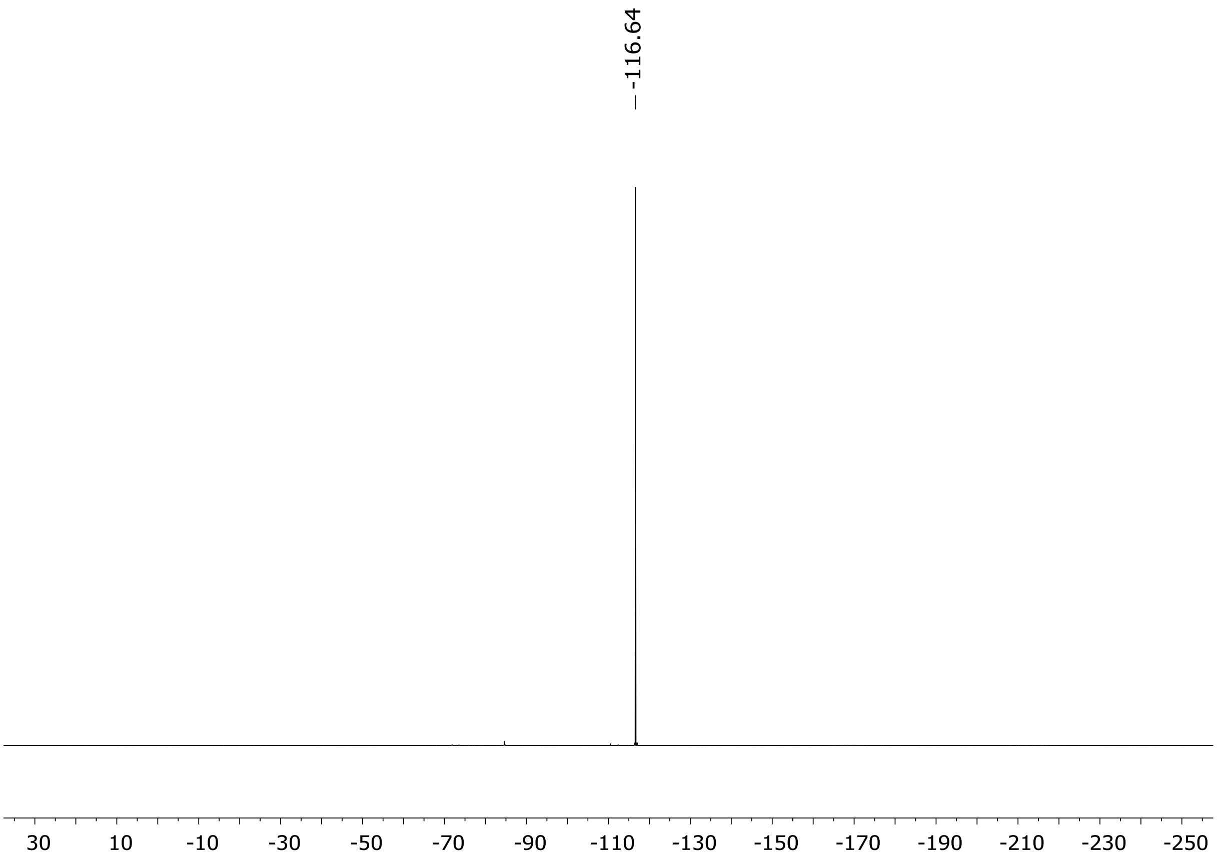

$^{19}F\{^{1}H\}$ NMR (470 MHz, $CDCl_3$) of compound **2j**.

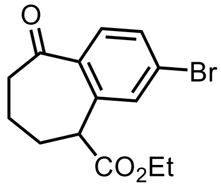

compound **2k**

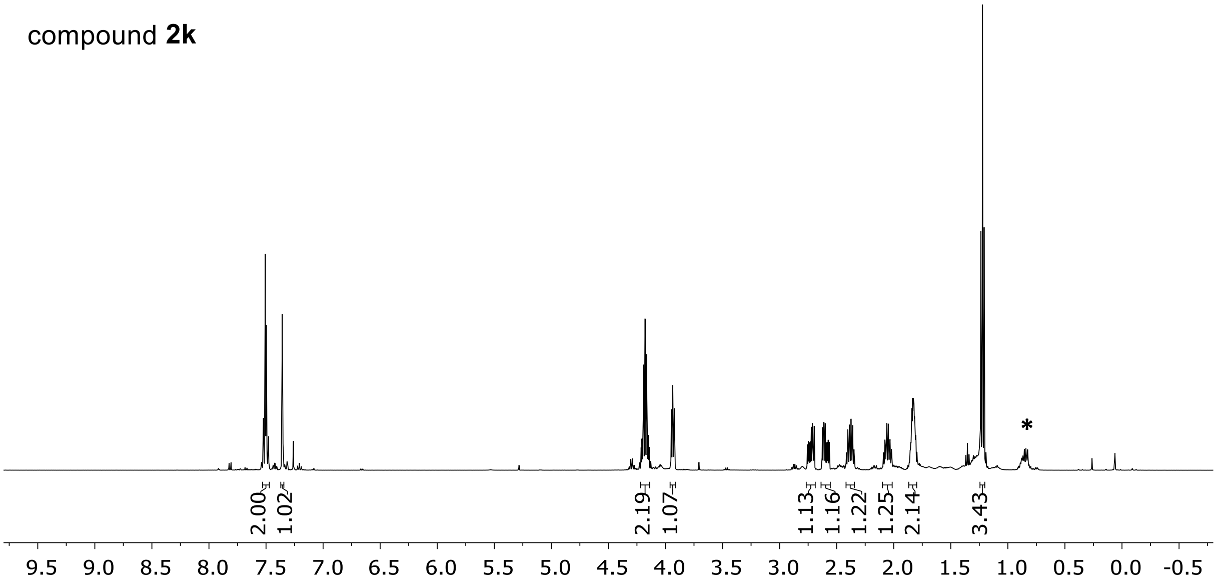

^{1}H NMR (500 MHz, $CDCl_3$) of compound **2k**. ***Impurities from the solvent.

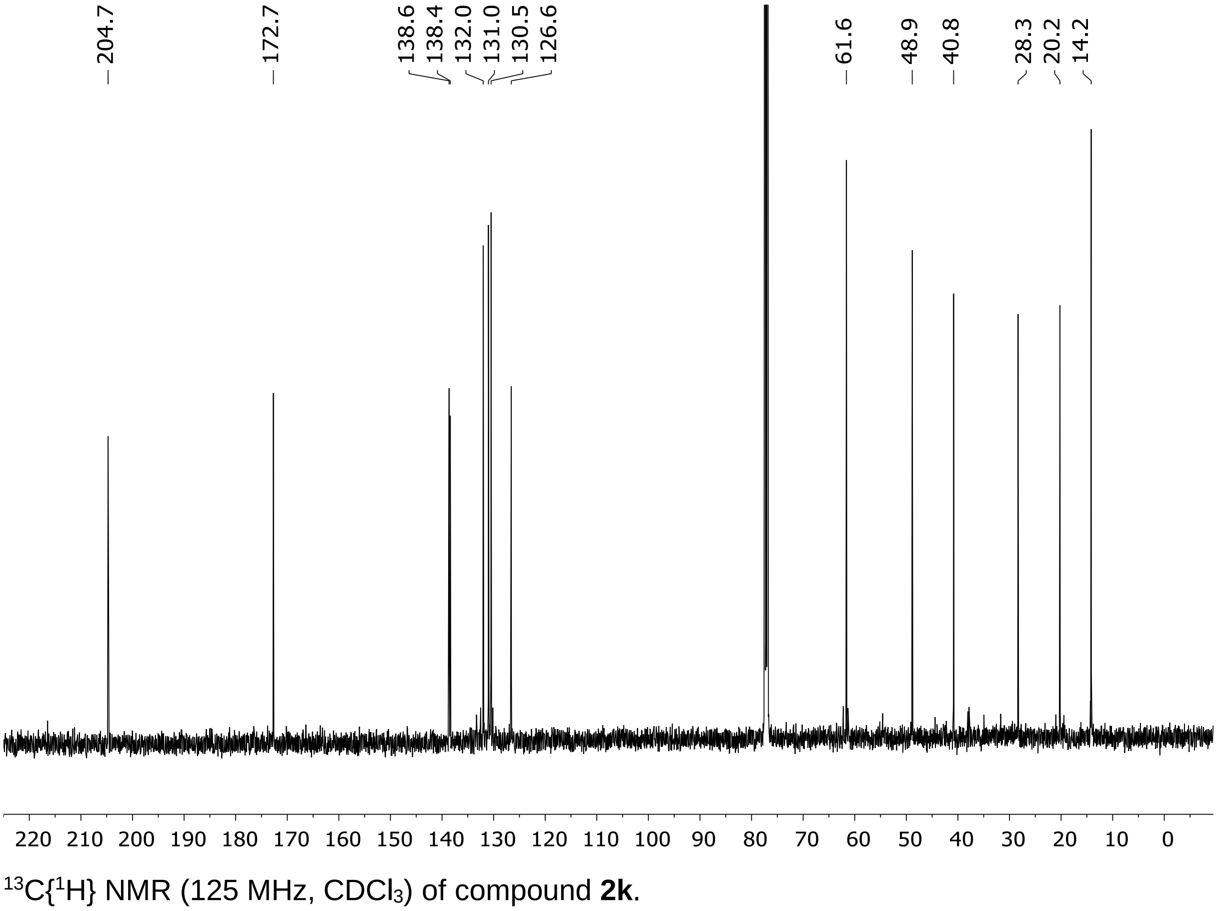

$^{13}C\{^{1}H\}$ NMR (125 MHz, $CDCl_3$) of compound **2k**.

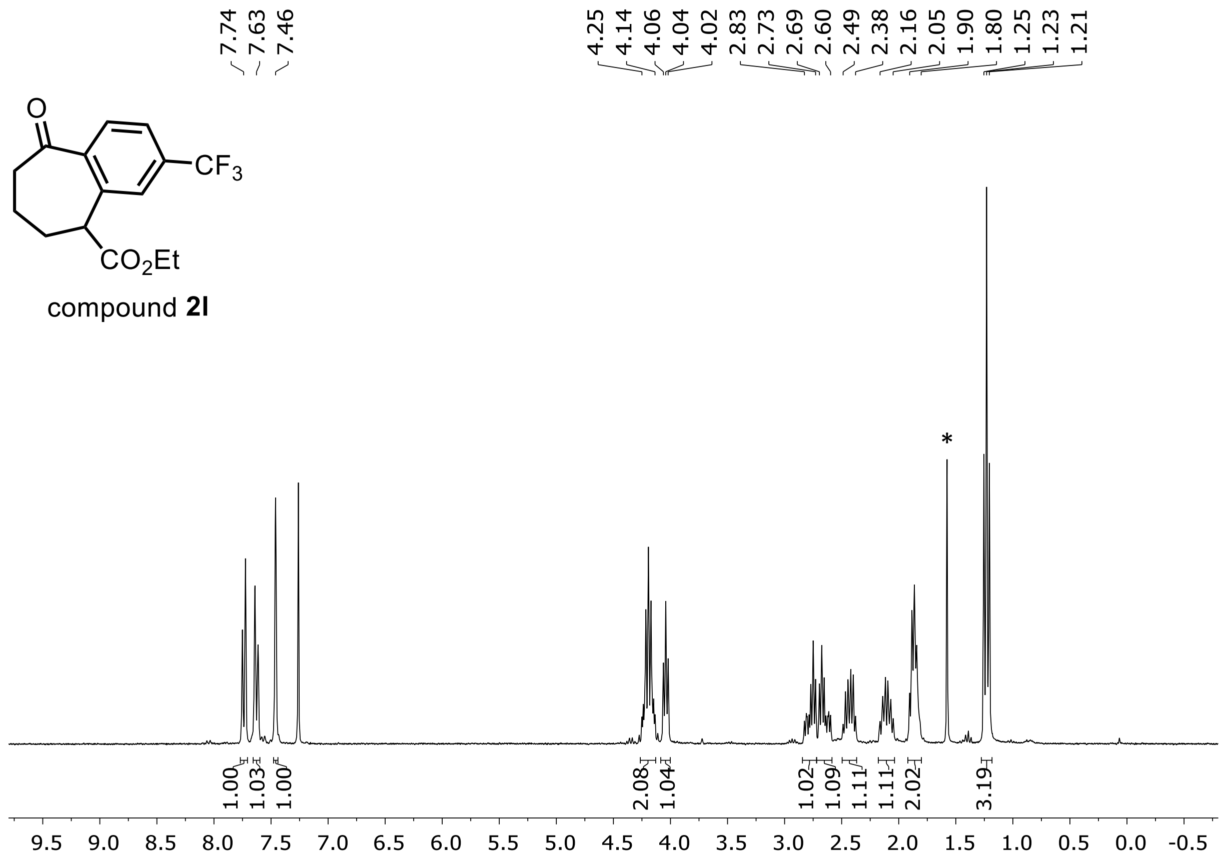

^{1}H NMR (300 MHz, $CDCl_3$) of compound **2l**. *Impurities from the solvent.

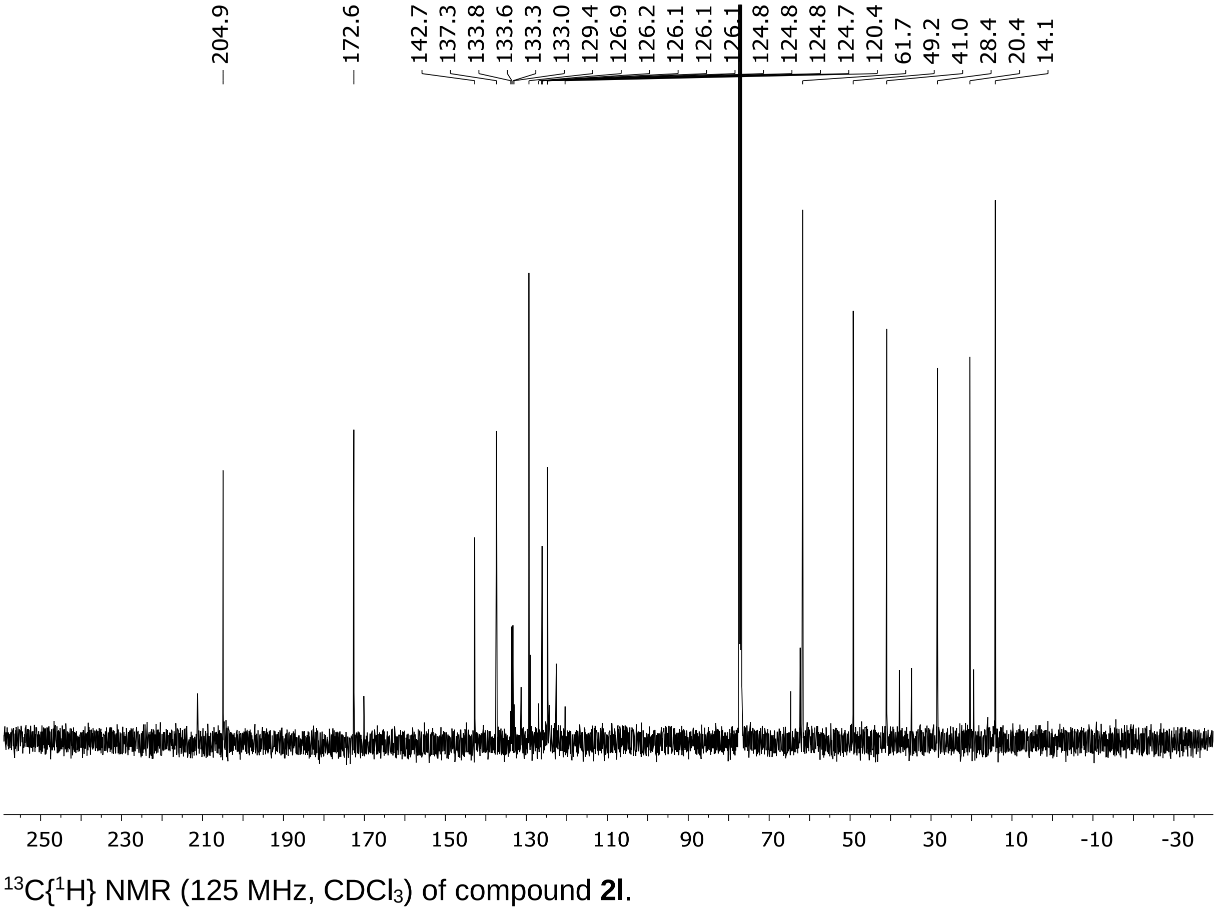

$^{13}C\{^{1}H\}$ NMR (125 MHz, $CDCl_3$) of compound **2l**.

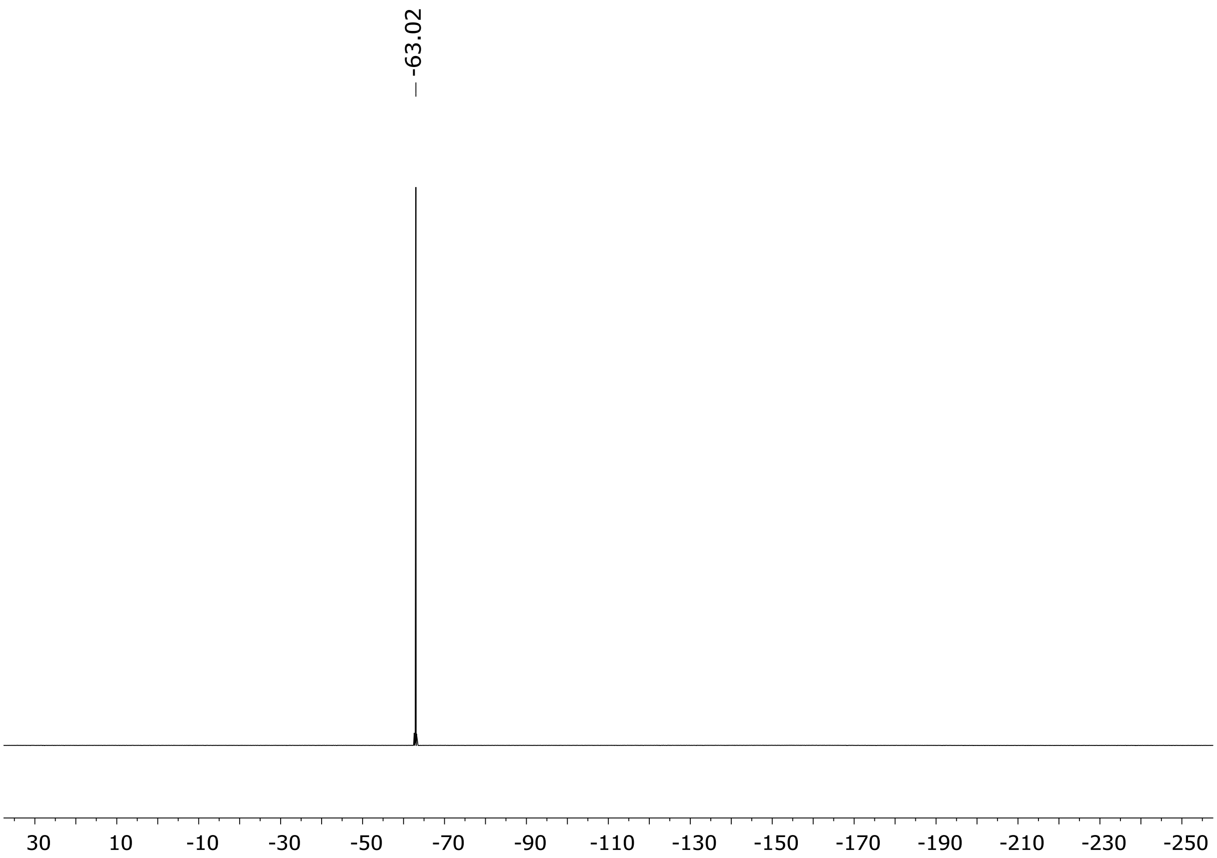

$^{19}F\{^{1}H\}$ NMR (470 MHz, $CDCl_3$) of compound **2l**.

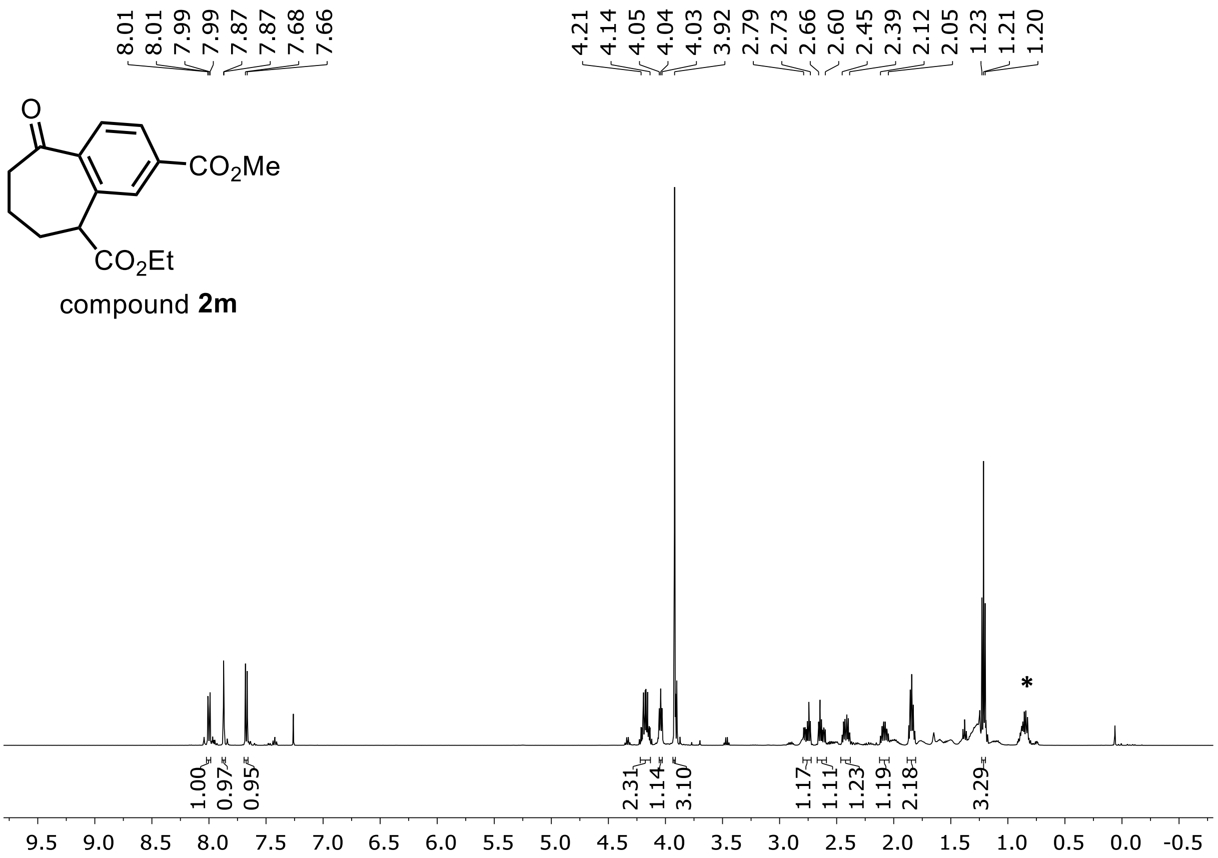

^{1}H NMR (500 MHz, $CDCl_3$) of compound **2m**. ***Impurities from the solvent.

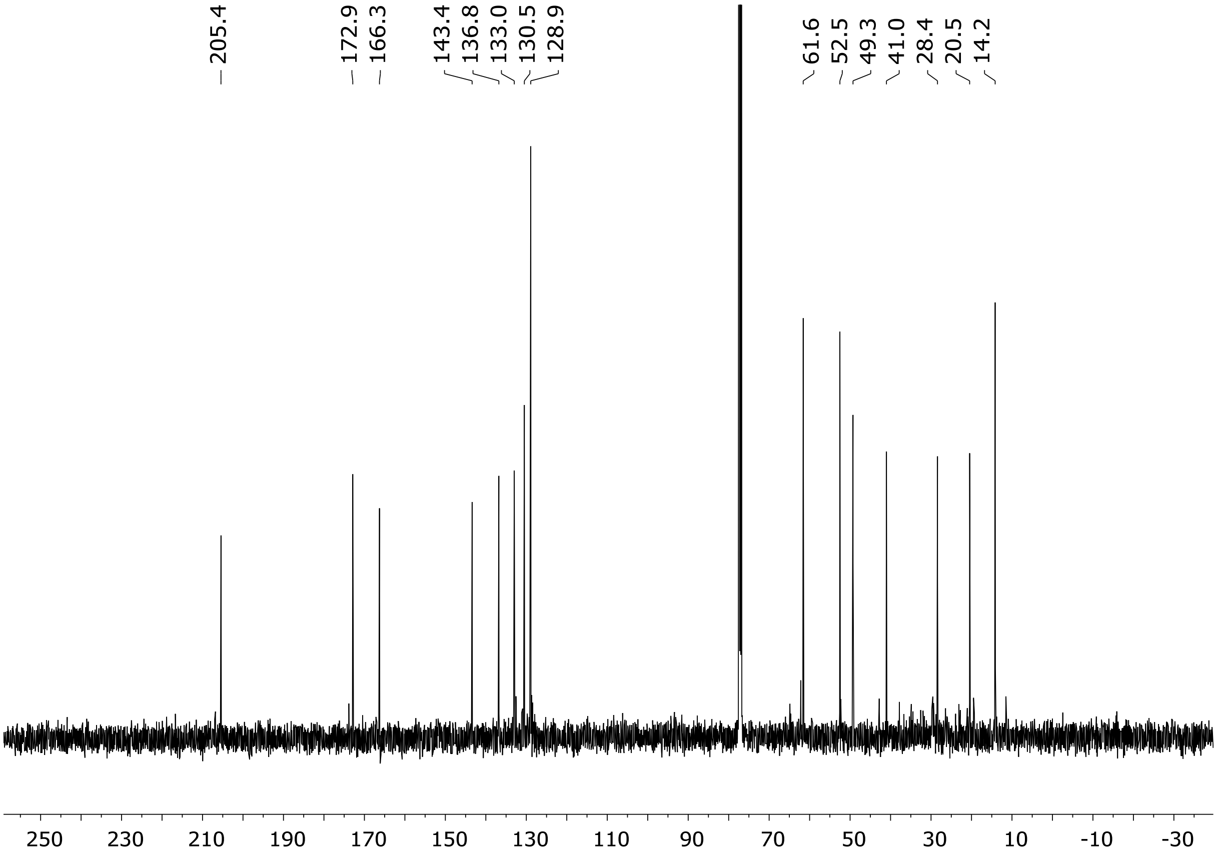

^{13}C{^{1}H} NMR (125 MHz, $CDCl_3$) of compound **2m**.

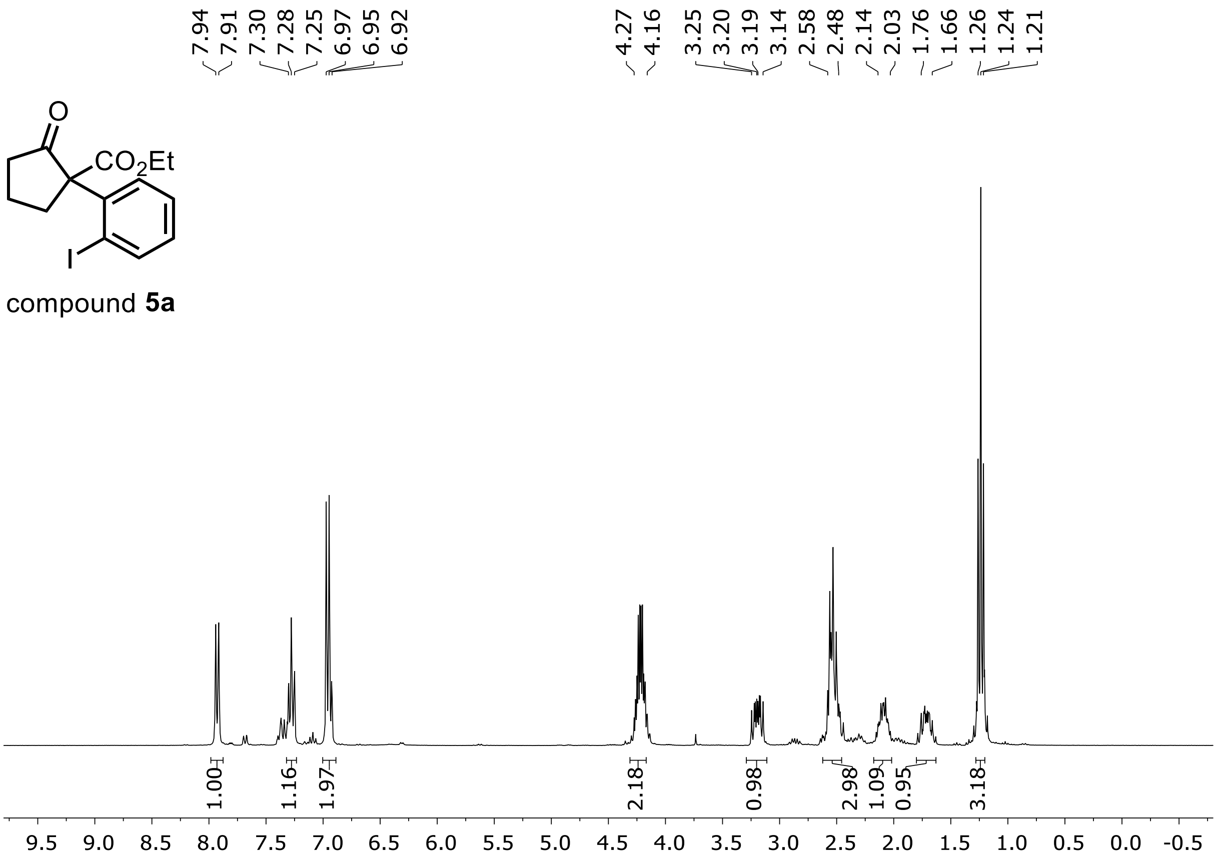

^{1}H NMR (300 MHz, $CDCl_3$) of compound **5a**.

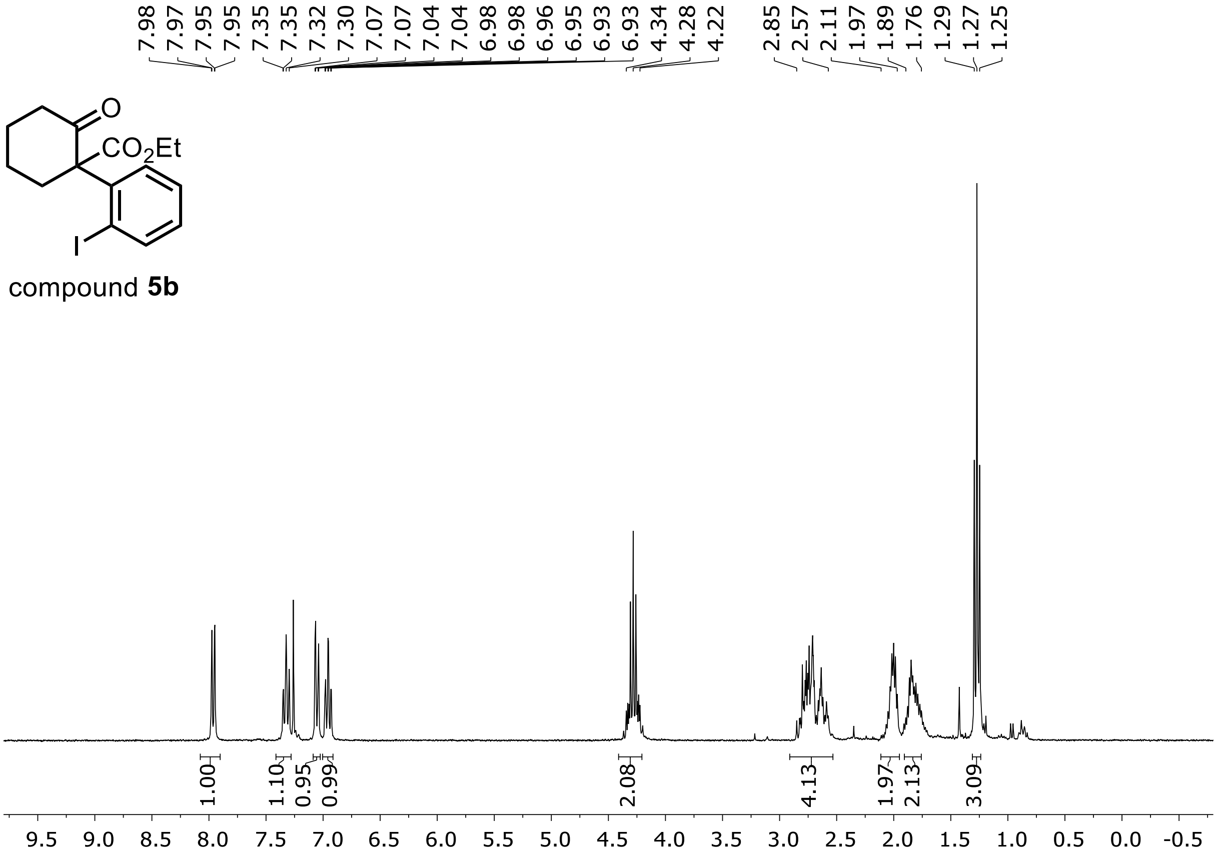

^{1}H NMR (300 MHz, $CDCl_3$) of compound **5b**.

compound **5c**

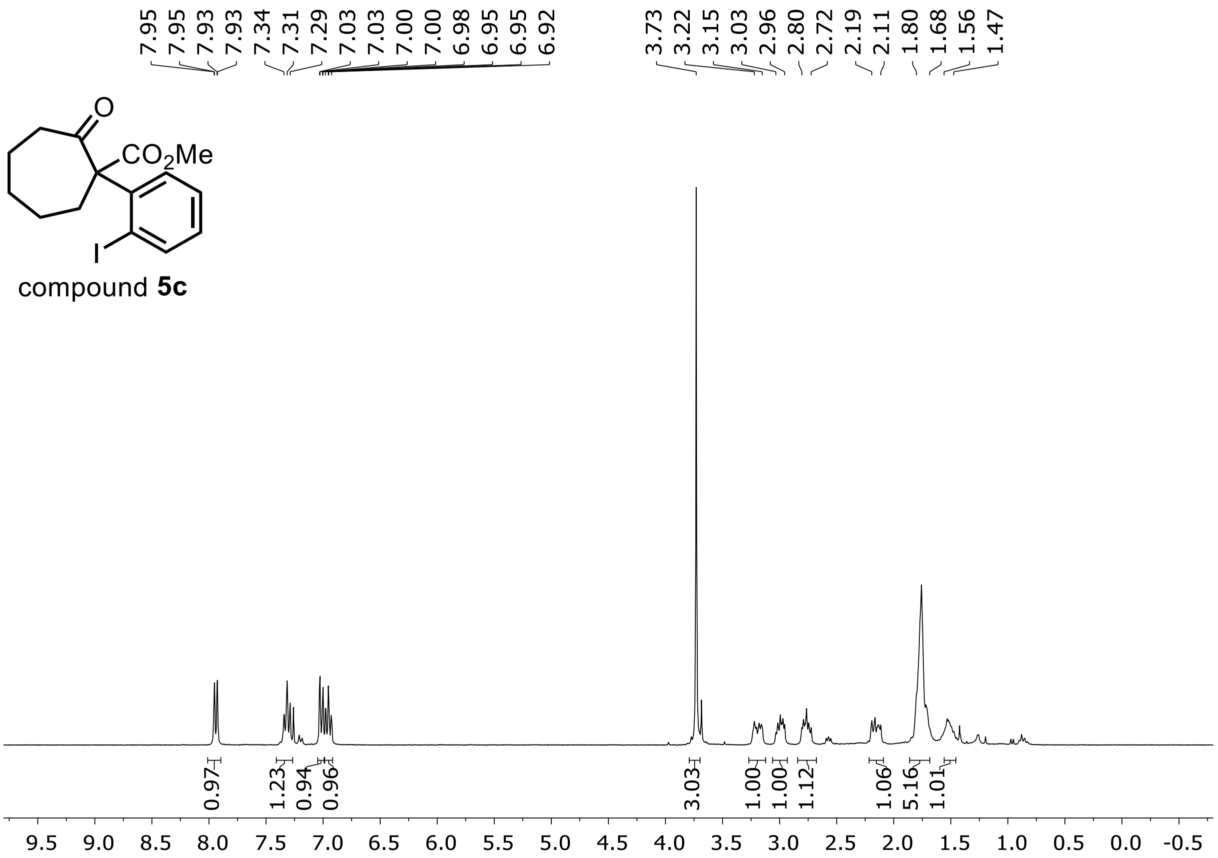

^{1}H NMR (300 MHz, $CDCl_3$) of compound **5c**.

compound **1d**

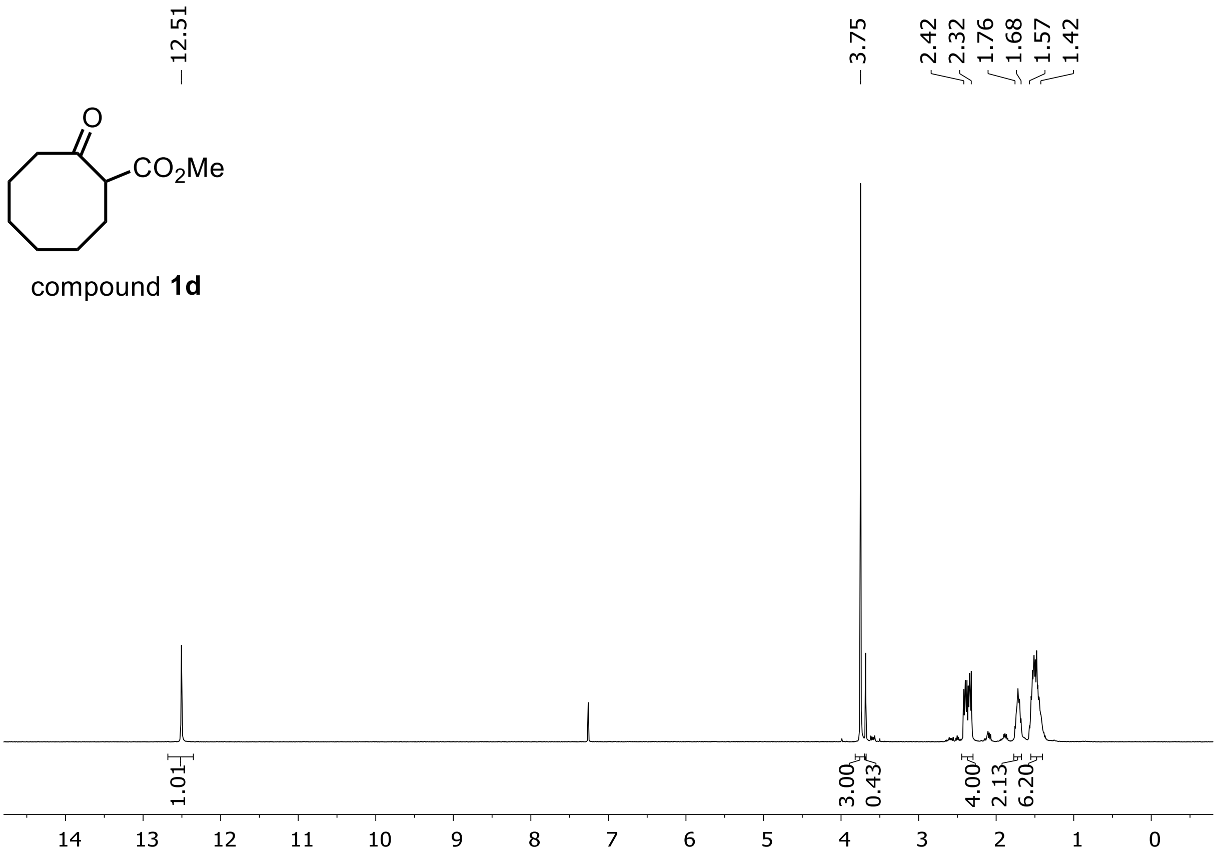

^{1}H NMR (300 MHz, $CDCl_3$) of compound **1d**.

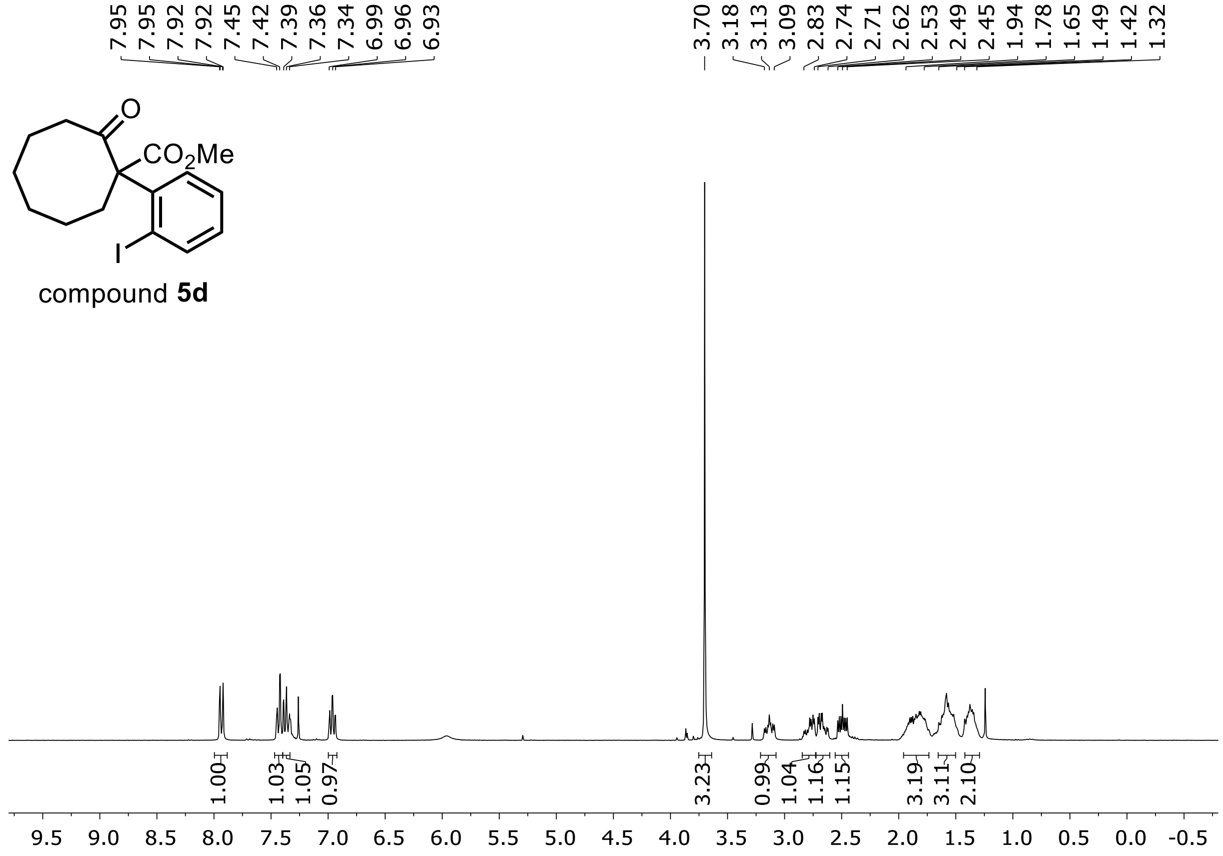

^{1}H NMR (300 MHz, $CDCl_3$) of compound **5d**.

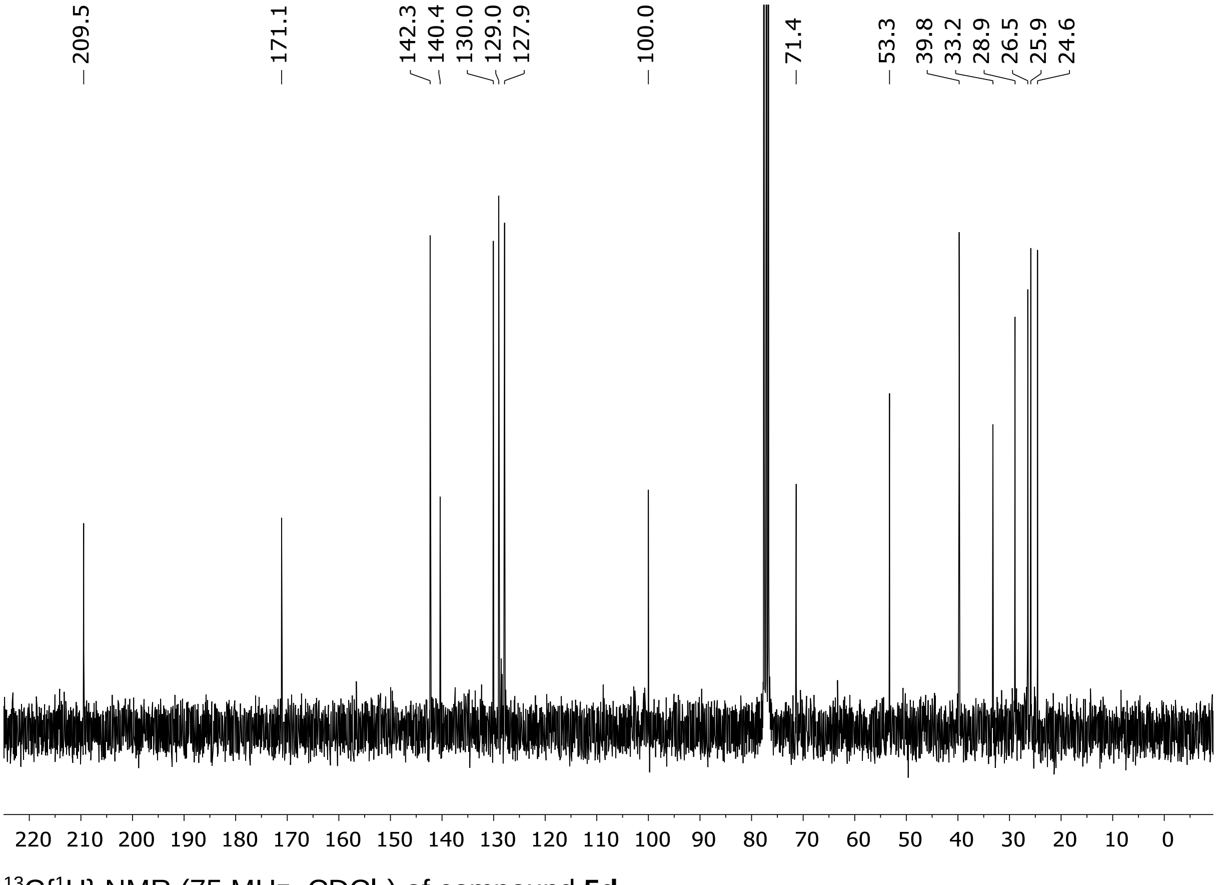

$^{13}C\{^{1}H\}$ NMR (75 MHz, $CDCl_3$) of compound **5d**.

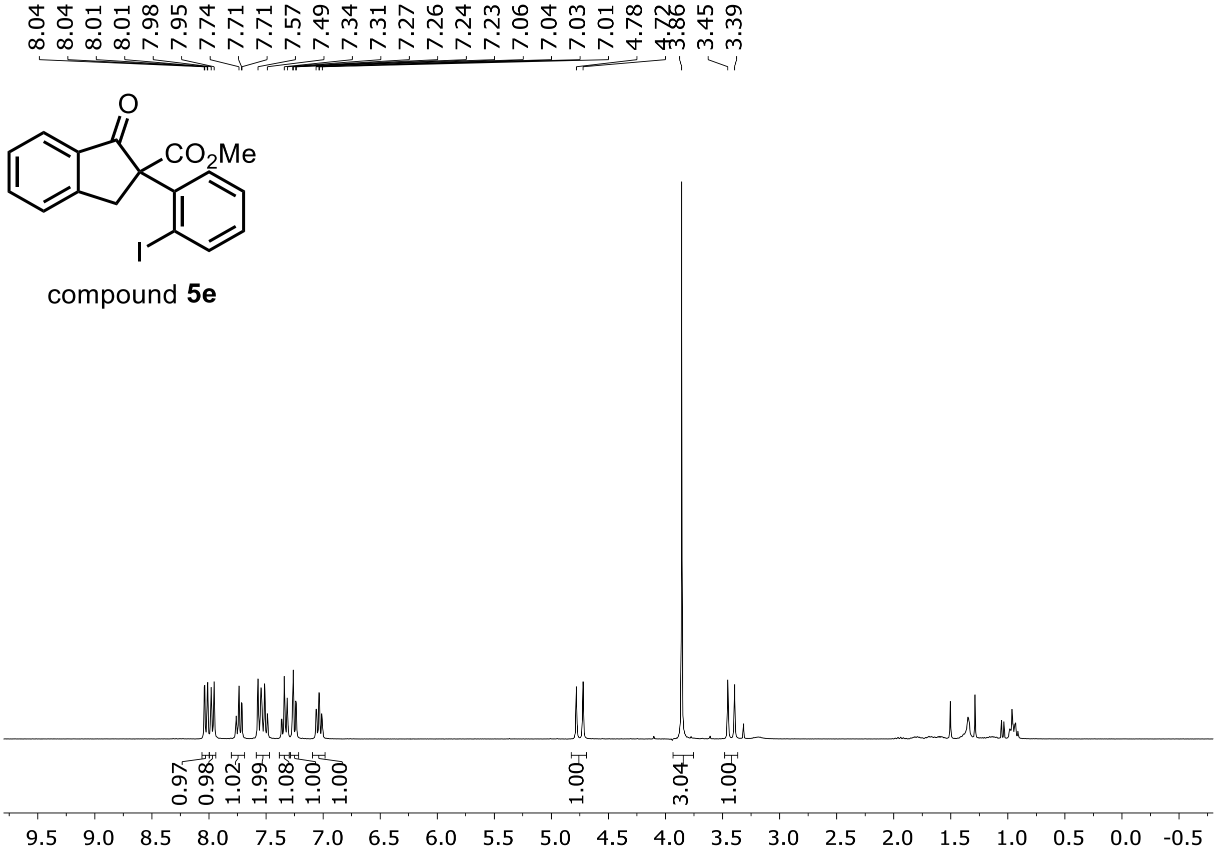

^{1}H NMR (300 MHz, $CDCl_3$) of compound **5e**.

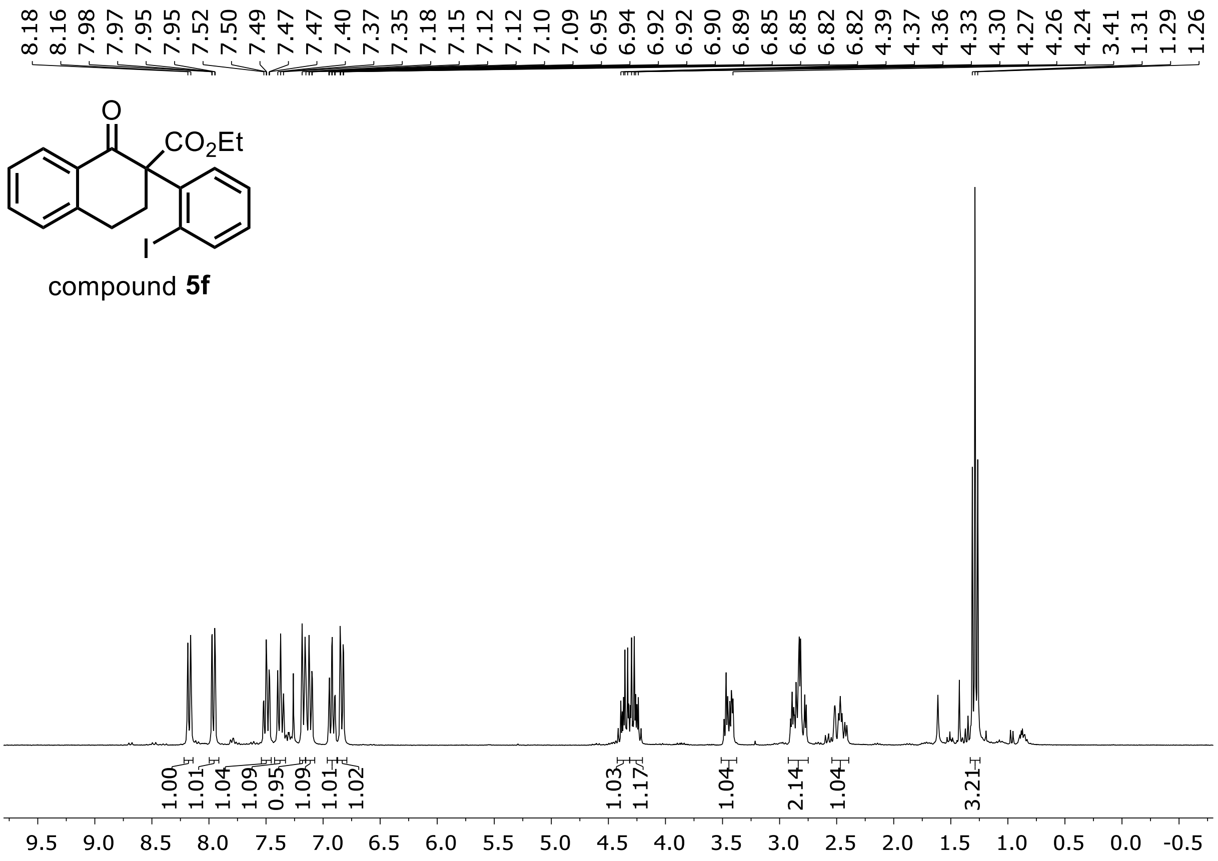

^{1}H NMR (300 MHz, $CDCl_3$) of compound **5f**.

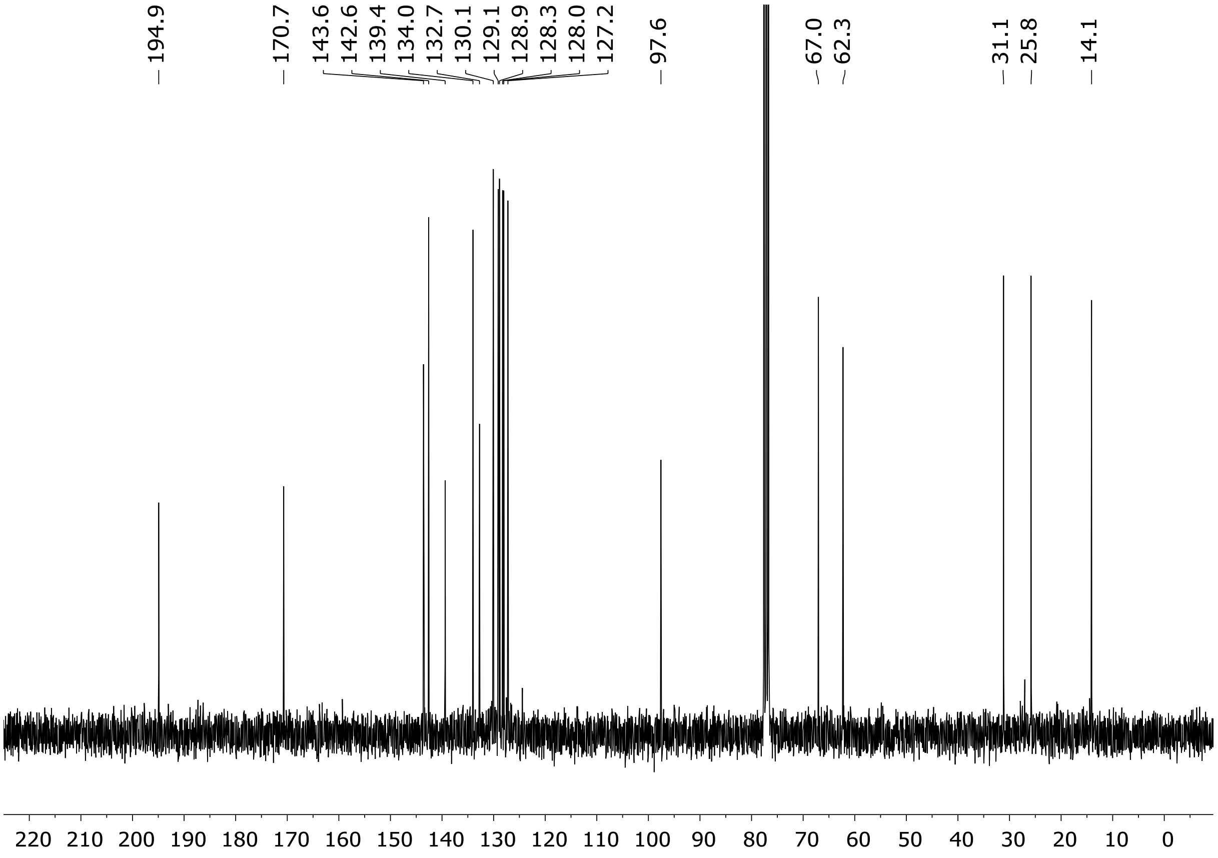

^{13}C{^{1}H} NMR (75 MHz, $CDCl_3$) compound **5f**.

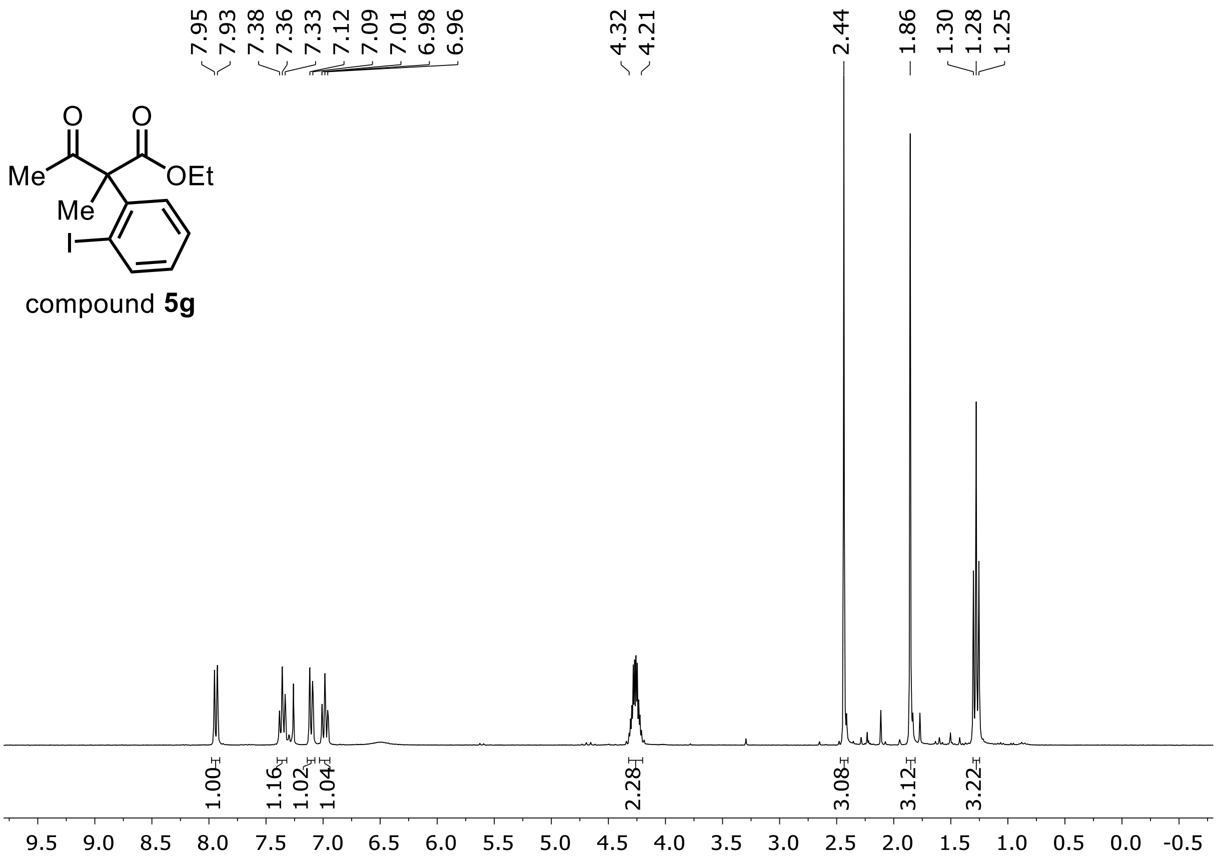

^{1}H NMR (300 MHz, $CDCl_3$) of compound **5g**.

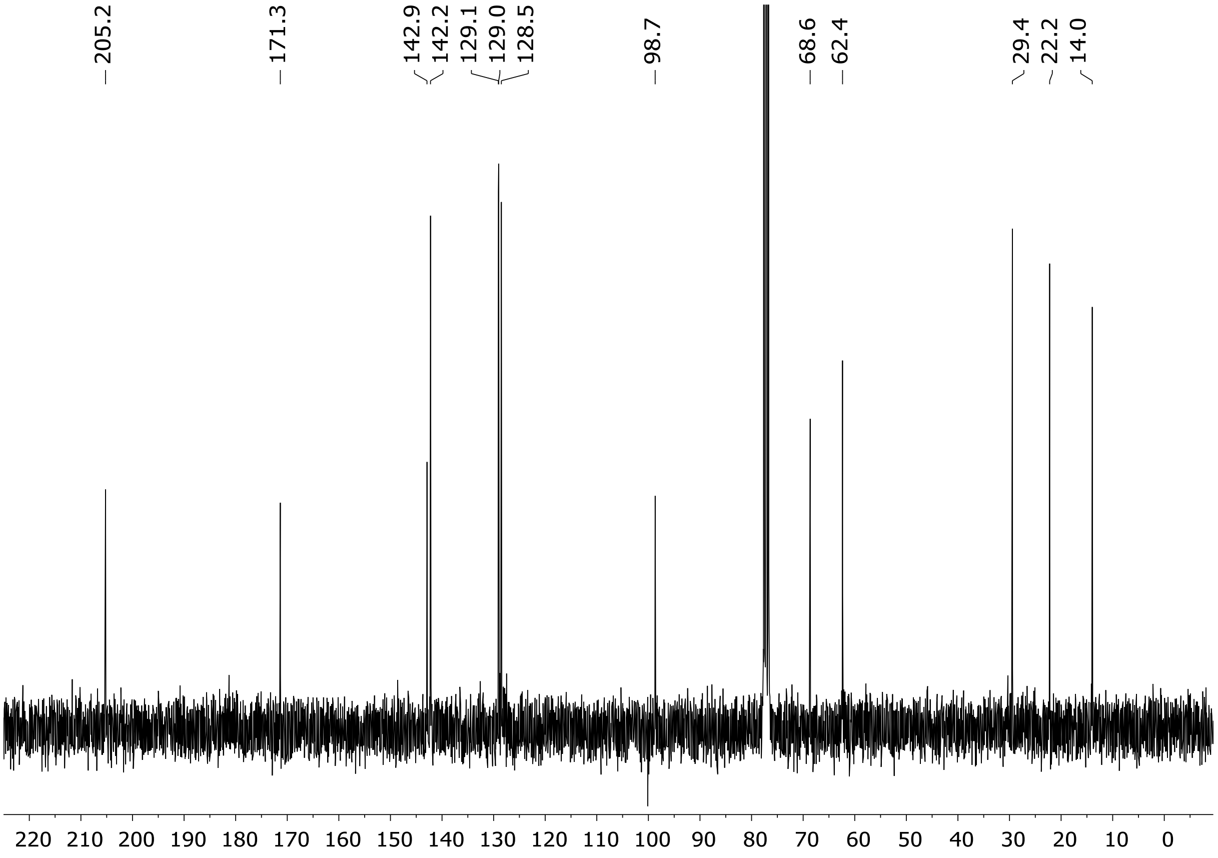

$^{13}C\{^1H\}$ NMR (75 MHz, $CDCl_3$) compound **5g**.

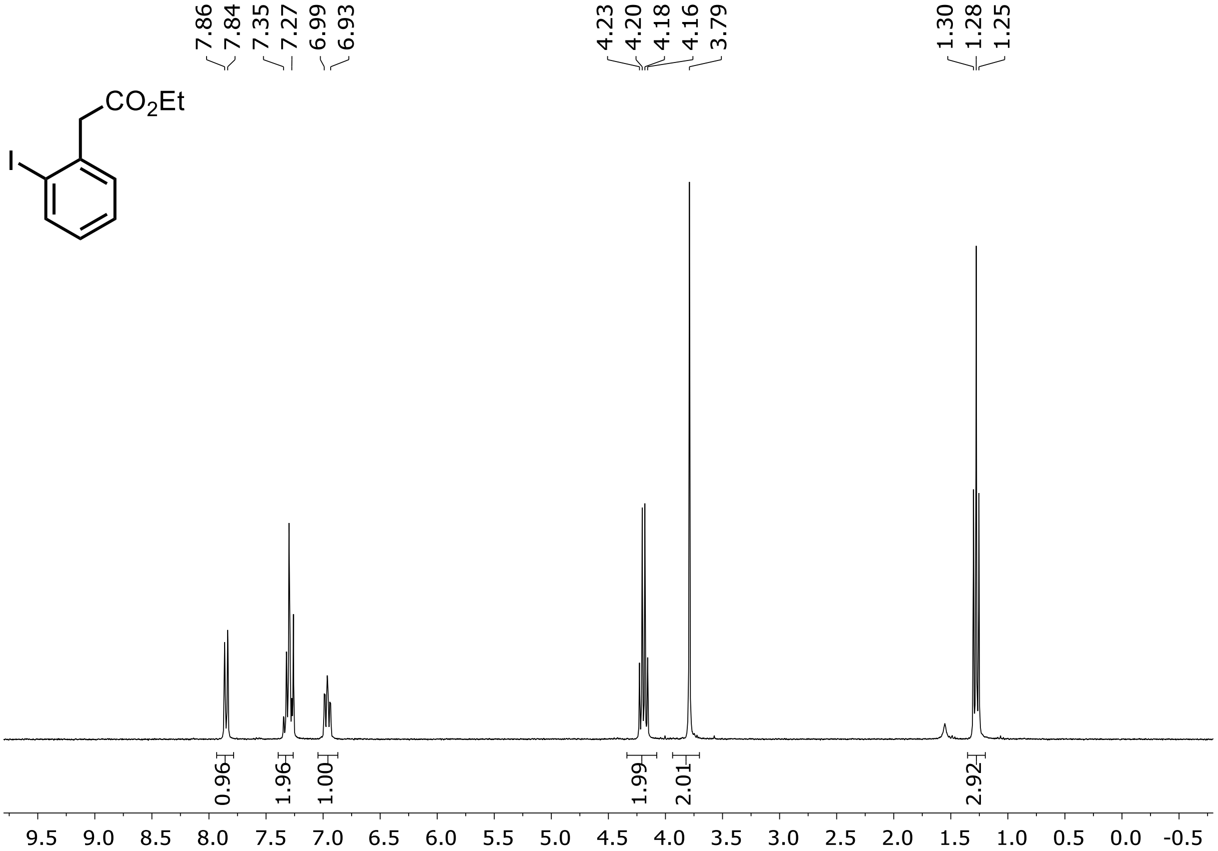

^{1}H NMR (300 MHz, $CDCl_3$) of ethyl (2-iodophenyl)acetate.

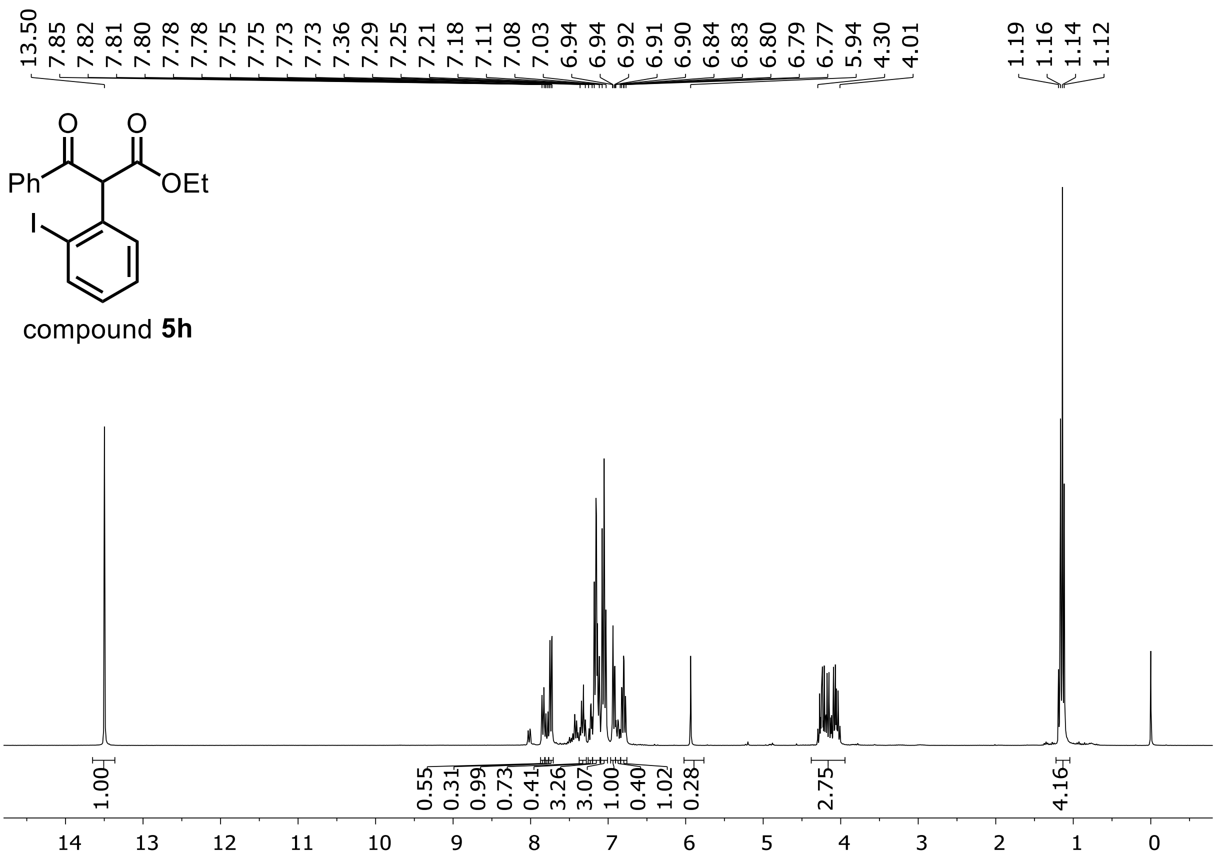

^{1}H NMR (300 MHz, $CDCl_3$) of compound **5h**.

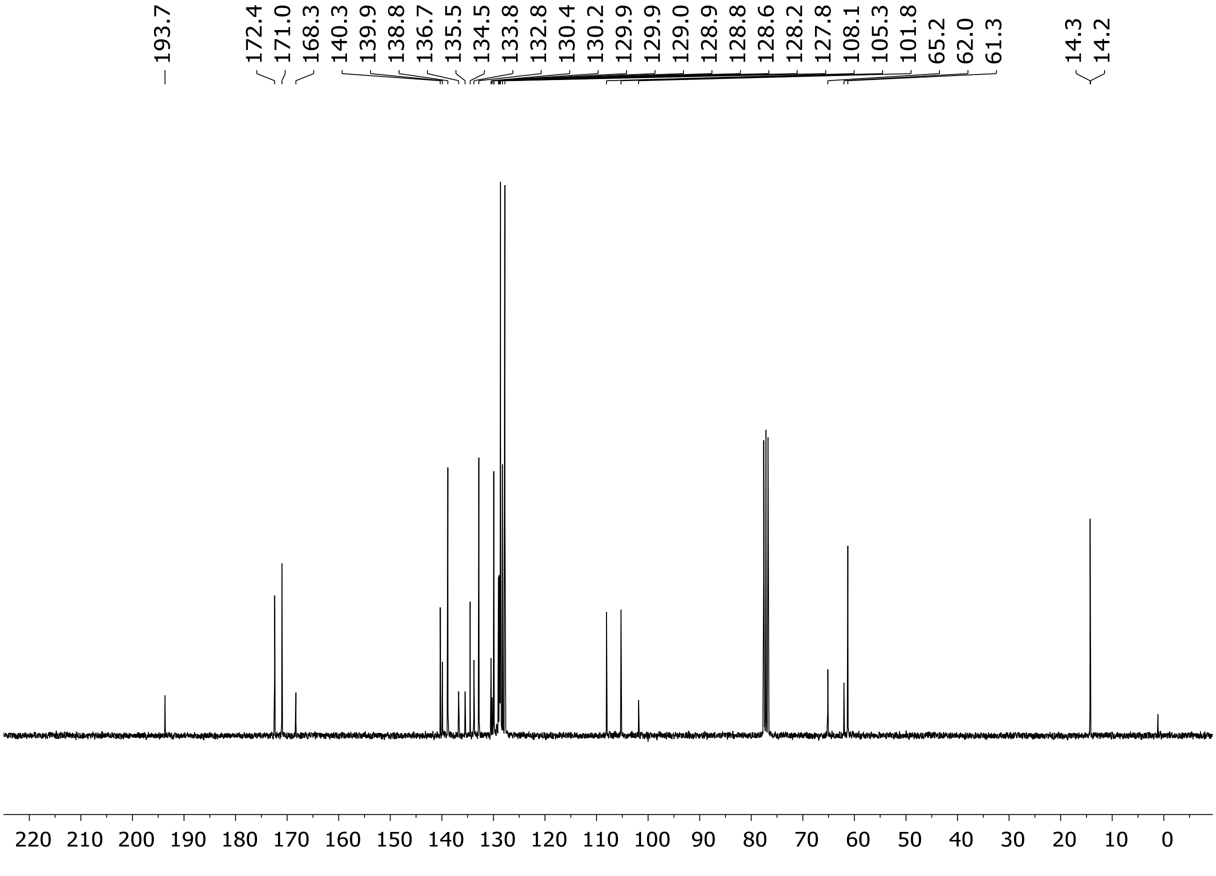

$^{13}C\{^{1}H\}$ NMR (75 MHz, $CDCl_3$) of compound **5h**.

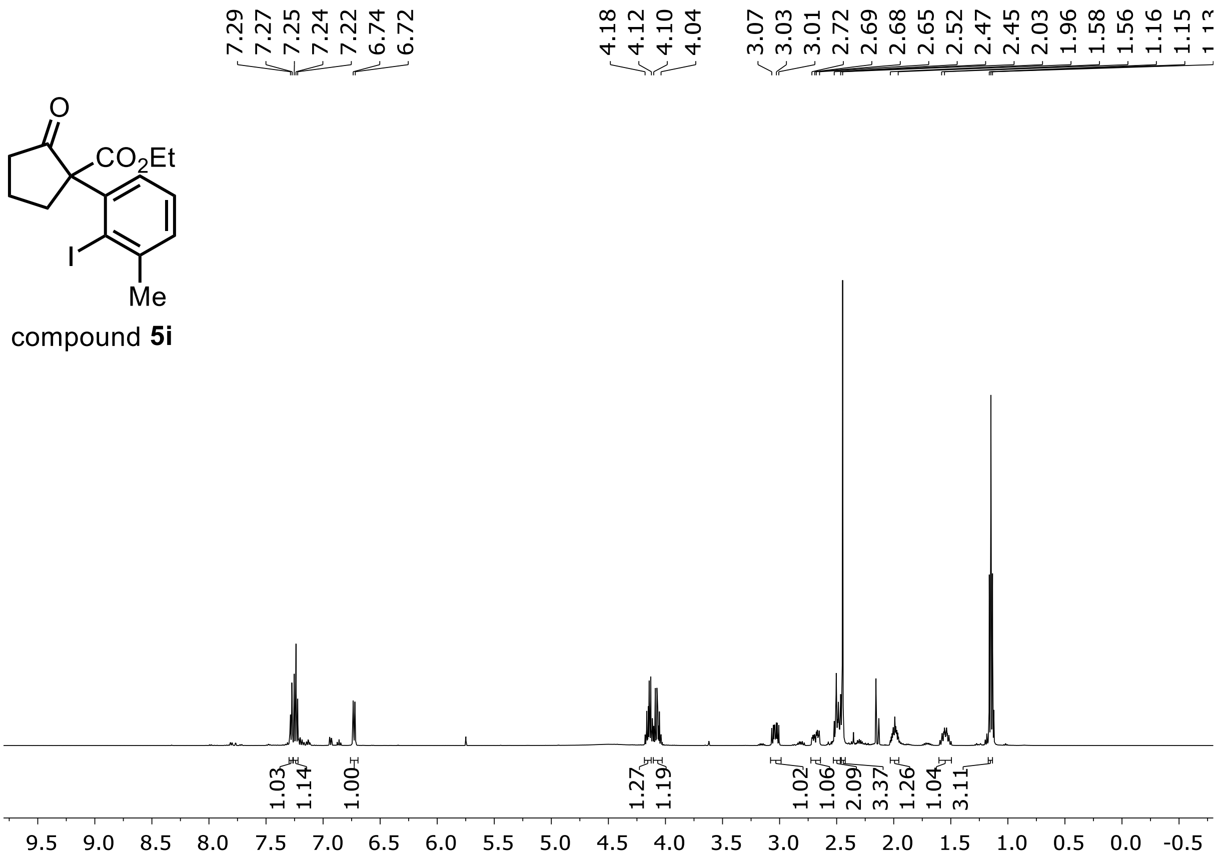

^{1}H NMR (500 MHz, DMSO-d_6) of compound **5i**.

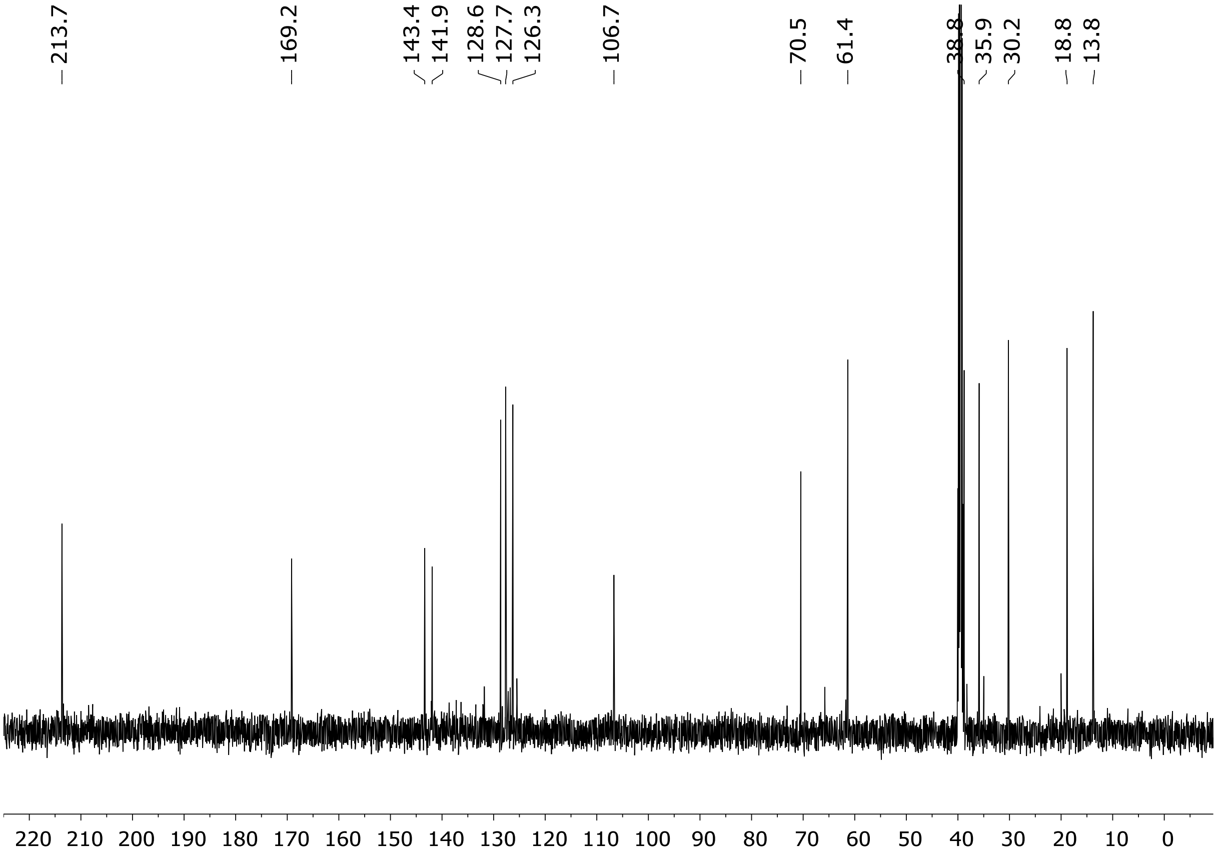

^{13}C{^{1}H} NMR (125 MHz, DMSO-d_6) of compound **5i**.

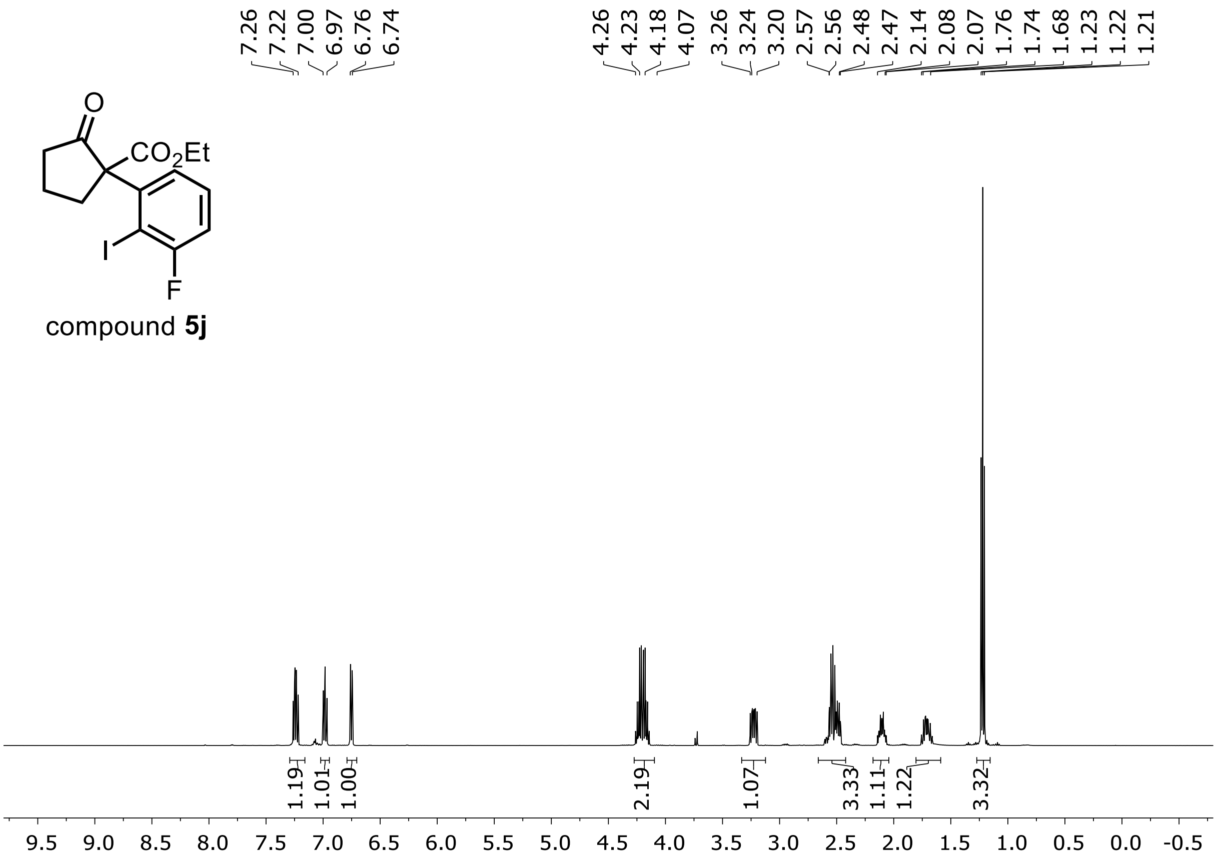

^{1}H NMR (500 MHz, $CDCl_3$) of compound **5j**.

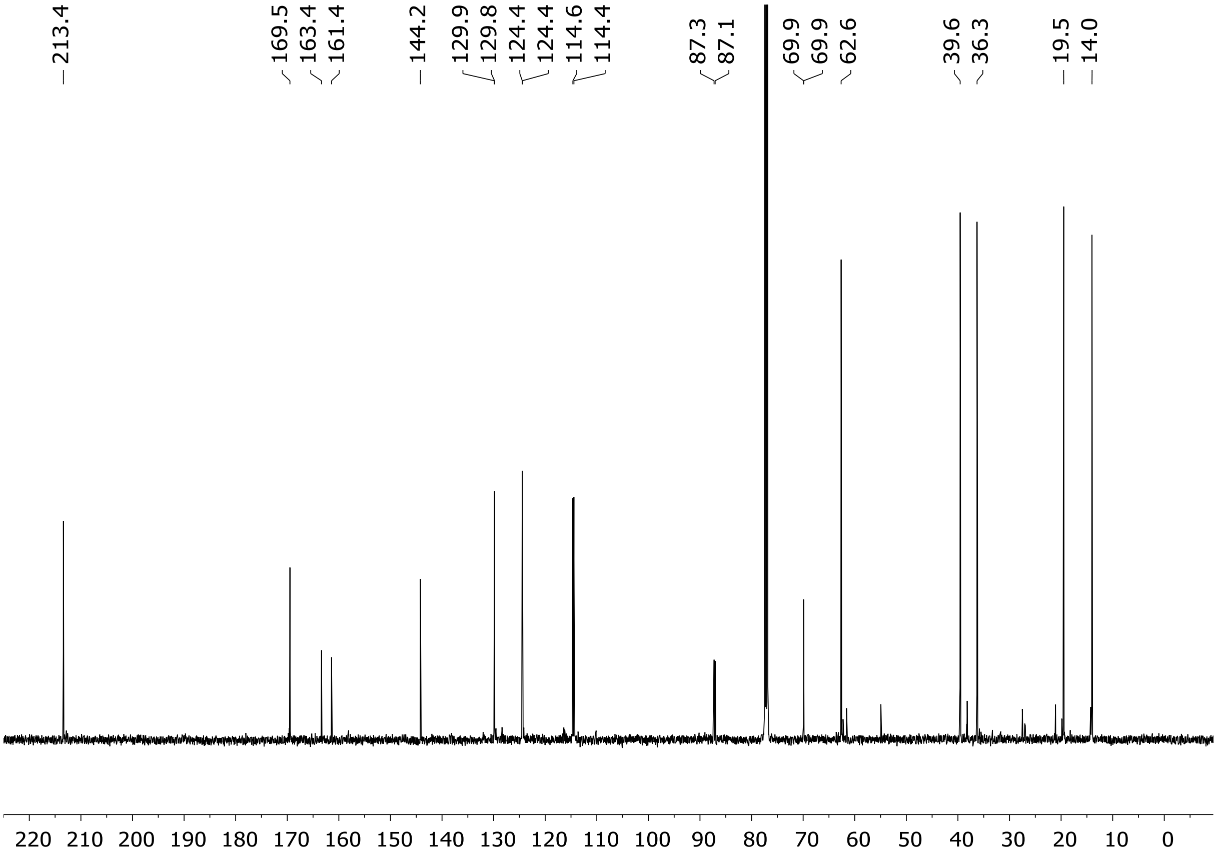

$^{13}C\{^{1}H\}$ NMR (125 MHz, $CDCl_3$) of compound **5j**.

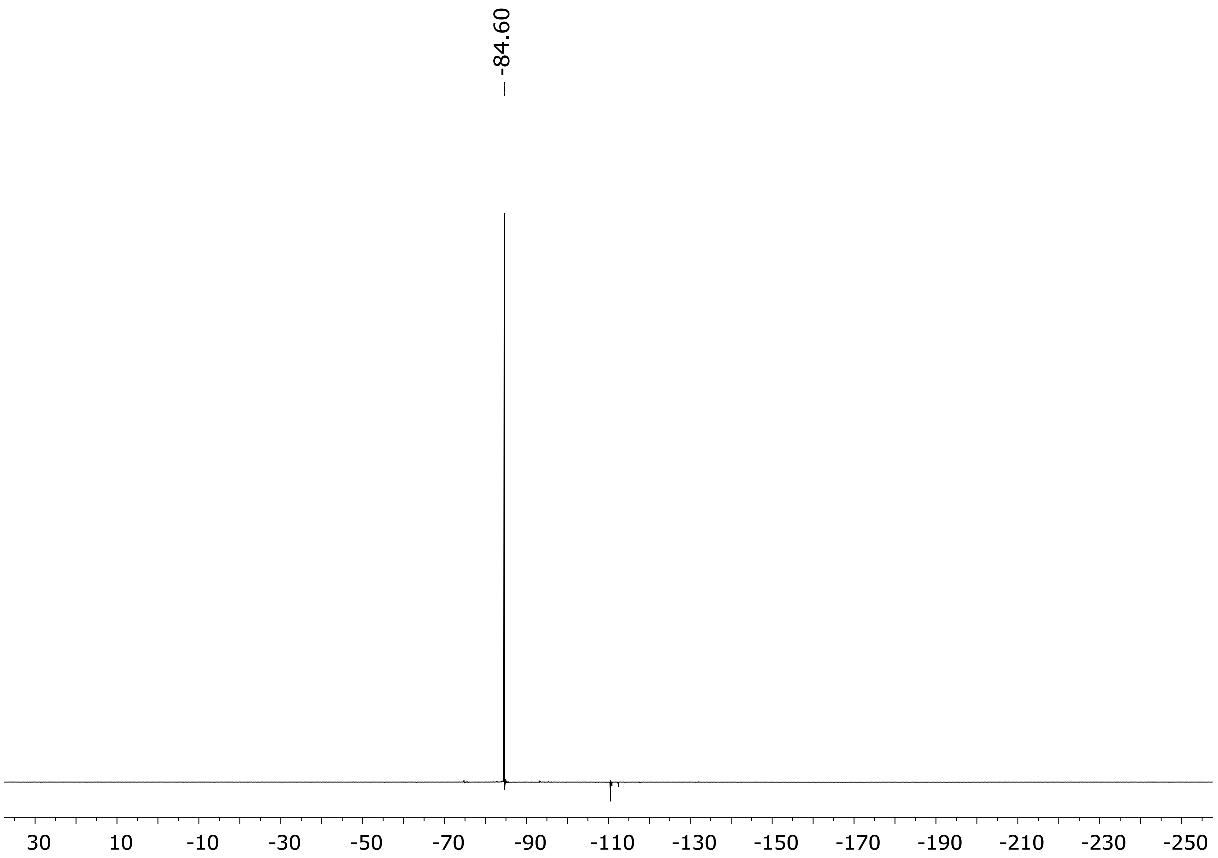

$^{19}F\{^{1}H\}$ NMR (470 MHz, $CDCl_3$) of compound **5j**.

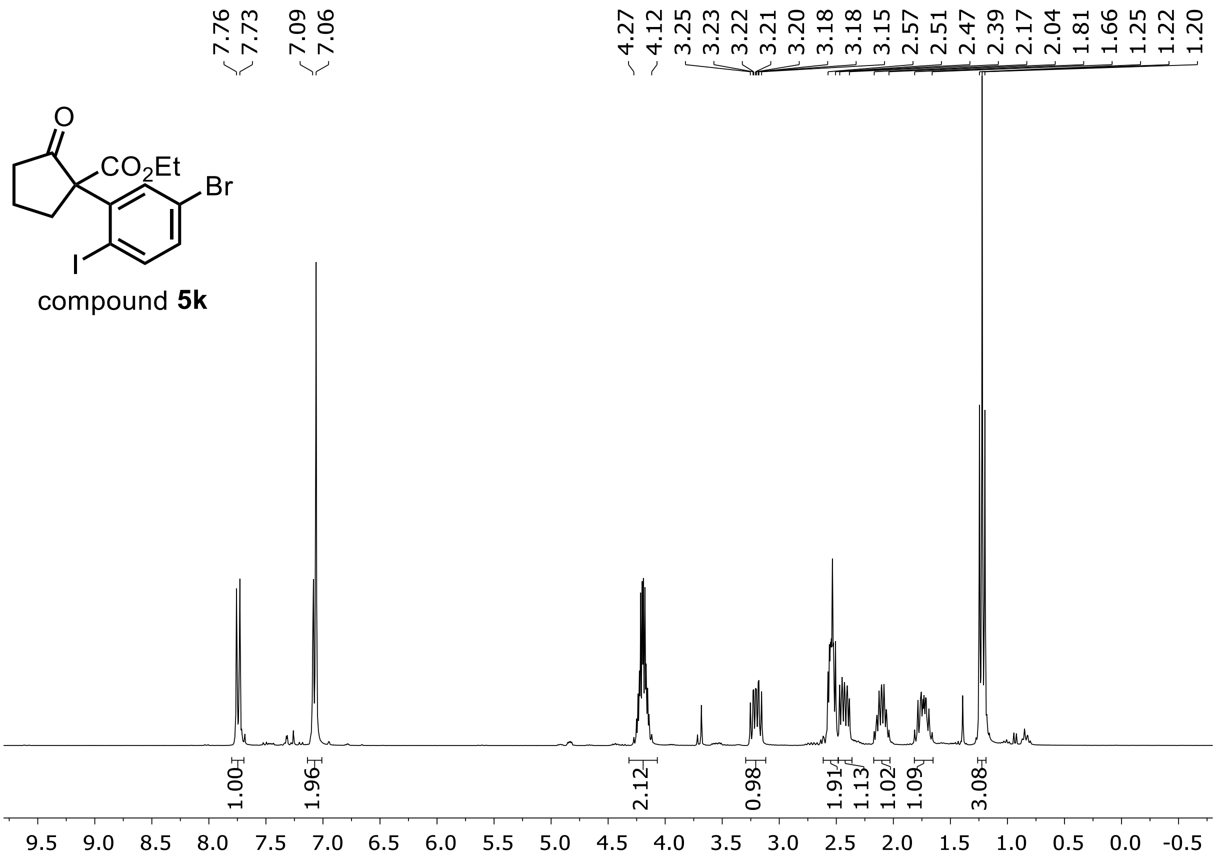

^{1}H NMR (300 MHz, $CDCl_3$) of compound **5k**.

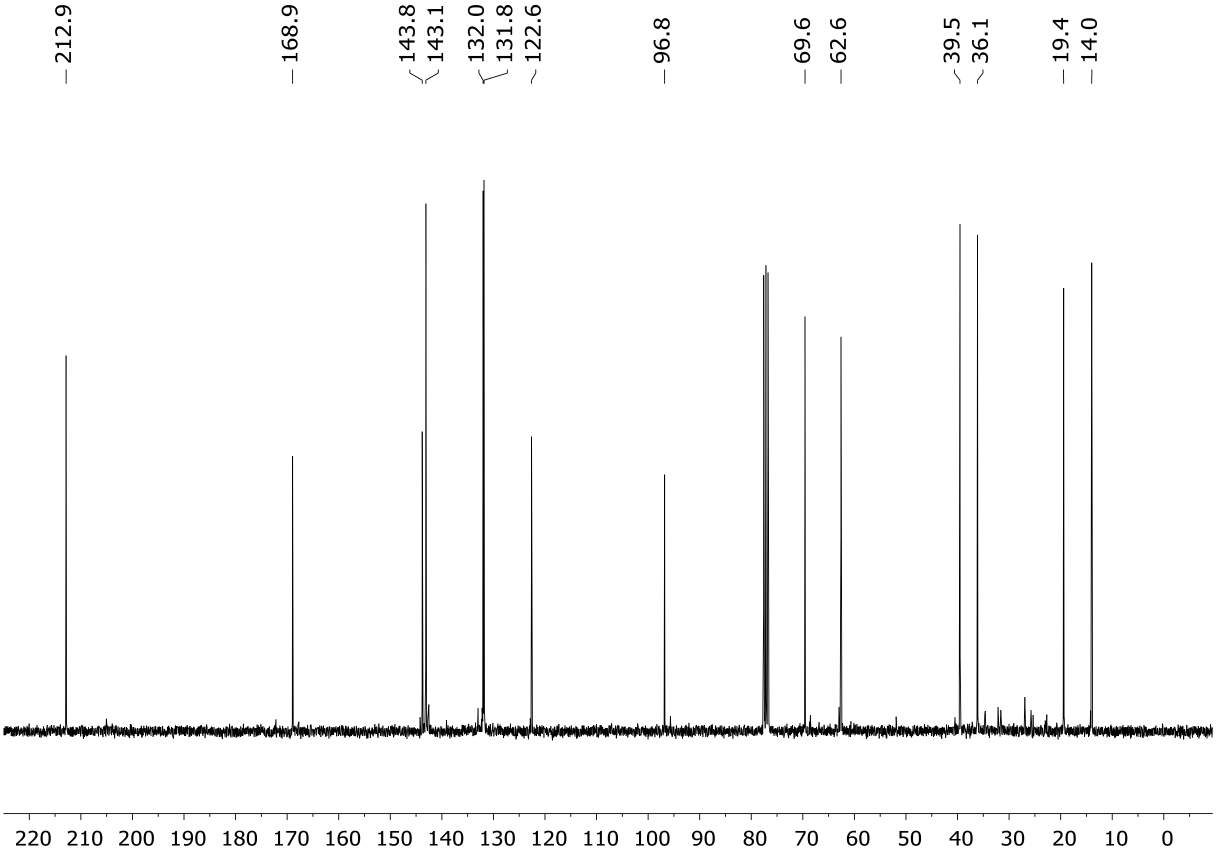

^{13}C{^{1}H} NMR (75 MHz, $CDCl_3$) of compound **5k**.

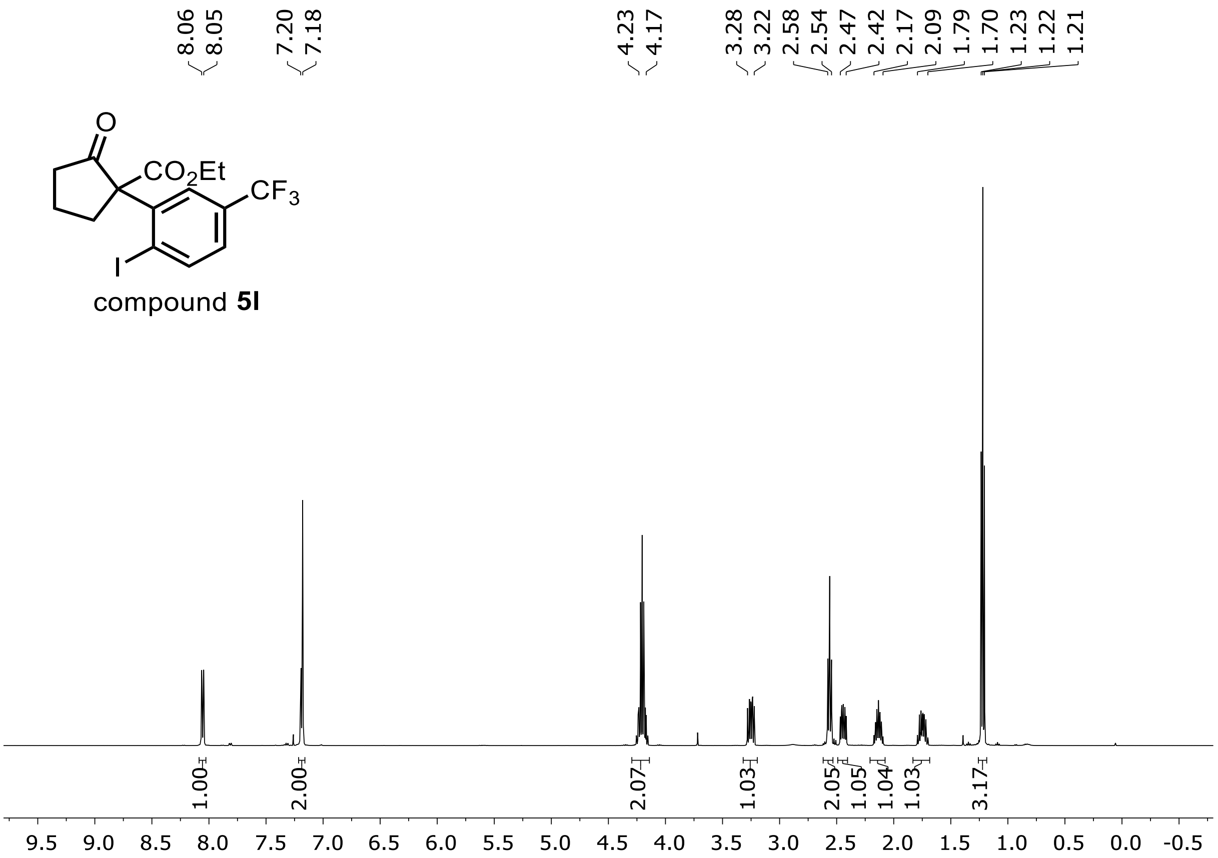

^{1}H NMR (500 MHz, $CDCl_3$) of compound **5l**.

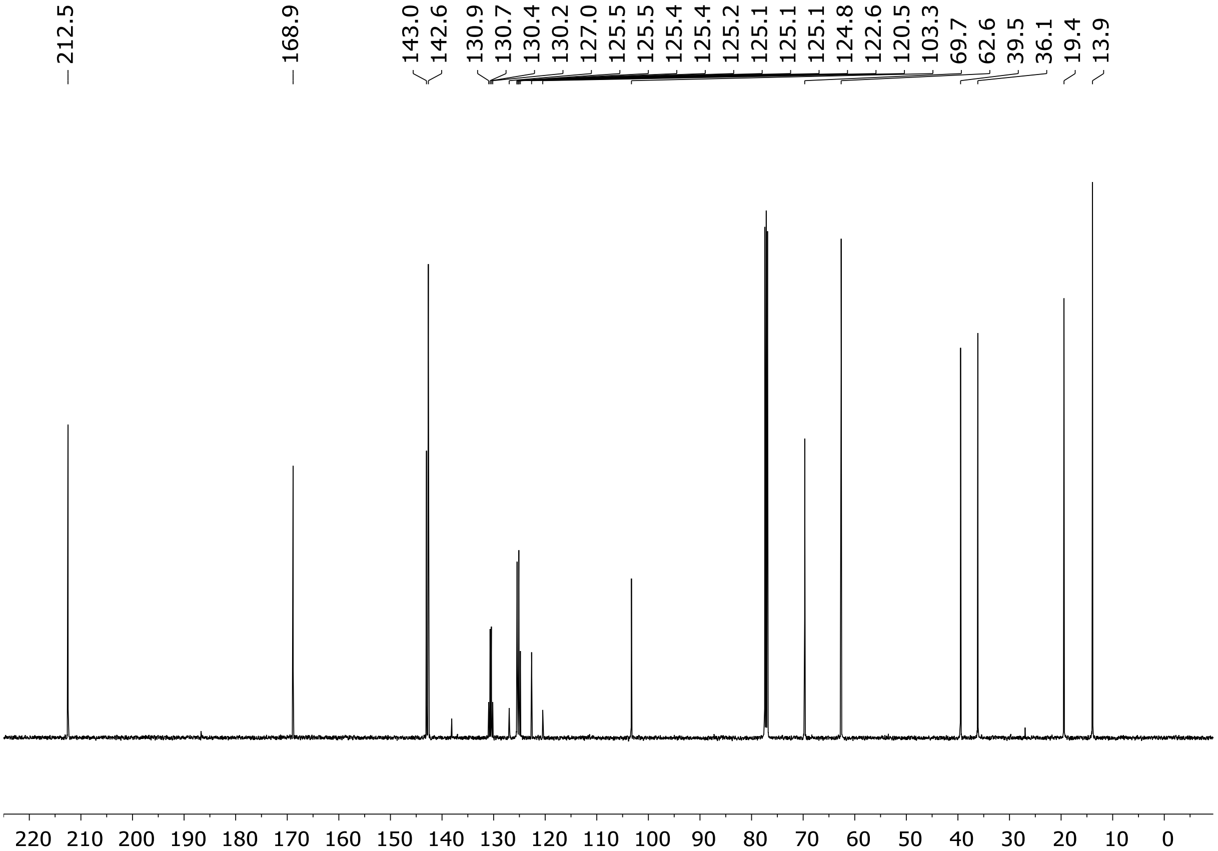

^{13}C{^{1}H} NMR (125 MHz, $CDCl_3$) of compound **5l**.

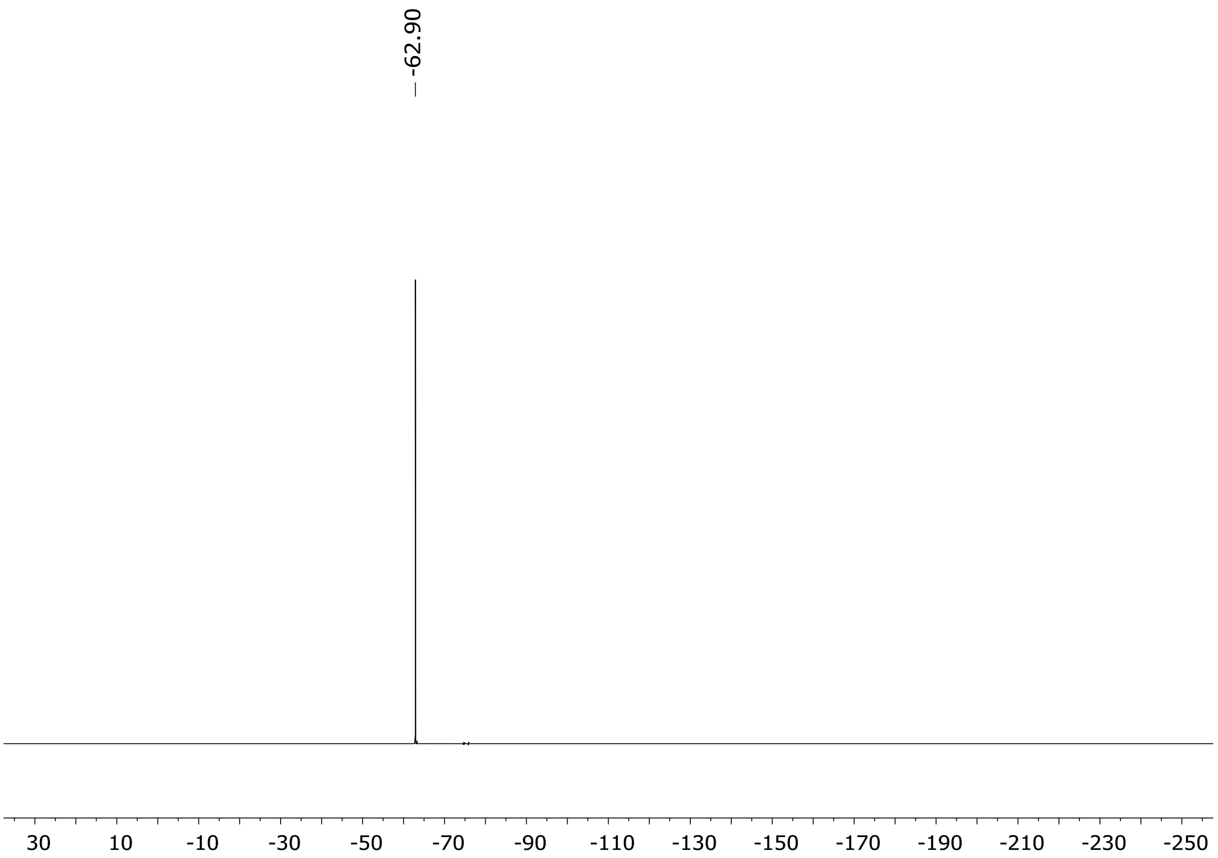

$^{19}F\{^1H\}$ NMR (470 MHz, $CDCl_3$) of compound **5l**.

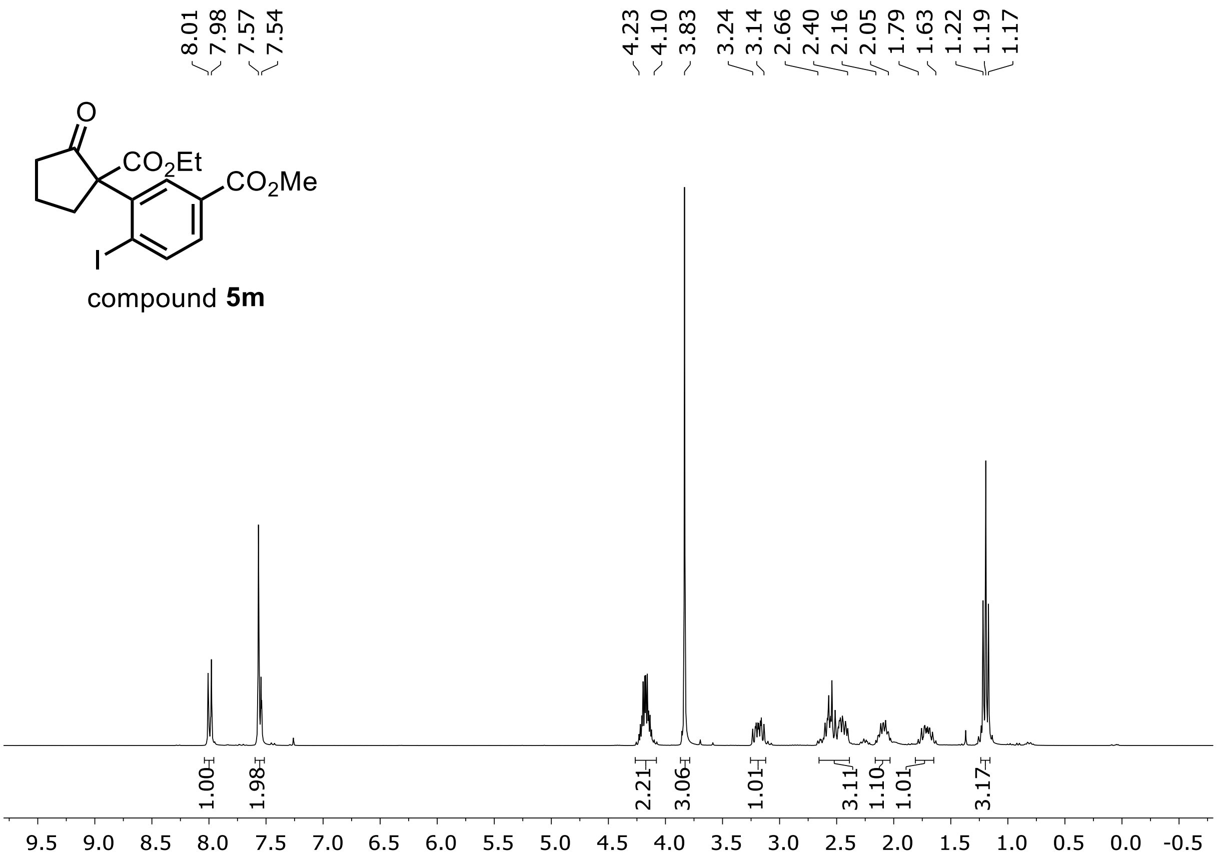

^{1}H NMR (300 MHz, $CDCl_3$) of compound **5m**.

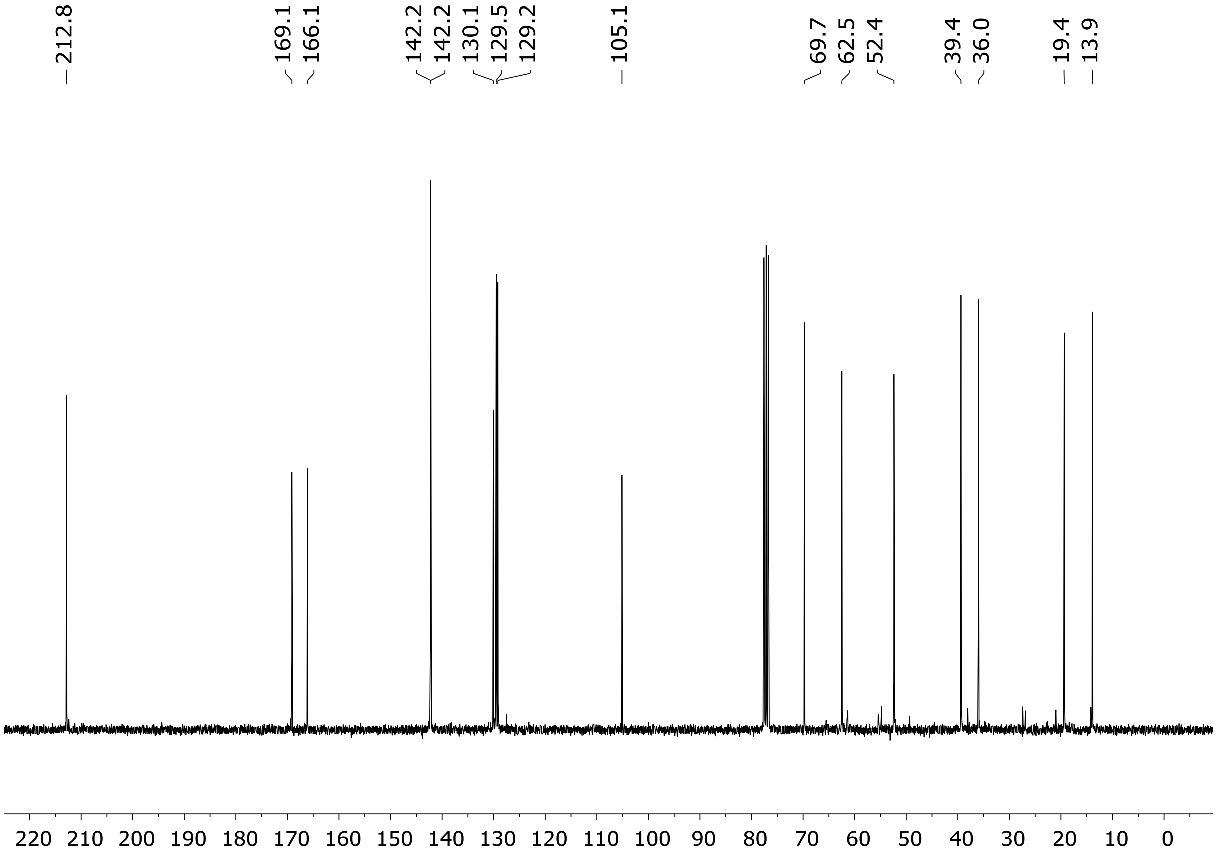

$^{13}C\{^{1}H\}$ NMR (75 MHz, $CDCl_3$) of compound **5m**.

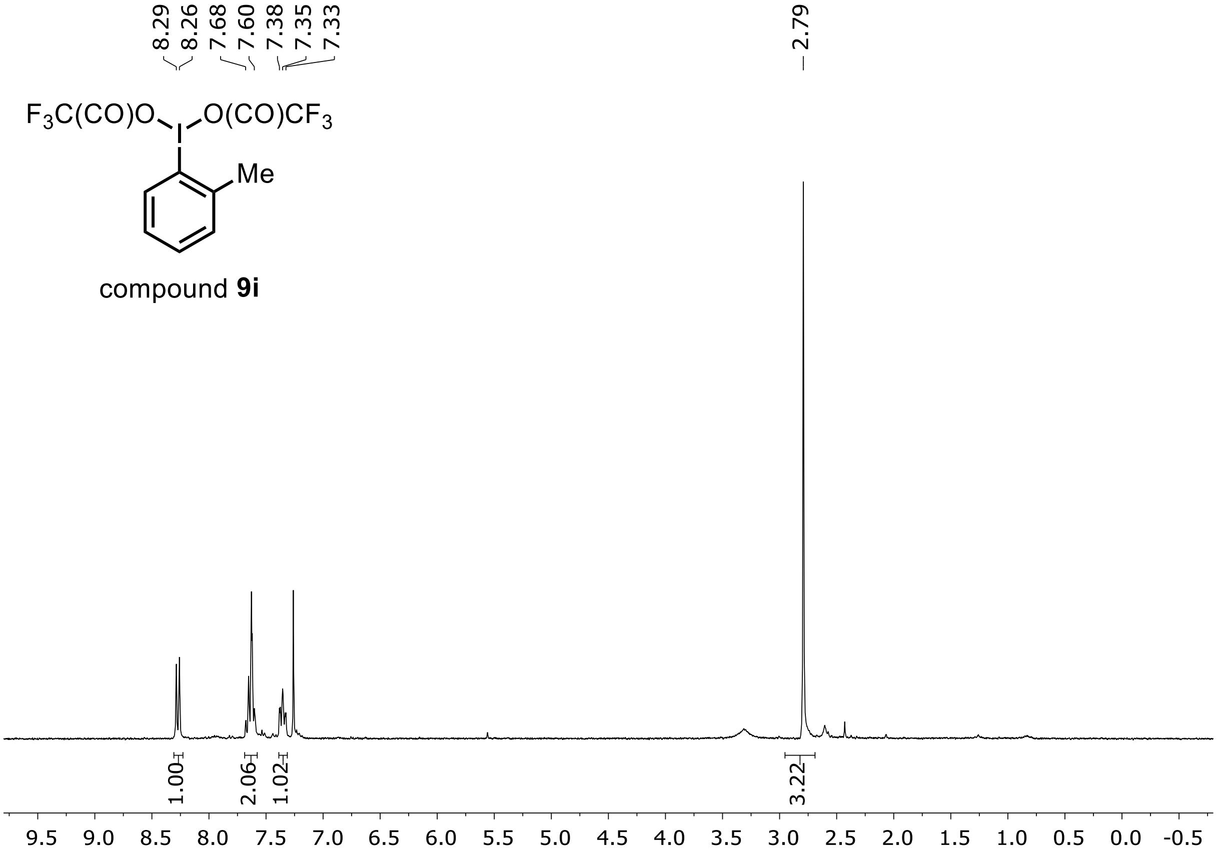

compound **9i**

^{1}H NMR (300 MHz, $CDCl_3$) of compound **9i**.

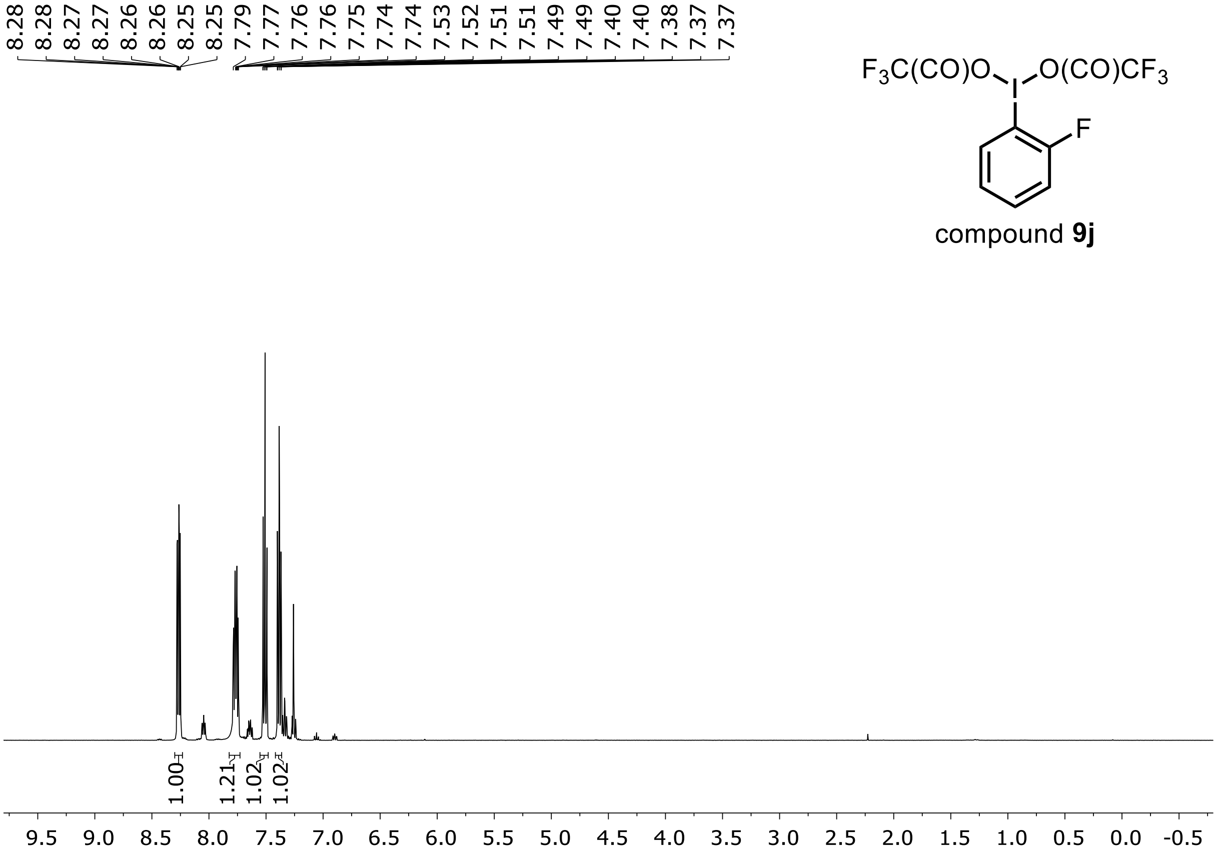

^{1}H NMR (500 MHz, $CDCl_3$) of compound **9j**.

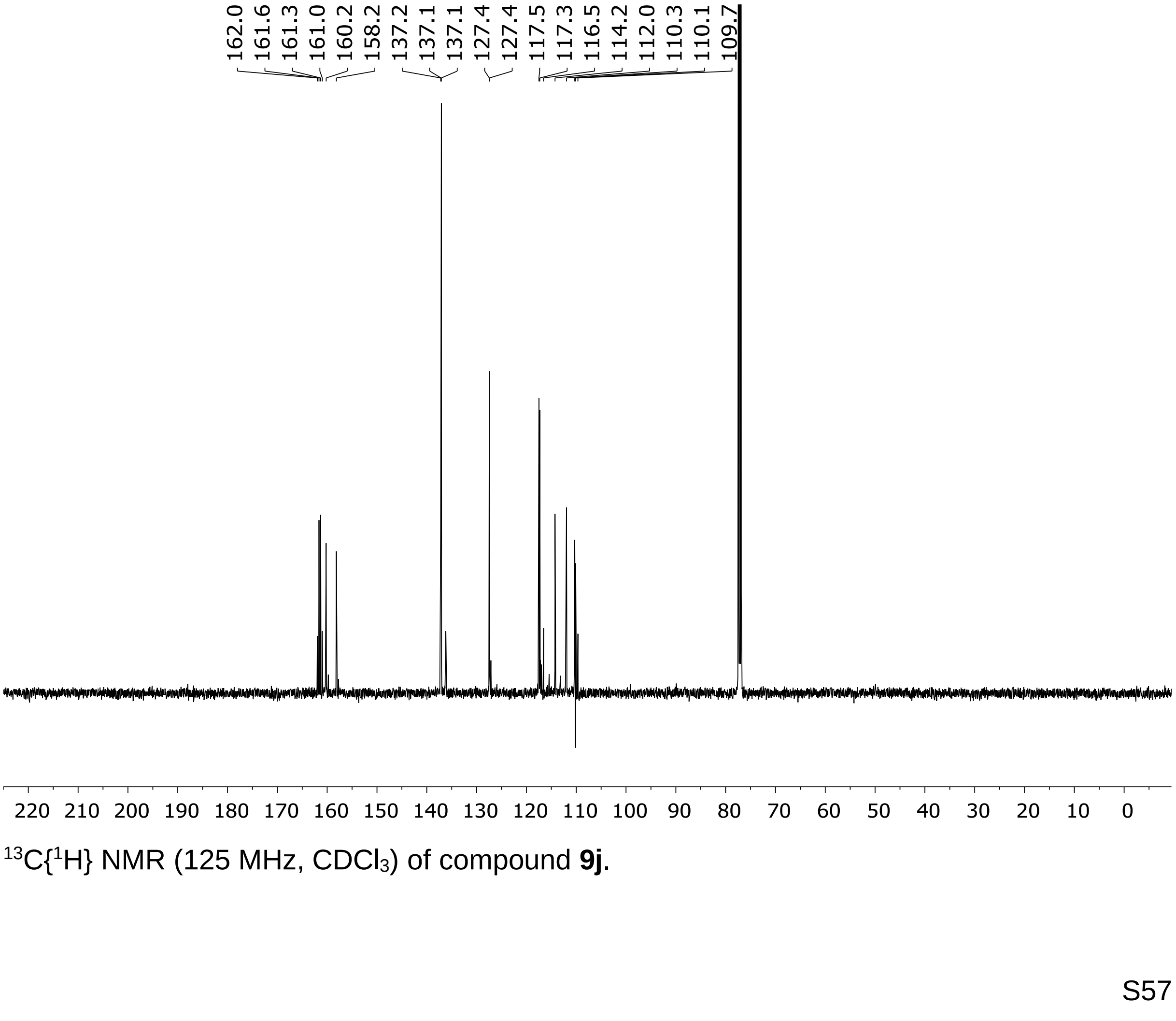

^{13}C{^{1}H} NMR (125 MHz, $CDCl_3$) of compound **9j**.

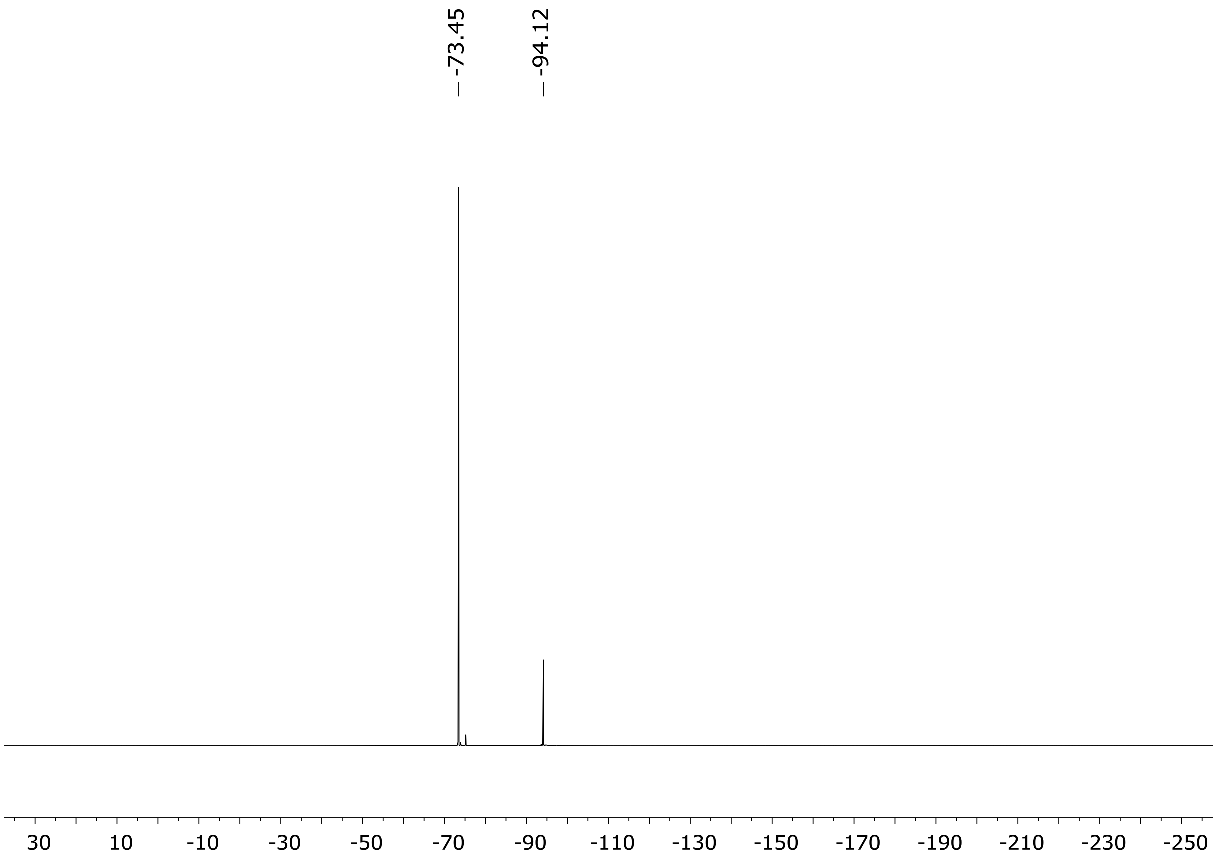

$^{19}F\{^1H\}$ NMR (470 MHz, $CDCl_3$) of compound **9j**.

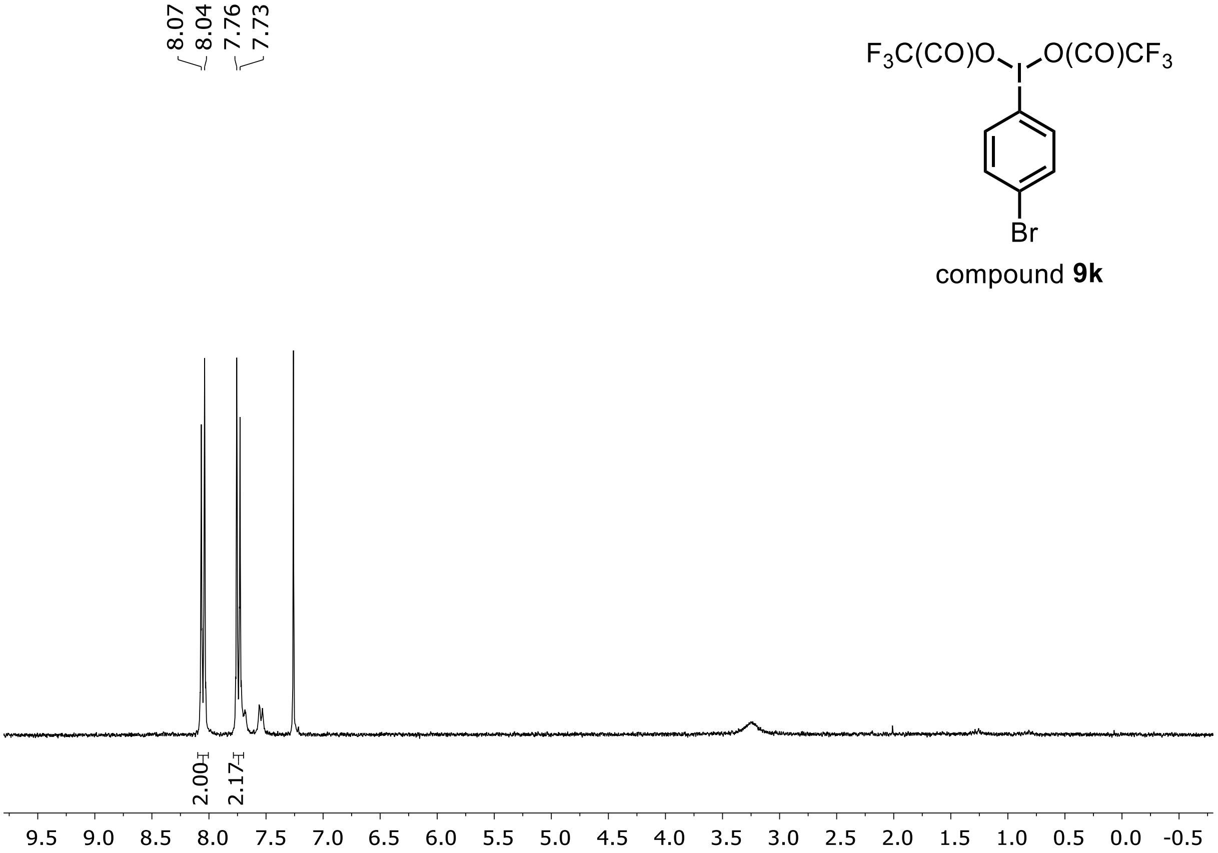

^{1}H NMR (300 MHz, $CDCl_3$) of compound **9k**.

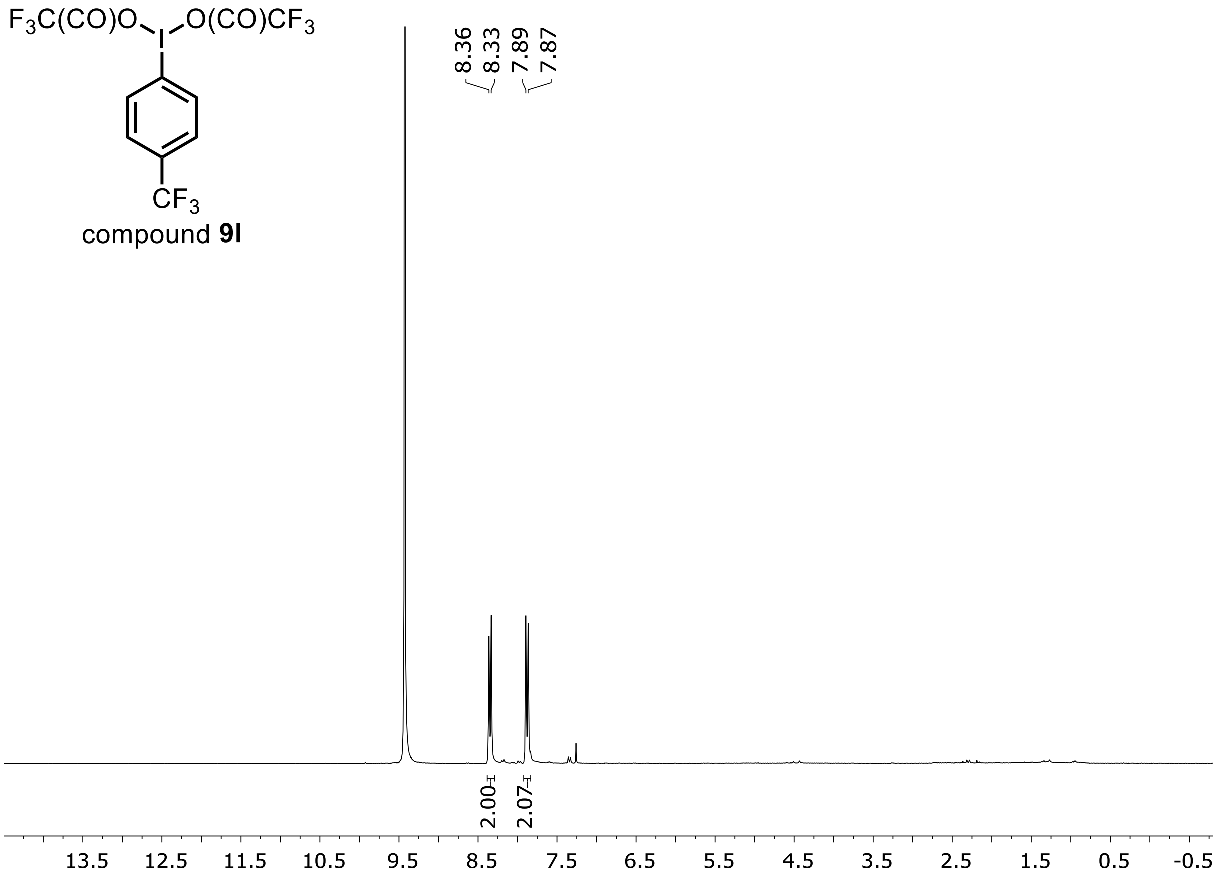

^{1}H NMR (300 MHz, $CDCl_3$ + 1 vol% TFA) of compound **9l**.

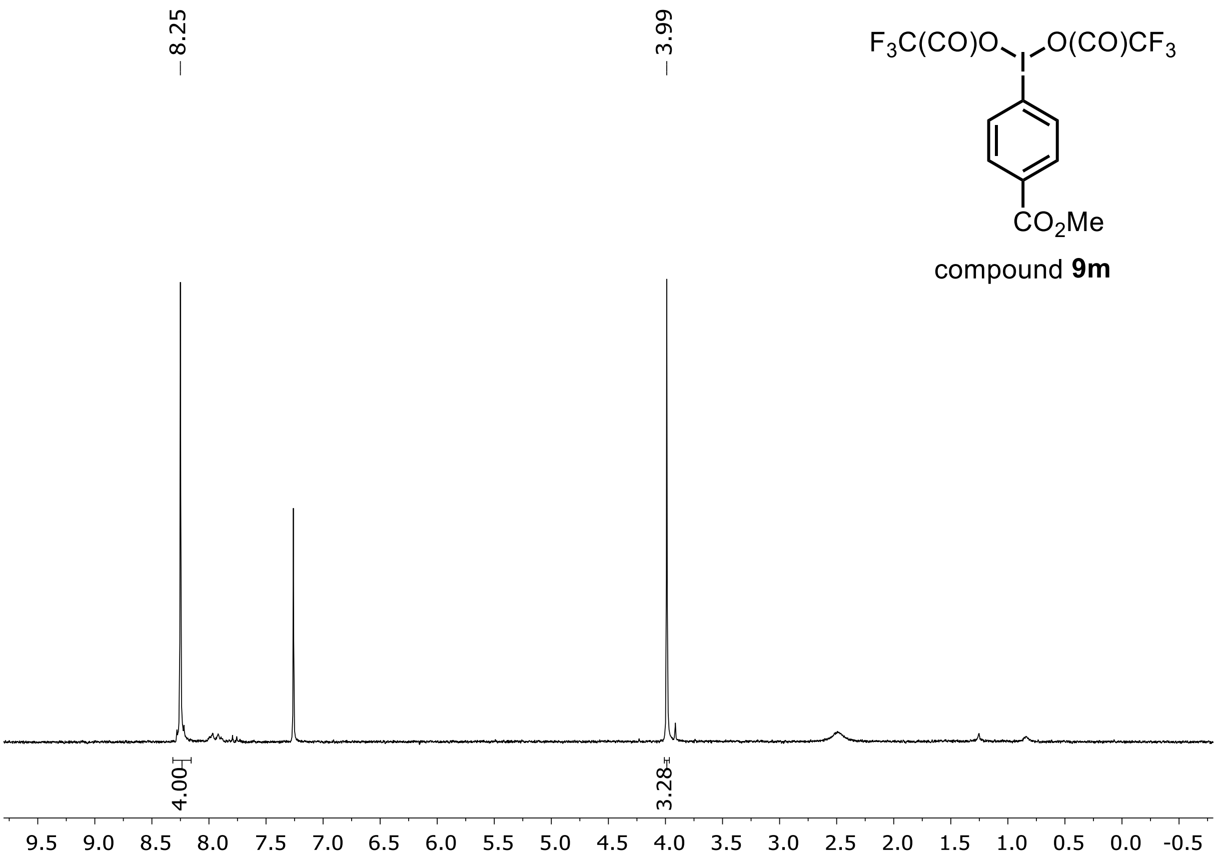

^1H NMR (300 MHz, $CDCl_3$) of compound **9m**.

8. GLC of Compounds 2a, 8 and 2c–2m

<Sample Information>

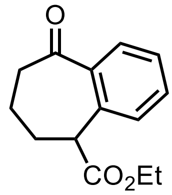

compound **2a**

Sample Name	: Compound_2a
Sample ID	: JUST-249
Data Filename	: Compound_2a.gcd
Method Filename	: 50J.gcm
Batch Filename	: Batch 1.gcb
Vial #	: 1
Injection Volume	: 1 uL
Date Acquired	: 02.12.2019 15:40:30
Date Processed	: 02.12.2019 17:52:26
Sample Type	: Unknown
Acquired by	: System Administrator
Processed by	: System Administrator

<Chromatogram>

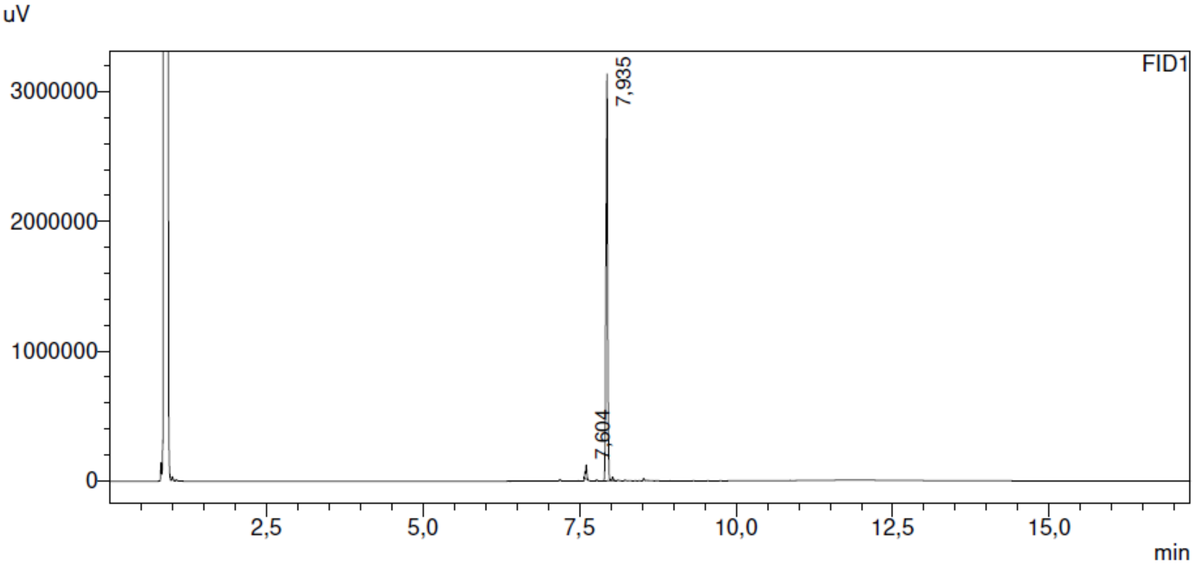

<Peak Table>

FID1

Peak#	Ret. Time	Area	Height	Conc.	Unit	Mark	Name
1	7,604	167257	104860	2,895		M	
2	7,935	5611077	3051080	97,105		M	
Total		5778335	3155940				

<Sample Information>

Sample Name : Compound_6b
Sample ID : JUST-234
Data Filename : Compound_6b.gcd
Method Filename : 50J.gcm
Batch Filename : Batch 1.gcb
Vial # : 2
Injection Volume : 1 uL
Date Acquired : 02.12.2019 16:05:19
Date Processed : 03.12.2019 10:09:08

Sample Type : Unknown

Acquired by : System Administrator
Processed by : System Administrator

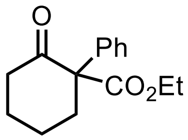

compound **8**

<Chromatogram>

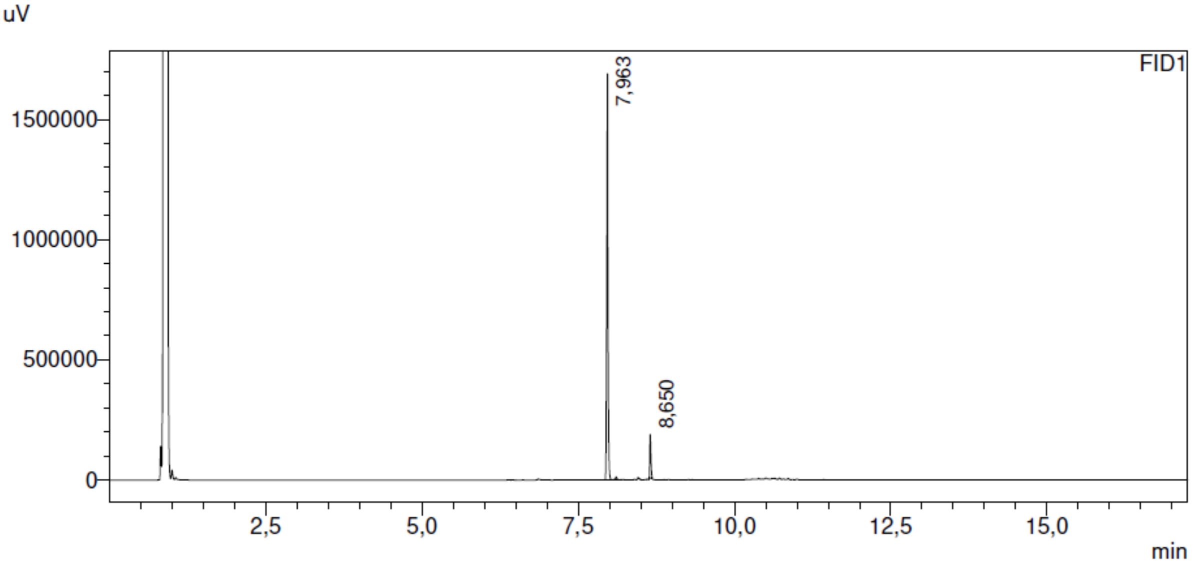

<Peak Table>

FID1

Peak#	Ret. Time	Area	Height	Conc.	Unit	Mark	Name
1	7,963	2484248	1624552	92,164		M	
2	8,650	211215	175139	7,836		M	
Total		2695463	1799691				

<Sample Information>

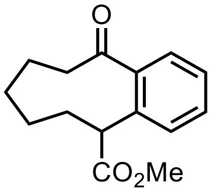

compound **2c**

Sample Name	: JUST-237-RG18		
Sample ID	: JUST-237		
Data Filename	: JUST-237-RG18.gcd		
Method Filename	: 50J.gcm		
Batch Filename	: Batch 1.gcb		
Vial #	: 3	Sample Type	: Unknown
Injection Volume	: 1 uL		
Date Acquired	: 04.12.2019 16:35:23	Acquired by	: System Administrator
Date Processed	: 04.12.2019 18:21:35	Processed by	: System Administrator

<Chromatogram>

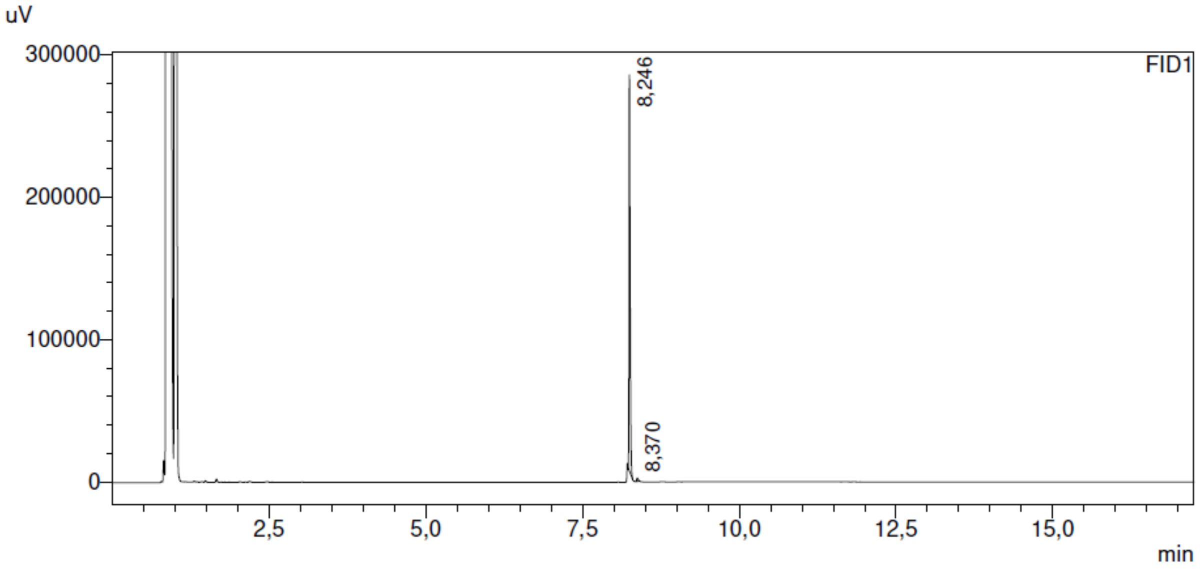

<Peak Table>

FID1

Peak#	Ret. Time	Area	Height	Conc.	Unit	Mark	Name
1	8,246	341954	269429	98,866		M	
2	8,370	3923	2274	1,134		M	
Total		345877	271702				

<Sample Information>

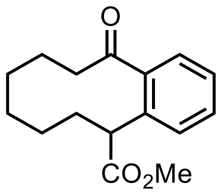

compound **2d**

Sample Name : Compound_2d
Sample ID : JUST-289
Data Filename : Compound-2d.gcd
Method Filename : 50J.gcm
Batch Filename : Batch 1.gcb
Vial # : 4
Injection Volume : 1 uL
Date Acquired : 02.12.2019 19:48:24
Date Processed : 03.12.2019 10:07:17

Sample Type : Unknown
Acquired by : System Administrator
Processed by : System Administrator

<Chromatogram>

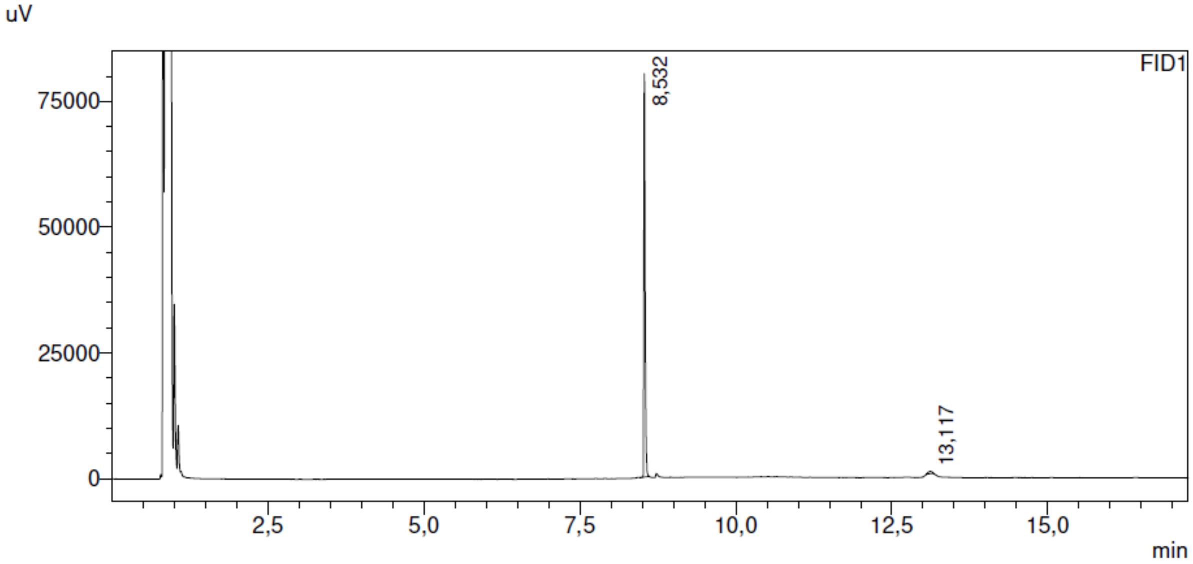

<Peak Table>

FID1

Peak#	Ret. Time	Area	Height	Conc.	Unit	Mark	Name
1	8,532	108251	76567	98,275		M	
2	13,117	1900	429	1,725		M	
Total		110151	76997				

<Sample Information>

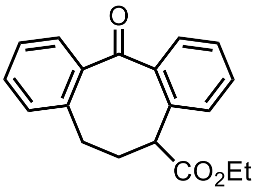

compound **2f**

Sample Name : JUST-253-RG10
Sample ID : JUST-253
Data Filename : JUST-253-RG10.gcd
Method Filename : 50J.gcm
Batch Filename : Batch 1.gcb
Vial # : 1
Injection Volume : 1 uL
Date Acquired : 04.12.2019 17:37:58
Date Processed : 05.12.2019 11:59:04

Sample Type : Unknown
Acquired by : System Administrator
Processed by : System Administrator

<Chromatogram>

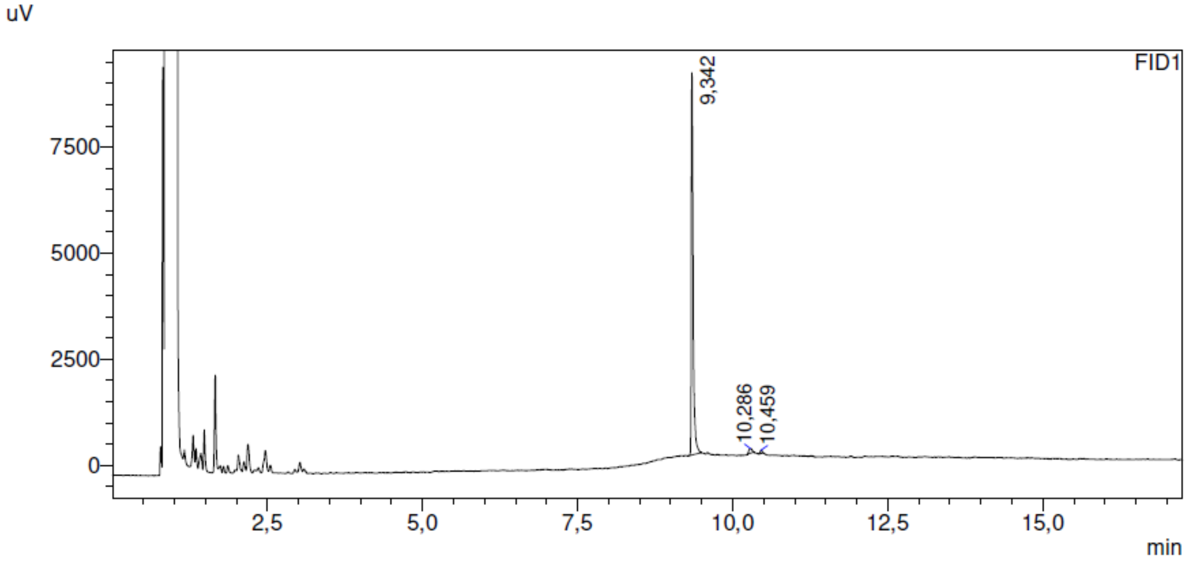

<Peak Table>

FID1

Peak#	Ret. Time	Area	Height	Conc.	Unit	Mark	Name
1	9,342	18493	8925	97,024		M	
2	10,286	401	110	2,106		M	
3	10,459	166	78	0,870		M	
Total		19060	9112				

<Sample Information>

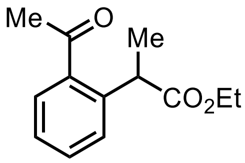

Sample Name	: Compound_2g		
Sample ID	: JUST-252		
Data Filename	: Compound_2g.gcd		
Method Filename	: 50J.gcm		
Batch Filename	: Batch 1.gcb		
Vial #	: 1	Sample Type	: Unknown
Injection Volume	: 1 uL		
Date Acquired	: 02.12.2019 18:33:03	Acquired by	: System Administrator
Date Processed	: 03.12.2019 10:02:21	Processed by	: System Administrator

compound **2g**

<Chromatogram>

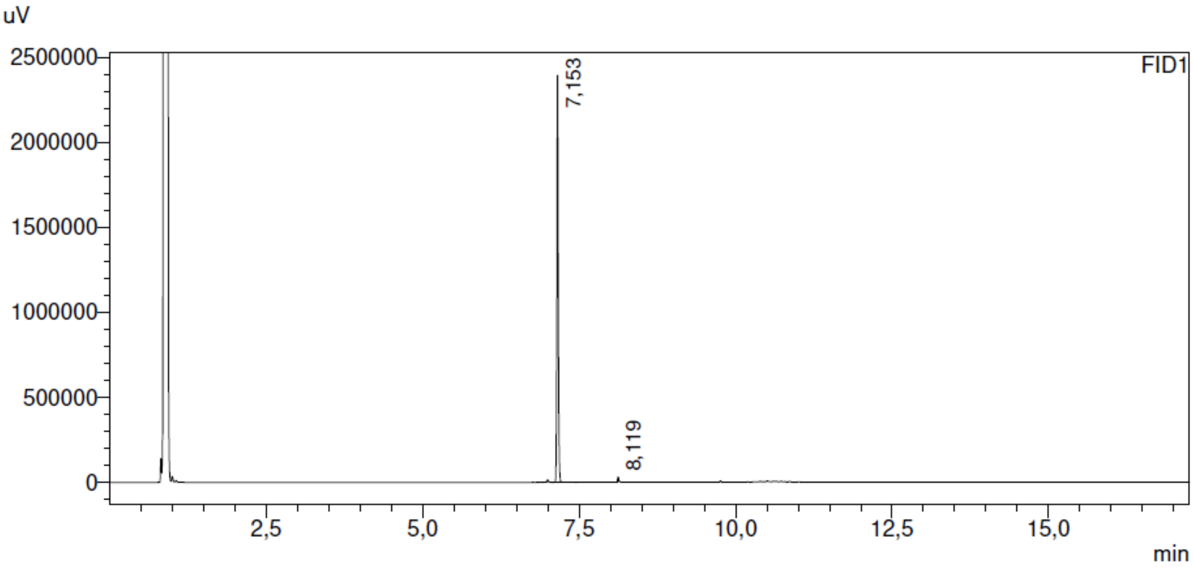

<Peak Table>

FID1

Peak#	Ret. Time	Area	Height	Conc.	Unit	Mark	Name
1	7,153	3690295	2342168	99,188		M	
2	8,119	30216	27274	0,812		M	
Total		3720510	2369441				

<Sample Information>

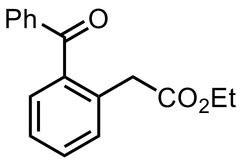

compound **2h**

Sample Name : Compound_2h_b
Sample ID : JUST-288
Data Filename : Compound-2h_b.gcd
Method Filename : 50J.gcm
Batch Filename : Batch 1.gcb
Vial # : 5
Injection Volume : 1 uL
Date Acquired : 02.12.2019 20:13:16
Date Processed : 03.12.2019 10:05:29

Sample Type : Unknown
Acquired by : System Administrator
Processed by : System Administrator

<Chromatogram>

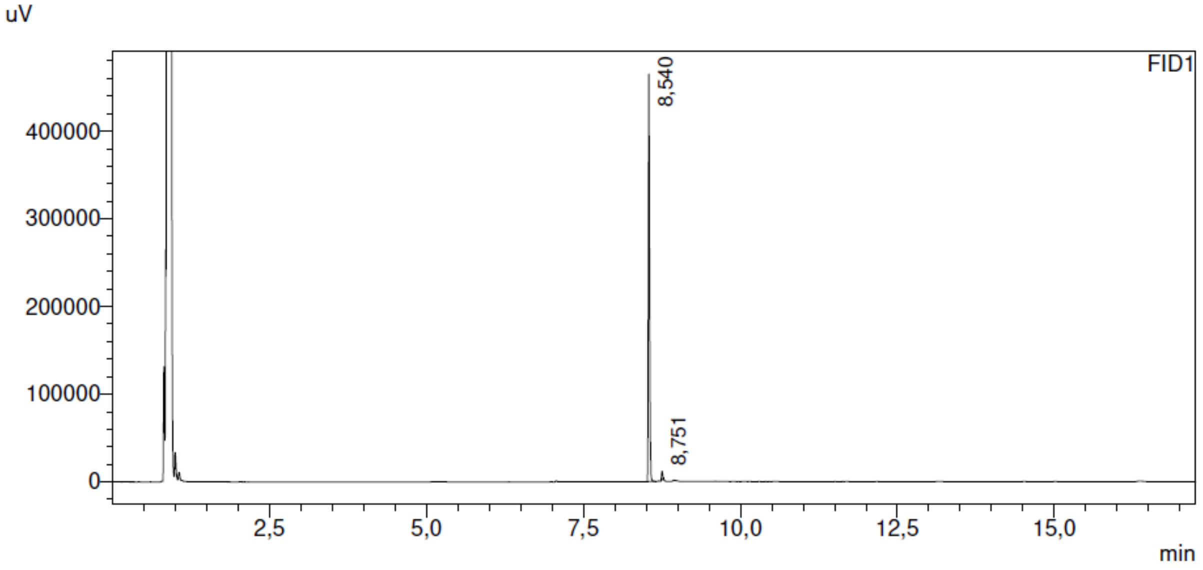

<Peak Table>

FID1

Peak#	Ret. Time	Area	Height	Conc.	Unit	Mark	Name
1	8,540	597859	451529	98,087		M	
2	8,751	11661	9215	1,913		M	
Total		609520	460744				

<Sample Information>

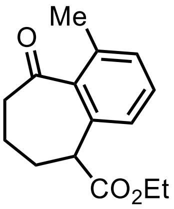

compound **2i**

Sample Name	: Compound_2i
Sample ID	: JUST-282
Data Filename	: Compound_2i.gcd
Method Filename	: 50J.gcm
Batch Filename	: Batch 1.gcb
Vial #	: 1
Injection Volume	: 1 uL
Date Acquired	: 03.12.2019 10:03:00
Date Processed	: 03.12.2019 15:32:49
Sample Type	: Unknown
Acquired by	: System Administrator
Processed by	: System Administrator

<Chromatogram>

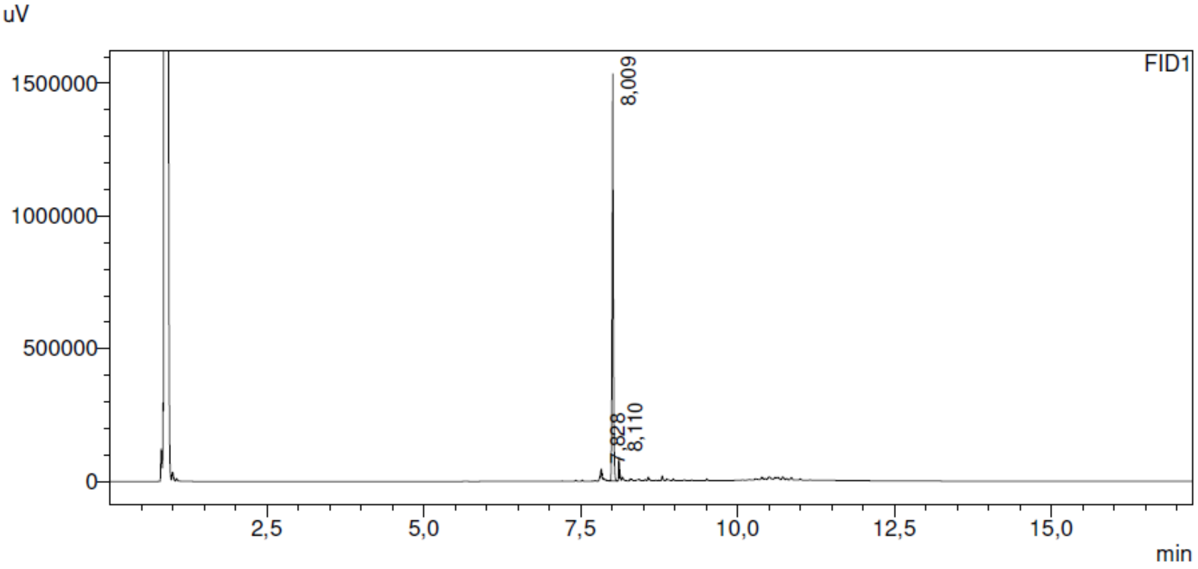

<Peak Table>

FID1

Peak#	Ret. Time	Area	Height	Conc.	Unit	Mark	Name
1	7,828	30027	23257	1,346		M	
2	8,009	2149537	1452914	96,323		M	
3	8,110	52035	60081	2,332		M	
Total		2231600	1536252				

<Sample Information>

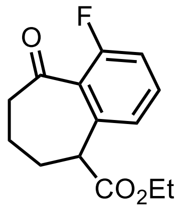

compound **2j**

Sample Name : Compound_2j_b
Sample ID : JUST-282
Data Filename : Compound_2j_b.gcd
Method Filename : 50J.gcm
Batch Filename : Batch 1.gcb
Vial # : 3
Injection Volume : 1 uL
Date Acquired : 03.12.2019 10:52:34
Date Processed : 03.12.2019 16:27:16

Sample Type : Unknown
Acquired by : System Administrator
Processed by : System Administrator

<Chromatogram>

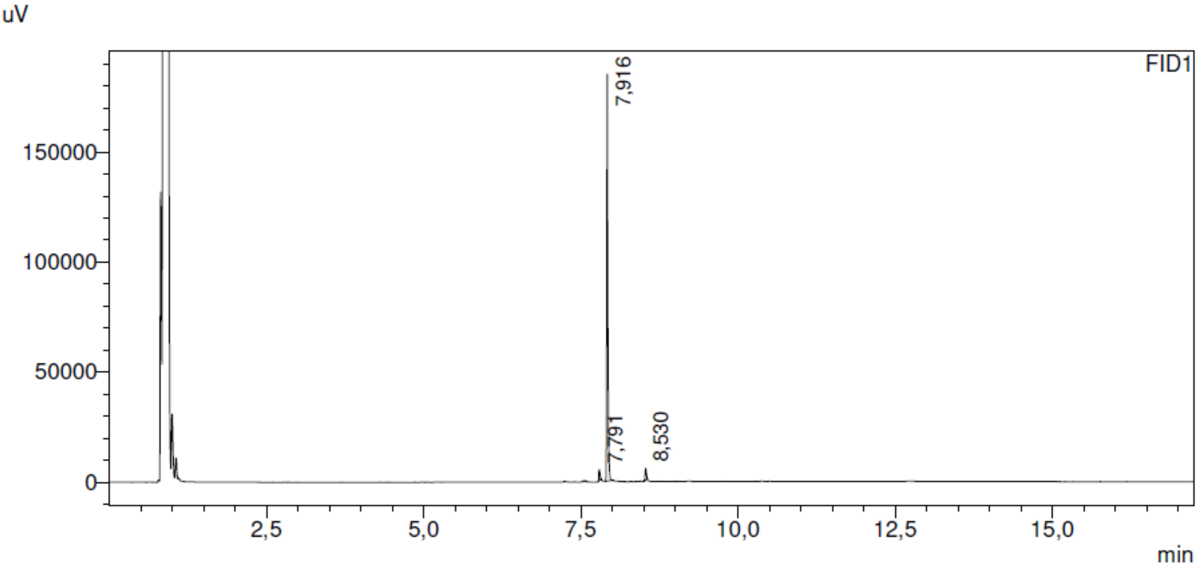

<Peak Table>

FID1

Peak#	Ret. Time	Area	Height	Conc.	Unit	Mark	Name
1	7,791	6331	4839	2,516		M	
2	7,916	238708	178393	94,863		M	
3	8,530	6595	5236	2,621		M	
Total		251635	188468				

<Sample Information>

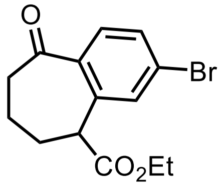

compound **2k**

Sample Name	: JUST-375-34-neu
Sample ID	:
Data Filename	: JUST-375-34-neu.gcd
Method Filename	: 50J.gcm
Batch Filename	: Batch 1.gcb
Vial #	: 22
Injection Volume	: 1 uL
Date Acquired	: 09.12.2019 12:06:59
Date Processed	: 09.12.2019 12:54:06
Sample Type	: Unknown
Acquired by	: System Administrator
Processed by	: System Administrator

<Chromatogram>

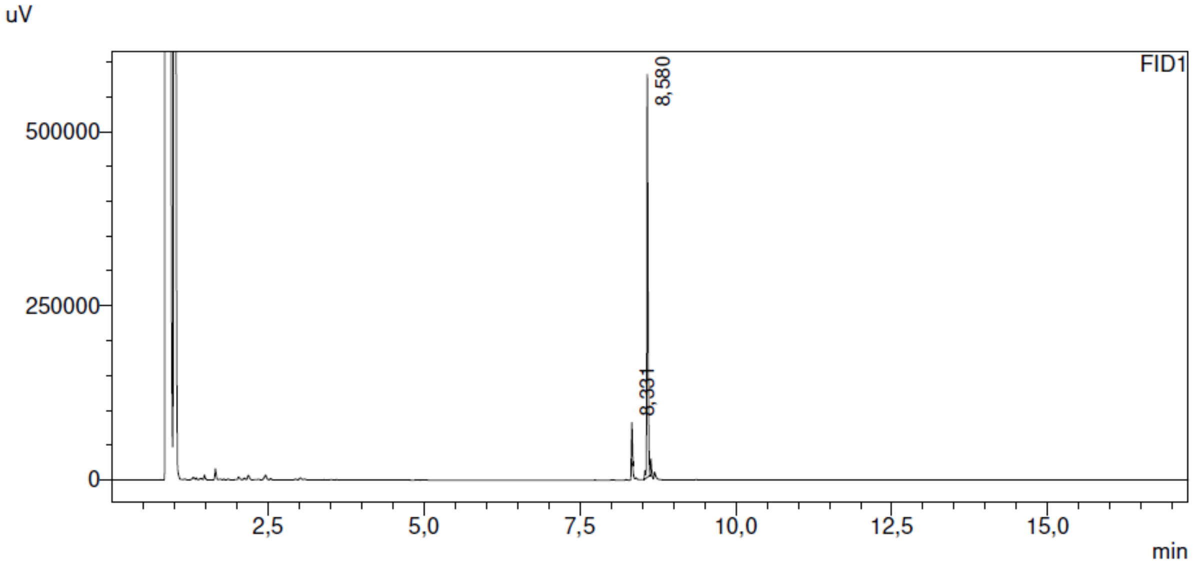

<Peak Table>

FID1

Peak#	Ret. Time	Area	Height	Conc.	Unit	Mark	Name
1	8,331	56265	56686	6,884		M	
2	8,580	761071	569087	93,116		M	
Total		817335	625773				

<Sample Information>

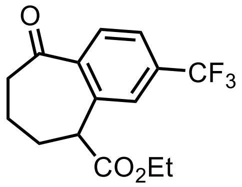

compound **2l**

Sample Name : JUST-286-RG12-neu
Sample ID : JUST-286
Data Filename : JUST-286-RG12-neu.gcd
Method Filename : 50J.gcm
Batch Filename : Batch 1.gcb
Vial # : 5
Injection Volume : 1 uL
Date Acquired : 04.12.2019 19:17:29
Date Processed : 05.12.2019 11:56:36

Sample Type : Unknown
Acquired by : System Administrator
Processed by : System Administrator

<Chromatogram>

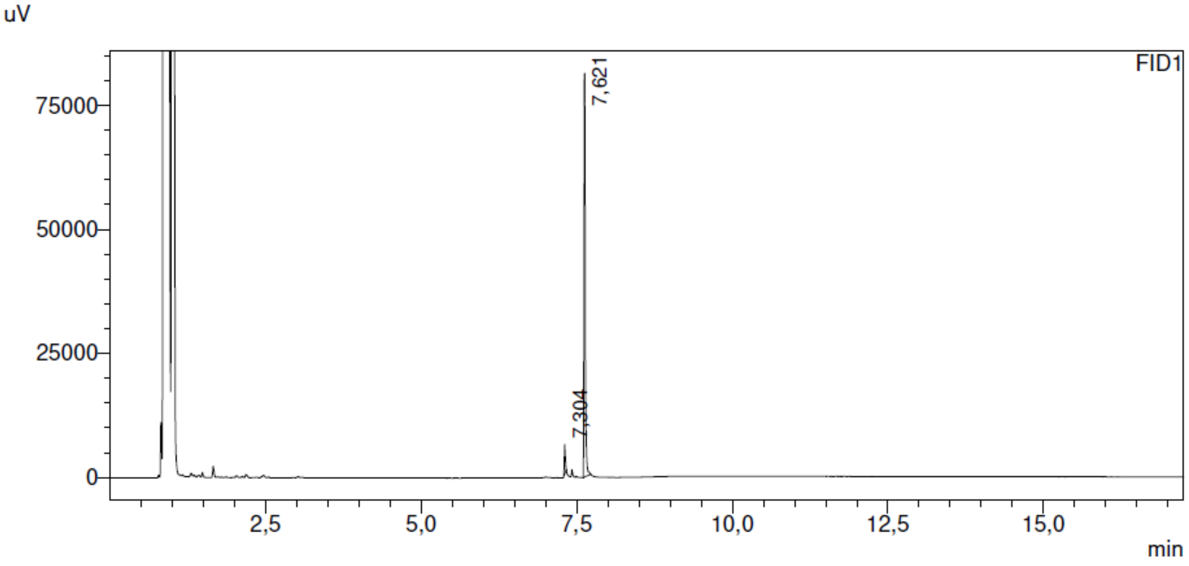

<Peak Table>

FID1

Peak#	Ret. Time	Area	Height	Conc.	Unit	Mark	Name
1	7,304	7529	5647	6,216		M	
2	7,621	113599	77106	93,784		M	
Total		121128	82754				

<Sample Information>

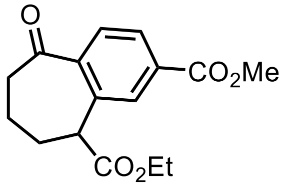

compound **2m**

Sample Name	: Compound_2m
Sample ID	: JUST-292
Data Filename	: Compound_2m.gcd
Method Filename	: 50J.gcm
Batch Filename	: Batch 1.gcb
Vial #	: 3
Injection Volume	: 1 uL
Date Acquired	: 03.12.2019 16:23:10
Date Processed	: 04.12.2019 18:20:29
Sample Type	: Unknown
Acquired by	: System Administrator
Processed by	: System Administrator

<Chromatogram>

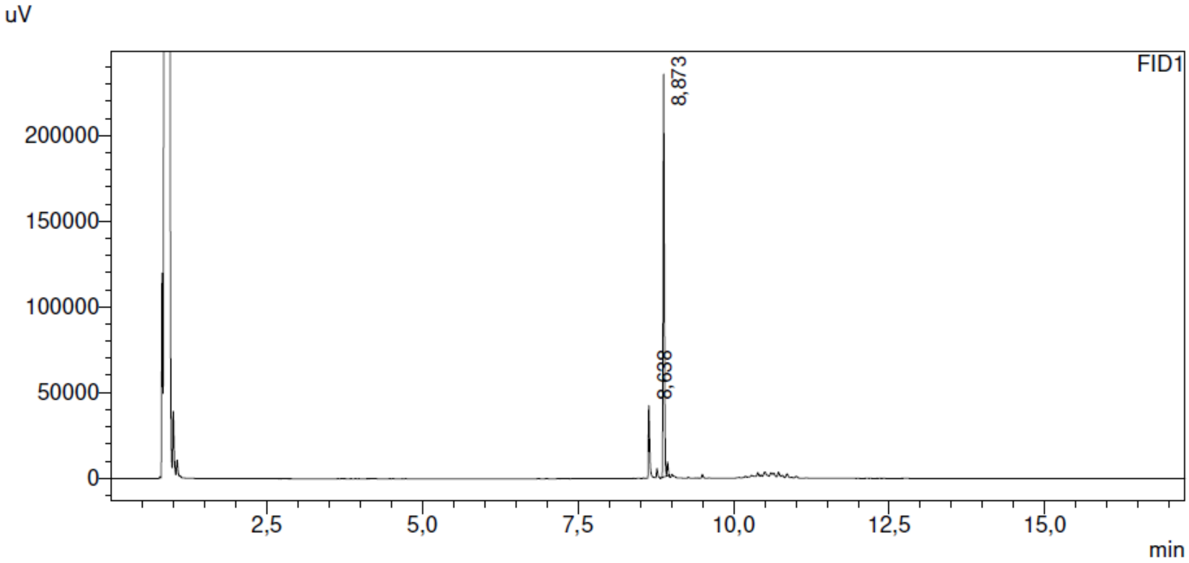

<Peak Table>

FID1

Peak#	Ret. Time	Area	Height	Conc.	Unit	Mark	Name
1	8,638	20508	24399	6,243		M	
2	8,873	308000	227545	93,757		M	
Total		328508	251945				
