# Supplementary figures and images for: Electrochemical‐Induced Ring Transformation of Cyclic α‐(ortho‐Iodophenyl)‐β‐oxoesters
Source: Chemistry. 2020 Jan 30;26(15):3222–5. doi: 10.1002/chem.201905570 (PMC7155071; doi:10.1002/chem.201905570)

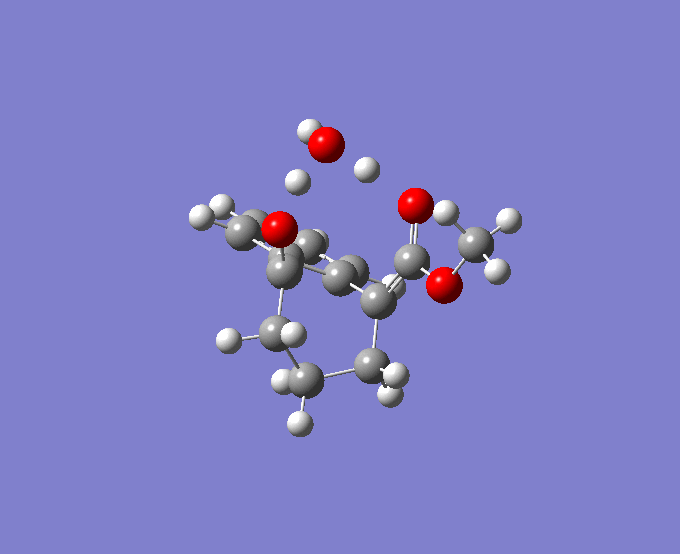

Supplement: Supplementary file 2 — Supplementary [file CHEM-26-3222-s002.gif]
